# Treatment efficacy of anti-hypertensive drugs in monotherapy or combination: ATOM systematic review and meta-analysis of randomized clinical trials according to PRISMA statement

**DOI:** 10.1097/MD.0000000000004071

**Published:** 2016-07-29

**Authors:** Marco A. Paz, Alejandro de-La-Sierra, Marc Sáez, María Antonia Barceló, Juan José Rodríguez, Sonia Castro, Cristina Lagarón, Josep M Garrido, Pilar Vera, Gabriel Coll-de-Tuero

**Affiliations:** a Hospital de Santa Caterina, Girona; b Hospital Mutua Terrassa, University of Barcelona-Terrassa (Barcelona); c Research Group on Statistics, Econometrics and Health (GRECS), University of Girona; d CIBER of Epidemiology and Public Health (CIBERESP); e Department of Medical Sciences, University of Girona; f CAP Can Gibert del Plà, Girona; g Research Unit, USR Girona, IdIAP Gol i Gorina, ICS, Spain.

**Keywords:** antihypertensive agent, hypertension, meta-analysis, PRISMA statement, systemic review

## Abstract

**Background:**

The relative efficacy of antihypertensive drugs/combinations is not well known. Identifying the most effective ones and the patients’ characteristics associated with best performance of the drugs will improve management of hypertensive patients.

**Objective:**

To assess the blood pressure (BP) reduction attributed to antihypertensive drugs and identify characteristics associated with BP decrease.

**Data sources:**

MEDLINE, Cochrane Central Register of Controlled Trials from inception through July 2012 and selected papers.

**Study eligibility criteria:**

Double-blind, randomized clinical trials whose main result was the reduction in BP by antihypertensive treatment, with study population ≥50 or ≥25 if the study was a crossover, follow-up of at least 8 weeks, and available required data.

**Study appraisal and synthesis methods:**

Study data were independently extracted by multiple observers and introduced in an electronic database. Inconsistencies were resolved by discussion and referral back to the original articles. Meta-analysis was performed according to PRISMA statement and using a Bayesian framework.

**Main Outcome(s) and Measure(s):**

Mean decrease in systolic (SBP) and diastolic blood pressure (DBP) achieved by each drug or combination.

**Results:**

Two hundred eight trials including 94,305 patients were identified. In monotherapy, most drugs achieved 10 to 15 mm Hg SBP and 8 to 10 mm Hg DBP decreases.

Olmesartan/amlodipine, olmesartan/hydrochlorothiazide, felodipine/metoprolol, and valsartan/hydrochlorothiazide were the combinations leading to the greatest mean SBP reductions (>20 mm Hg). Female sex and body mass index >25 kg/m^2^ were associated with more pronounced SBP and DBP reductions, whereas Afro-American ethnicity was associated with BP reductions smaller than the median. Results were adjusted by study duration, cardiovascular disease, and diabetes mellitus. Still, the estimation was performed using the mean administered doses, which do not exactly match those of the available drug formats.

**Limitations:**

Data corresponded to those obtained in each of the included trials; the analysis of the combinations was limited to the most recent ones; estimations were performed using the mean administered doses.

**Conclusions and implications:**

Certain drug combinations achieve BP reductions ranging from 20 to 25/10 to 15 mm Hg. Sex, ethnicity, and obesity are associated with antihypertensive response. This information can contribute to better selection of the antihypertensive drug, depending on the magnitude of pretreatment BP elevation. Guidelines should be revised.

## Abstract 2

Supplemental Digital Content is available in the text

## 1 Introduction

Selection of antihypertensive drugs should be based on the knowledge of the drug's ability to reduce blood pressure (BP) levels, which is the main target factor to avoid cardiovascular complications in these patients.^[1]^ Thus, the different treatments have been validated by means of studies showing their antihypertensive efficacy. However, most of these trials have been performed comparing just 2 agents, 2 combinations, or 2 treatment strategies, and they are considerably heterogeneous, with noncomparable study populations with respect to age, sex, and ethnic group, baseline BP or dose.

Therefore, the comparison of the relative antihypertensive effect of several drugs, or that of the most common combinations, is not well known. Moreover, the results obtained with their use, as well as the variables associated with treatment response, differ.^[2–7]^

Although some meta-analyses have been published,^[8,9]^ their ability to determine significant clinical differences among drugs was questioned,^[10]^ since they were restricted to certain pharmacological drugs, and no analyses of combinations were performed or they were performed as simple meta-analyses (i.e., adjusting for specific variables); all of which, made it difficult to generalize the results.

Hypertension (HTN) guidelines recommend antihypertensive drug classes, without detailing specific drugs. As not all drugs from the same class have the same antihypertensive potency, their selection could potentially affect the probability of achieving BP control. Considering the aforementioned explanations, it would be of most importance to know the antihypertensive effect of the most frequently used drugs, adjusted according to the most relevant clinical variables, as well as the characteristics related to better or worse treatment response. This knowledge would potentially help the clinician to choose the most adequate treatment, since the response to a specific drug could be better predicted.

The aim of the “Blood pressure-lowering effects of Anti-hyperTensive drugs and combinations: Meta-regression of published clinical trials” (ATOM study) consisted in determining the BP reduction attributed to the drugs of common use for HTN treatment, adjusted for the most relevant variables in the clinical practice, by means of a meta-analysis. In addition, we aimed to find out whether there were any clinical/phenotypic characteristics associated with the amount of decrease in BP with the use of the specific drug classes.

## 2 Methods

### 2.1 Data sources and searches

A systematic search for clinical trials assessing the efficacy of antihypertensive drugs was conducted following a triple strategy: search in the MEDLINE database with no oldest limit date and up to July 2012, according to the following syntax: “drug's generic name” AND “hypertension” OR “blood pressure” AND “efficacy” with the filters “human”, “randomized clinical trials,” and “English” (complete syntax is shown in supplementary data, Table 1b); search in the Cochrane Central Register of Controlled Trials database with the syntax “drug's generic name” AND “trials” AND “2012”; and review of selected papers aiming to find trials, whose main objective was determining the antihypertensive efficacy, which had not been found with the 2 previous strategies.

### 2.2 Study selection

The initial selection of the studies obtained by the syntax search in the literature databases was assessed by reading the title or the abstract, when doubting the subject of the paper. In this first part, we selected those trials, which could be included, and then we obtained the original papers to be reviewed. The selection of the trials was performed according to the following inclusion criteria: double-blind, randomized clinical trial with a study population ≥50 patients or ≥25 if the study had a crossover design, this minimum patient number was established to reduce as much as possible the heterogeneity of patients included in the analysis; follow-up of at least 8 weeks; and data needed to carry out the meta-analysis had to be available.

The explicit exclusion criteria were: clinical trials conducted exclusively in specific populations of hypertensive patients: diabetic, patients with resistant hypertension or who did not respond to a previous treatment with a specific drug in the same study, chronic kidney disease; clinical trials missing relevant information about the BP reduction obtained or about the dose administered in the different treatment periods; and clinical trials whose main reported outcomes were total mortality, cardiovascular morbidity and mortality, or evolution of the subclinical vascular disease.

### 2.3 Data extraction and quality assessment

Data were extracted and introduced in an electronic database (excel v.2007). Any inconsistencies were resolved by discussion and referral back to the original articles. The following variables were gathered from every original trial and for every arm of treatment: number of included patients; age; sex; ethnicity; baseline systolic (SBP) and diastolic blood pressure (DBP) (mm Hg)—mean and SD values; final SBP and DBP at the end of each period, before titration dose or drug combination; baseline and final heart rate (beats per minute); drug's dose in each study phase; body mass index (BMI); total duration of study (weeks); and presence or absence of diabetes (%).

### 2.4 Data synthesis and analysis

The reductions in SBP and DBP, without eliminating the placebo effect, observed in the various treatments contained in the clinical trials meta-analyzed in this study were combined separately. The reduction in BP attributable to the placebo effect is available in the text. The mean summary combined was the average reduction in SBP and DBP for each drug or combination.

The putative bias risk in the studies included in the meta-analysis was determined by means of the Egger test,^[11]^ which measures the degree of funnel plot asymmetry by the intercept from regression of standard normal deviates against precision. If the *P*-value of the intercept is ≤0.1, the asymmetry is considered to be statistically significant. The *P*-value of the intercept for every pharmacologic group in monotherapy and for the combinations was *P* > 0.10 (supplementary data, Table 2), confirming, thus, the absence of bias in the assessed publications.

Heterogeneity was examined using the *I*^2^ uncertainty parameter, which measures the percentage of variance of the observed results attributable to the heterogeneity.^[12]^ Given the low statistical power of this statistic, much less when, as in our case, the number of studies to be combined is limited, we considered the presence of heterogeneity with statistical significance of 10% and when *I*^2^ exceeded 30%.

Since in all cases we found a very important heterogeneity, we combined the reductions in BP by means of a random effects model.^[13]^ This model allows taking into account both the within (i.e., intrastudy) and between study (i.e., interstudy) heterogeneity. Furthermore, in order to obtain more homogeneous studies in every subgroup, we conducted subgroups analyses stratifying according to drug class and/or dose (low, medium, high) for every active ingredient (supplementary data, Table 3). Finally, we used meta-regression to control heterogeneity introducing potential explanatory variables of the heterogeneity.

The variables initially introduced were the baseline BP (previous to the intervention), dose (one variable in the case of monotherapy, two in the case of combination), duration of treatment, percentage of women, age (standardized), BMI (standardized), percentage of Caucasian individuals, percentage of Afro-American/Afro-Caribbean individuals, and number of treated individuals. However, in those subgroups, in which the number of studies was less than 10, the scarcity of degrees of freedom led to the impossibility to estimate the model in some cases and in others, although the estimation was possible, it was very inefficient (i.e., very wide confidence intervals). In summary, in a third of the cases, when we stratified by pharmacologic groups, and in a half of the cases, when we stratified by active ingredient and dosage, we could not control the heterogeneity using a meta-regression.

For this reason, a sensitivity analysis was performed to determine the minimum number of variables to control that, in turn, would allow controlling the heterogeneity in the majority of cases. In the end, only subgroups with 7 or more studies were combined using meta-regression. The variables finally included in the meta-regression were baseline BP, dose (one variable in the case of monotherapy, two in the case of combination), age (standardized), and number of treated individuals.

This meta-analysis was performed in accordance with the Meta-Analysis of Observational Studies in Epidemiology (MOOSE)^[14]^ and the Preferred Reporting Items for Systematic Reviews and Meta-Analysis (PRISMA) guidelines.^[15]^ Due to the greater flexibility of the Bayesian estimation, a consequence of its hierarchical strategy, we chose to estimate the meta-regressions by means of a Bayesian framework. All analyses were conducted using the free software R (version 3.0.3),^[16]^ through the INLA library.^[17,18]^

### 2.5 Ethical considerations

The variables recorded come from clinical trials that do not contain any personal data. For this reason the approval of an ethics committee was not considered necessary.

## 3 Results

Two hundred eight trials (supplementary data, Table 4) out of the originally reviewed 779 were included. For each study, the following data were gathered: reference, study size, age, baseline SBP and DBP (mean ± SD), antihypertensive drug, and dose.

The other 587 (supplementary data, Table 5) were excluded, since they did not meet the previously stated inclusion criteria or met some of the exclusion criteria.

**Figure 1:**
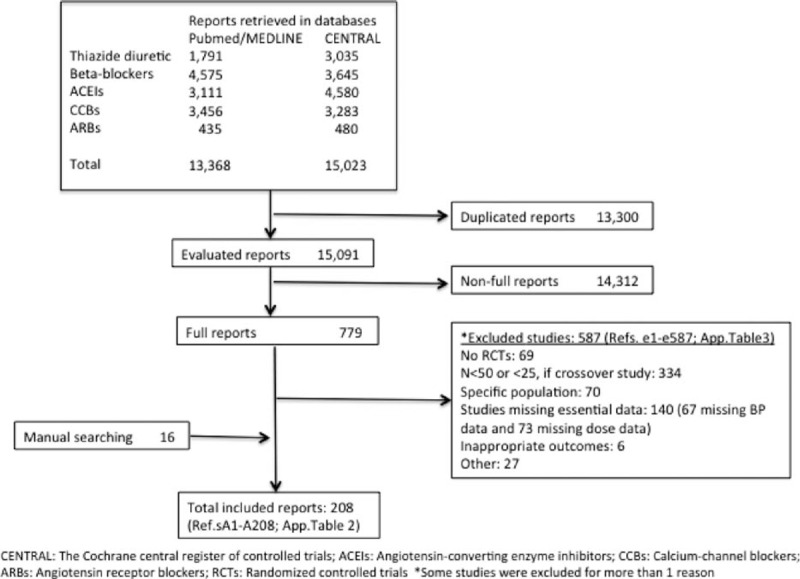
PRISMA flow diagram. Summary of literature search and selection process (For listing of included and excluded full-text articles, see supplemental material Tables 2 and 3).

Figure 1 shows the study flow chart. The total population included consisted of 94,305 patients, whose characteristics are shown in Table 1. The placebo effect was −4.1 mm Hg (95% CI −3.5 to −4.6) for SBP in 94 treatment arms for which this information was available, and −3.5 mm Hg (95% CI −2.6 to −4.4) for DBP in 96 treatment arms.

**Table 1:**
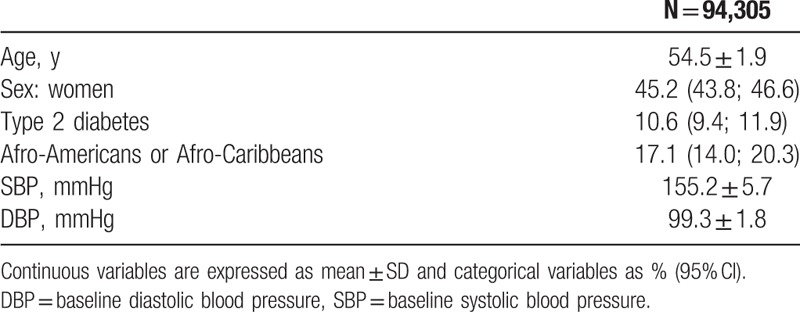
Characteristics of total patients included.

It was not possible to include the variable sex in the meta-regression or the variable age in the multivariate models built to estimate treatment response, since the extremely high collinearity (i.e., correlation between explanatory variables close to the unity) that would cause their inclusion would imply confidence intervals of the coefficient of interest with limits close to infinity, making statistical inference impossible.

### 3.1 Antihypertensive efficacy of specific antihypertensive drugs used as monotherapy

**Figure 2:**
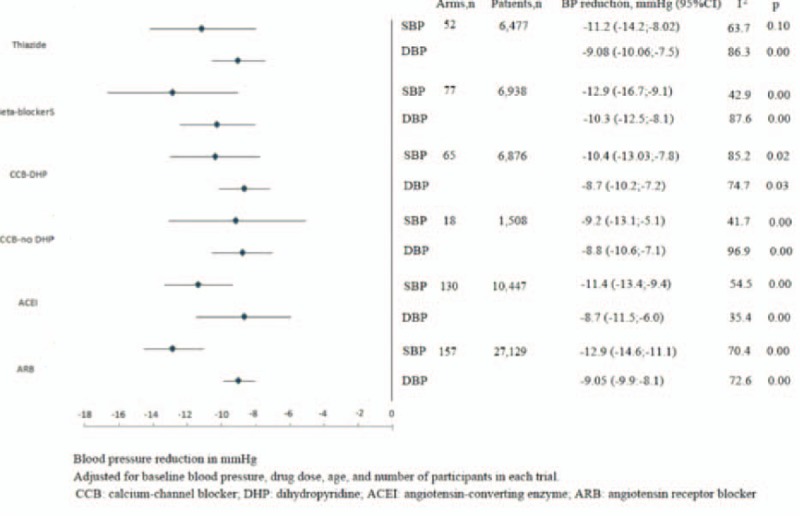
Blood pressure reduction with antihypertensive pharmacologic families in monotherapy: meta-regression analysis.

**Figure 3:**
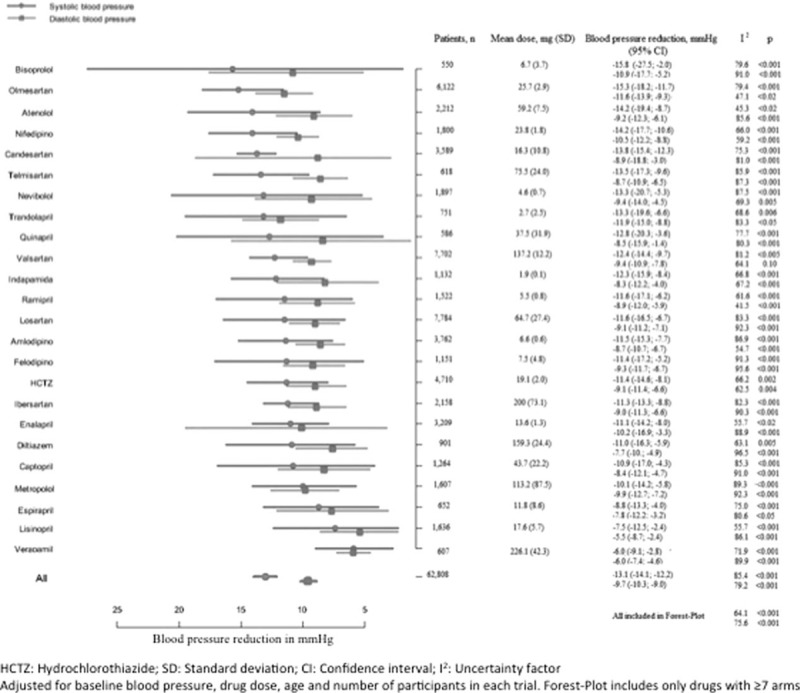
Blood pressure reduction with antihypertensive drugs in monotherapy: meta-regression analysis.

Although decreases in BP were overall similar among the different pharmacologic families (Fig. 2), the specific analysis of the drugs used in monotherapy showed relevant differences (Fig. 3). When considering the BP reduction achieved by drugs on monotherapy at mean doses, we observed that most drugs achieved mean SBP reductions between 10 and 15 mm Hg, while only 2 drugs showed a reduction smaller than 10 mm Hg (lisinopril, −7.5, 95% CI −2.4 to −12.5; and verapamil, −6.0, 95% CI −2.8 to −9.1), and 2 other showed reductions over 15 mm Hg (bisoprolol, −15.8, 95% CI −2 to −27.5; and olmesartan, −15.3; 95% CI −11.7 to −18.2), even though the 95% CI with bisoprolol was considerably wider. Regarding the reduction in mean DBP observed with monotherapy, most drugs resulted in a 5 to 10 mm Hg decrease (hydrochlorothiazide (HCTZ), indapamide, atenolol, metoprolol, nebivolol, amlodipine, felodipine, verapamil, diltiazem, captopril, ramipril, enalapril, lisinopril, spirapril, quinapril, losartan, valsartan, irbesartan, candesartan, telmisartan), while nifedipine, enalapril, trandolapril, olmesartan, and bisoprolol resulted in a 10 to 14 mm Hg reduction.

### 3.2 Antihypertensive efficacy of drug combinations

**Figure 4:**
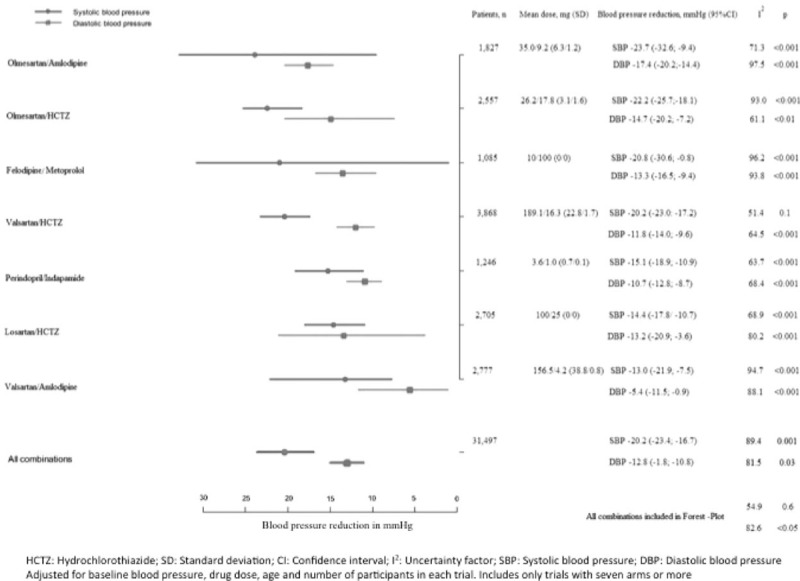
Blood pressure reduction with antihypertensive drug combinations: meta-regression analysis.

Although the mean SBP reduction of the studied combinations was −20.2 (−16.7 to −23.4), the combinations of valsartan/amlodipine, losartan/HCTZ and perindopril/indapamide showed much smaller reductions. On the other hand, the combinations of olmesartan/amlodipine, olmesartan/HCTZ, felodipine/metoprolol, and valsartan/HCTZ achieved SBP decreases larger than 20 mm Hg. The mean DBP decrease was −12.8 (−1.8 to −10.8). All of the studied combinations reduced DBP over 10 mm Hg with the exception of valsartan/amlodipine (−5.4, −0.9 to −11.5). Only the combination of olmesartan/amlodipine reduced DBP more than 15 mm Hg (OR −17.4, −14.4 to −20.2) (Fig. 4).

### 3.3 Characteristics of patients with greater or smaller reductions in BP

Overall, female sex and BMI higher than 25 kg/m^2^ were associated with reductions in SBP/DBP larger than the median reduction (−13.0/−9.8 mm Hg; SBP interquartile range (IQR) 6.05/DBP IQR 3.57), while the Afro-American ethnicity was associated with a reduction in BP smaller than the median reduction.

With respect to different antihypertensive drug classes, women showed more pronounced BP reductions with thiazide diuretics (OR 1.04, 95% CI 1.02–1.06), Angiotensin receptor blockers (ARBs) (1.04, 1.02–1.07) and with drug combinations (1.06, 1.03–1.08; median BP reduction: −19.5/−13.2 mm Hg; SBP IQR 8.7/DBP IQR 4.5), as compared to men.

The increase in BMI was associated with a greater reduction in BP with ARBs (OR for each unit above 25 kg/m^2^, 1.06, 1.02–1.11), calcium-channel blockers (CCBs) (1.05, 1.01–1.11), and drug combinations (1.05, 1.02–1.08), as compared to patients with normal BMI. The Afro-American ethnicity showed smaller reductions in BP when treated with beta-blockers (OR 0.92, 0.84–0.99), as compared to other ethnicities (Table 2).

**Table 2:**
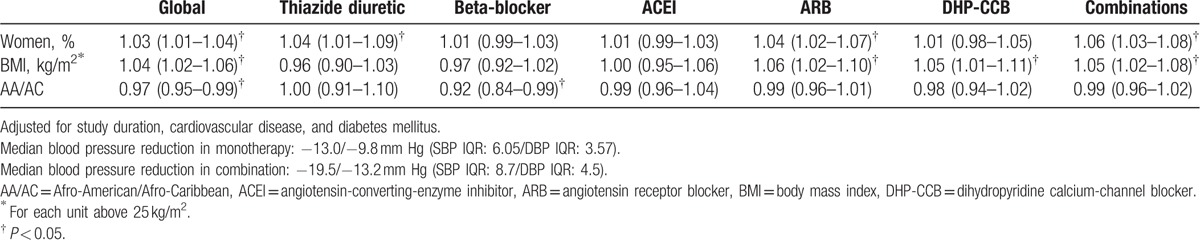
Characteristics of patients with blood pressure reduction above the systolic and diastolic blood pressure median reductions: multivariate analysis [OR (95% CI)].

## 4 Discussion

Our study allows assessing the BP reduction achieved with the most frequently used drugs for HTN treatment by means of Bayesian meta-regression, and the results may be applied to a wide population, independently of dose, baseline BP, and age.

Main results of our meta-regression partly agree with the recommendations of the European Society of Hypertension and the European Society of Cardiology (ESH/ESC) guidelines 1 and with the report by the Eighth Joint National Committee (JNC-8),^[19]^ in that reductions obtained with monotherapy are limited. In our study, those values are 20/10 mm Hg, and in order to achieve greater decreases in BP than these values, the use of drug combinations should be recommended. These results provide evidence supporting the recommendations of the aforementioned guidelines, which were mostly derived from expert opinions. In addition, the NICE guidelines recommend a stepwise pharmacological approach without initiating the combination treatment.^[20]^ This possibility is also provided by the ESC guidelines but note that only a few patients will achieve a decrease in BP large enough and that most patients will need a drug combination.

There are only few studies comparing the efficacy of the most commonly used antihypertensive drugs. The current meta-analysis shows similar results to those of Baguet et al,^[8,9]^ regarding HCTZ, amlodipine, and enalapril, and different BP reductions for atenolol, lisinopril, verapamil, and diltiazem. However, those studies were performed as simple meta-analyses, with no meta-regression, and results were only adjusted by the number of subjects included in each assay, without taking into account other important variables such as the BP at the time of study inclusion.

Additionally, our study shows variability in the strength of the different antihypertensive drugs to lower SBP, as well as DBP. These differences are important and may reach 9.3 and 6.0 mm Hg for SBP and DBP, respectively, when comparing the most and the least efficacious drugs. In the case of the studied drug combinations, these differences are greater, at 14.6 and 13.1 mm Hg for SBP and DBP, respectively.

Although a similar antihypertensive effect of most drug classes was observed, the differences among specific drugs from the same class should probably be kept in mind for treatment selection. A general recommendation of a drug class could eventually be translated into the recommendation of a specific drug, which would not be able to achieve the therapeutic goal, even with its best response.

Compared to other agents, the smallest BP reductions observed with verapamil and lisinopril stand out in the evaluation of treatment responses according to drug. It must be pointed out, as it has been previously reported, that as compared to enalapril, the dose of lisinopril resulting in an equivalent BP reduction is about double.^[19]^ On the other hand, no differences between indapamide and HCTZ were observed in our study, despite some meta-analyses showing the superiority of indapamide.^[8,9]^ In the same context, the drugs showing the greatest efficacy were bisoprolol and olmesartan, although with very different confidence intervals. The wider confidence interval of bisoprolol implies large variability in the individual response. In contrast, olmesartan shows a much narrower confidence interval translating into a more predictable clinical response to this drug. Our data are in agreement with those of a meta-analysis conducted with 4892 patients showing that olmesartan has greater efficacy in BP reduction than losartan and valsartan.^[21]^

Overall, any of the assessed drug combinations guarantees SBP reductions >20 mm Hg and DBP reductions >10 mm Hg; and thus, they may be considered as the first option treatment when a reduction in BP of such magnitude is required. However, some combinations achieve greater BP reductions than others and this can also be taken into account in the process of drug selection. The reduction in BP varies depending on certain phenotypic characteristics of the population, such as sex, BMI, and ethnicity.

When the characteristics of the patients showing the overall best antihypertensive response were assessed, the associated variables were female sex, higher BMI, and Caucasian ethnicity, whereas Afro-American ethnicity and normal weight were associated with a lower response to antihypertensive drugs. Women experienced better antihypertensive response overall, and specifically to thiazides, ARBs, and combinations. Agarwal et al^[5]^ also showed that women had better antihypertensive response to combinations (both, CCBs plus olmesartan and thiazide diuretics plus olmesartan) than men.

Regarding obesity, Weber et al^[7]^ showed greater cardiovascular morbidity and mortality in obese patients treated with benazepril/HCTZ as compared to patients with normal weight; and these differences disappeared when patients were treated with benazepril/amlodipine. The results of the current meta-regression show an intense association between BMI above 25 kg/m^2^ and overall BP reduction, and between BMI above 25 kg/m^2^ and BP reduction with CCBs, ARBs, and combinations.

The recent meta-analysis published by the Blood Pressure Lowering Treatment Trialists’ Collaboration shows that angiotensin-converting-enzyme inhibitors (ACEIs) are more effective than CCBs in reducing the cardiovascular risk of patients with high BMI.^[22]^

The authors did not report data on ARBs in the same situation. Our study shows neutral ACEIs behavior regarding BP reduction according to BMI. This result does not disagree with those of the mentioned meta-analysis since at the same BP reduction, the ACEIs may lead to a greater reduction in cardiovascular risk by means of other physiopathologic mechanisms.

With respect to ethnicity, the use of diuretics or CCBs has been recommended in Afro-American patients.^[1,19,23,24]^ A smaller response of these patients to beta-blockers and to ACEIs/ARBs, as well as a greater BP reduction with diuretics and CCBs, has been reported.^[2,5,6]^ The meta-regression shows an overall response lower than the median in Afro-American patients treated with beta-blockers as compared to other ethnicities.

However, a smaller BP decrease with ACEIs/ARBs was not observed, neither an improved response to thiazides or CCBs. These results do not support the recommendations of NICE,^[20]^ ESH,^[1]^ or JNC-8^[19]^ guidelines.

Our data show that overweight patients may especially benefit from using drug combinations of antihypertensive drugs, since they respond better to this type of treatment, showing a hypotensive response 5% greater than the median per every increased BMI unit with respect to those with normal BMI, achieving in obese patients (BMI >30 kg/m^2^) a hypotensive response greater than 25%. This observation should be considered for the treatment of these patients.

It is usually recommended to initiate antihypertensive therapy with drug combinations, not only in grade 2 hypertension but also in subjects with high cardiovascular risk associated with the presence of multiple risk factors, chronic kidney disease, or subclinical vascular disease. According to the results of this meta-analysis, this recommendation should probably be extended to obese subjects.

The study has some limitations: first, data are not individual but correspond to those obtained in each of the included trials; second, the analysis of the combinations is limited to the most recent ones, most of them with ARBs, since we could not find enough studies performed with other commonly used combinations such as ACEIs (enalapril or lisinopril) with HCTZ; and, finally, the estimation was performed using the mean administered doses, which do not exactly match those of the available drug formats, although they are very close.

In summary, our study shows that the expected mean BP reduction in monotherapy is overall between 10 and 15 mm Hg in SBP and between 8 and 10 mm Hg in DBP. The use of drug combinations at average/high doses achieves BP reductions ranging from 20 to 25/10 to 15 mmHg. Reductions in SBP/DBP higher than 20/10 mmHg are very unlikely to be achieved with monotherapy.

## 5 Conclusion

Even with the assumption that all drug classes promote similar BP reductions, clinically relevant differences exist among specific drugs. This should be reflected in hypertension guidelines, since a general drug class recommendation could eventually promote the use of a specific drug with not enough potency to achieve therapeutic goals.

On the other hand, it is possible to identify phenotypic variables such as sex, ethnicity, or obesity associated with the antihypertensive response. This knowledge can contribute to a finer adjustment of the recommendations stated in the managing guidelines for this condition. In addition, it might justify future studies oriented to facilitate treatment individualization, taking into consideration these variables together with the cardiovascular risk profile of the patient and the presence or not of subclinical vascular disease.

## Acknowledgments

We thank Almudena Pardo Mateos who translated the manuscript and formatted it for publication.

## Supplementary Material

Supplemental Digital Content
