# Supplementary material for: Treatment efficacy of anti-hypertensive drugs in monotherapy or combination: ATOM systematic review and meta-analysis of randomized clinical trials according to PRISMA statement
Source: Medicine (Baltimore). 2016 Jul 29;95(30):e4071. doi: 10.1097/MD.0000000000004071 (PMC5265817; doi:10.1097/MD.0000000000004071)
Supplement: Supplemental Digital Content [file medi-95-e4071-s001.doc]

Supplementary Table 1. Complete search Syntax in PubMed

| ("Drug generic name" [MeSH Terms] OR " Drug generic name" [All Fields] OR " Drug generic name" [Pharmacological Action]) AND (("blood pressure"[MeSH Terms] OR ("blood"[All Fields] AND "pressure"[All Fields]) OR "blood pressure"[All Fields] OR "blood pressure determination"[MeSH Terms] OR ("blood"[All Fields] AND "pressure"[All Fields] AND "determination"[All Fields]) OR "blood pressure determination"[All Fields] OR ("blood"[All Fields] AND "pressure"[All Fields]) OR "blood pressure"[All Fields] OR "arterial pressure"[MeSH Terms] OR ("arterial"[All Fields] AND "pressure"[All Fields]) OR "arterial pressure"[All Fields] OR ("blood"[All Fields] AND "pressure"[All Fields])) OR ("hypertension"[MeSH Terms] OR "hypertension"[All Fields])) NOT (("death"[MeSH Terms] OR "death"[All Fields]) OR ("complications"[Subheading] OR "complications"[All Fields])) with activated filters “Human” [MeSH terms], “Clinical Trial” or “Randomized Controlled Trial” [ptyp] , English [lang] |
| --- |

**Supplementary data, table 2.Egger test.**

The null hypothesis is that the intercept from the regression of standard normal deviates (dependent variable) vs. precision (explicative variable), pondered by thevariance is equal to zero (NO publication bias)

**Table 2**. p-values of the intercept (Student´s t test)

| **Pharmacologic group** | **SBP** | **DBP** |
| --- | --- | --- |
| **Thiazide diuretics** | 0.731 | 0.331 |
| **Beta-blockers** | 0.391 | 0.205 |
| **Dihydropyridine (DHP) calcium channel blockers** | 0.754 | 0.305 |
| **Non-DHP calcium channel blockers** | 0.276 | 0.282 |
| **Angiotensin-converting enzyme-inhibitors (ACEIs)** | 0.204 | 0.196 |
| **Angiotensin II receptor blockers (ARBs)** | 0.658 | 0.342 |
| **All -Monotherapy** | 0.123 | 0.191 |
| **Drug** | **SBP** | **DBP** |
| **Indapamide (r)** | 0.272 | 0.656 |
| **Hydrochlorothiazide** | 0.216 | 0.291 |
| **Atenolol** | 0.745 | 0.179 |
| **Bisoprolol** | 0.547 | 0.797 |
| **Metroprolol** | 0.163 | 0.122 |
| **Nevibolol** | 0.372 | 0.189 |
| **Amlodipine** | 0.801 | 0.722 |
| **Felodipine** | 0.417 | 0.636 |
| **Lecardinipine** | 0.296 | 0.328 |
| **Nifedipine** | 0.753 | 0.179 |
| **Diltiazem** | 0.220 | 0.193 |
| **Verapamil** | 0.545 | 0.427 |
| **Captopril** | 0.367 | 0.766 |
| **Enalapril** | 0.401 | 0.194 |
| **Lisinopril** | 0.609 | 0.956 |
| **Quinapril** | 0.333 | 0.276 |
| **Ramipril** | 0.589 | 0.635 |
| **Espirapril** | 0.625 | 0.268 |
| **Trandolapril** | 0.163 | 0.162 |
| **Candesartan** | 0.417 | 0.560 |
| **Ibersartan** | 0.622 | 0.822 |
| **Losartan** | 0.713 | 0.893 |
| **Olmesartan** | 0.981 | 0.174 |
| **Telmisartan** | 0.770 | 0.905 |
| **Valsartan** | 0.422 | 0.186 |
| **Combinations** | **SBP** | **DBP** |
| **Felodipine/Metropolol** | 0.390 | 0.249 |
| **Losartan/HCTZ** | 0.689 | 0.297 |
| **Olmesartan/Amlodipine** | 0.405 | 0.746 |
| **Olmesartan/HCTZ** | 0.430 | 0.986 |
| **Perindopril/Indapamide** | 0.388 | 0.520 |
| **Valsartan/Amlodipino** | 0.953 | 0.142 |
| **Valsartan/HCTZ** | 0.880 | 0.719 |
| **Total - Combination** | 0.511 | 0.373 |
| SBP: Systolic blood pressure; DBP: Diastolic blood pressure; DHP: Dihydropyridine; HCTZ: hydrochlorothiazide | | |

**Supplementary Table 3. Dose (mg) of antihypertensive drugs**

| **Drug** | **Minimum** | **Mean** | **Maximum** |
| --- | --- | --- | --- |
| **Diuretics** |  |  |  |
| Chlorthalidone | 12.5 | 25 | 50 |
| Indapamide | 1.25 | 2.5 | 5 |
| Hydrochlorothiazide | 12.5 | 25 | 50 |
| **Beta-blockers** |  |  |  |
| Atenolol | 25 | 50 | 100 |
| Bisoprolol | 2.5 | 5-10 | 20 |
| Nebivolol | 2.5 | 5 | 5 |
| **ACEI** |  |  |  |
| Enalapril | 5 | 10-20 | 40 |
| Lisinopril | 10 | 20-40 | 80 |
| Ramipril | 2.5 | 5 | 10 |
| **ARB** |  |  |  |
| Olmesartan | 10 | 20 | 40 |
| Telmisartan | 40 | 40-80 | 80 |
| Valsartan | 80 | 160 | 320 |
| **CCB** |  |  |  |
| Amlodipine | 5 | 5-10 | 10 |
| Lercanidipine | 10 | 10-20 | 20 |
| Nifedipine | 30 | 60 | 120 |
| Diltiazem | 120 | 180-240 | 360 |
| Verapamil | 120 | 240 | 480 |
| **Combinations** |  |  |  |
| Perindopril/Indapamide | 2/0.75 | 4/1.5 |  |
| Olmesartan+HCTZ |  | 20/12.5 | 40/12.5-25 |
| Olmesartan/amlodipine |  | 20/5 | 40/10 |
| Valsartan/HCTZ |  | 160/12.5 |  |
| Valsartan/amlodipine |  | 160/5 | 160/10 |
| Felodipine/metoprolol |  | 5/50 | 10/100 |
| Losartan/HCTZ |  | 50/12.5 | 100/25 |
| ACEI: angiotensin-converting enzyme inhibitor; ARB: angiotensin receptor blocker; CCB: calcium-channel blocker; HCTZ: hydrochlorothiazide | | | |

Supplementary Table 4. Included studies

| **Author** | **Reference** | **Participants** | **Age (mean)** | **Age SD** | **Baseline SBP** | **SD Baseline SBP** | **Baseline DBP** | **SD Baseline DBP** | **Drug 1** | **Drug 2** | **Drug 3** | **Drug 1 dose** | **Drug 2 dose** | **Drug 3 dose** | **Daily intakes** |
| --- | --- | --- | --- | --- | --- | --- | --- | --- | --- | --- | --- | --- | --- | --- | --- |
| Benz JR. Et al J Hum Hypertens. 1998 | A001 | 94 | 52 | 10.4 | 152.7 | 17.1 | 101.4 | 5 | placebo |  |  | 0 |  |  | 1 |
|  | A001 | 99 | 52 | 10.2 | 153.7 | 14.4 | 101.5 | 4.9 | valsartan |  |  | 80 |  |  | 1 |
|  | A001 | 99 | 52 | 10.5 | 153.5 | 15.1 | 101.5 | 4.8 | valsartan |  |  | 160 |  |  | 1 |
|  | A001 | 100 | 52 | 11.4 | 153.6 | 16.4 | 101.2 | 4.5 | HCTZ |  |  | 12.5 |  |  | 1 |
|  | A001 | 100 | 52 | 11 | 152 | 15.5 | 100.8 | 4.6 | HCTZ |  |  | 25 |  |  | 1 |
|  | A001 | 96 | 52 | 11.9 | 153 | 14.4 | 101 | 4.9 | valsartan | HCTZ |  | 80 | 12.5 |  | 1 |
|  | A001 | 97 | 53 | 11.3 | 154.5 | 15.4 | 101 | 4.5 | valsartan | HCTZ |  | 80 | 25 |  | 1 |
|  | A001 | 92 | 51 | 11.2 | 152 | 14.2 | 100.4 | 4.6 | valsartan | HCTZ |  | 160 | 12.5 |  | 1 |
|  | A001 | 94 | 53 | 11.2 | 155.9 | 14.8 | 101.4 | 4.8 | valsartan | HCTZ |  | 160 | 25 |  | 1 |
| Chrysant SG. Et al Am J Hypertens. 2004 | A002 | 42 | 54 | 9.9 | 152.1 |  | 103.4 |  | placebo |  |  | 0 |  |  | 1 |
|  | A002 | 45 | 54.1 | 10.8 | 153.4 |  | 103 |  | HCTZ |  |  | 12.5 |  |  | 1 |
|  | A002 | 43 | 54.7 | 10.5 | 155.9 |  | 104.4 |  | HCTZ |  |  | 25 |  |  | 1 |
|  | A002 | 39 | 49.9 | 10.9 | 153.6 |  | 104.1 |  | olmesartan |  |  | 10 |  |  | 1 |
|  | A002 | 41 | 54.1 | 9.9 | 154.6 |  | 103.2 |  | olmesartan |  |  | 20 |  |  | 1 |
|  | A002 | 45 | 54.4 | 11.2 | 152.9 |  | 102.6 |  | olmesartan |  |  | 40 |  |  | 1 |
|  | A002 | 35 | 52.4 | 10.7 | 156.6 |  | 104 |  | olmesartan | HCTZ |  | 10 | 12.5 |  | 1 |
|  | A002 | 39 | 54.4 | 11.7 | 153.7 |  | 103.8 |  | olmesartan | HCTZ |  | 10 | 25 |  | 1 |
|  | A002 | 44 | 52.3 | 10.2 | 152.3 |  | 103.1 |  | olmesartan | HCTZ |  | 20 | 12.5 |  | 1 |
|  | A002 | 47 | 51.6 | 14 | 154.7 |  | 103.8 |  | olmesartan | HCTZ |  | 20 | 25 |  | 1 |
|  | A002 | 42 | 52 | 10.5 | 151.9 |  | 103.9 |  | olmesartan | HCTZ |  | 40 | 12.5 |  | 1 |
|  | A002 | 40 | 51.7 | 11.6 | 153.6 |  | 103.4 |  | olmesartan | HCTZ |  | 40 | 25 |  | 1 |
| Myers MG. Et al J Hypertens. 2000 | A003 | 61 | 56 |  | 164 |  | 102 |  | placebo |  |  | 0 |  |  | 1 |
|  | A003 | 65 | 55 |  | 163 |  | 102 |  | perindopril | indapamida |  | 2 | 0.625 |  | 1 |
|  | A003 | 60 | 56 |  | 161 |  | 101 |  | indapamida |  |  | 1.25 |  |  | 1 |
|  | A003 | 65 | 54 |  | 159 |  | 101 |  | perindopril | indapamida |  | 2 | 1.25 |  | 1 |
|  | A003 | 61 | 54 |  | 160 |  | 101 |  | perindopril | indapamida |  | 4 | 1.25 |  | 1 |
|  | A003 | 64 | 57 |  | 164 |  | 102 |  | perindopril | indapamida |  | 8 | 1.25 |  | 1 |
|  | A003 | 62 | 55 |  | 162 |  | 101 |  | perindopril | indapamida |  | 8 | 2.5 |  | 1 |
| Bichisao E. et al J Internat Med Res.1989 | A004 | 274 | 51.1 | 10.9 | 169 |  | 102.5 |  | clortalidona |  |  | 25 |  |  | 1 |
|  | A004 | 271 | 51.8 | 9.5 | 170 |  | 103 |  | clortalidona | metoprolol |  | 25 | 200 |  | 1 |
| Chrysant SG. Et al . J Clin Pharmacol. 1992 | A005 | 43 | 53 | 2 | 153 |  | 104 |  | placebo |  |  | 0 |  |  | 1 |
|  | A005 | 41 | 51 | 2 | 152 |  | 103 |  | hidroclorotiazida | Triamterene |  | 25 | 50 |  | 1 |
|  | A005 | 44 | 55 | 2 | 154 |  | 103 |  | atenolol |  |  | 25 |  |  | 1 |
|  | A005 | 42 | 54 | 1 | 151 |  | 103 |  | atenolol |  |  | 50 |  |  | 1 |
|  | A005 | 43 | 53 | 2 | 150 |  | 102 |  | atenolol | HCTZ | Triamterene | 25 | 25 | 50 | 1 |
|  | A005 | 43 | 54 | 2 | 153 |  | 103 |  | atenolol | HCTZ | Triamterene | 50 | 25 | 50 | 1 |
| Papademetriou V. et al Am J Hypertens. 2006 | A006 | 86 | 54 | 12 | 152 |  | 100 |  | hidroclorotiazida |  |  | 6.25 |  |  | 1 |
|  | A006 | 104 | 54 | 11 | 151 |  | 100 |  | hidroclorotiazida |  |  | 12.5 |  |  | 1 |
|  | A006 | 48 | 53 | 9 | 151 |  | 100 |  | hidroclorotiazida |  |  | 25 |  |  | 1 |
|  | A006 | 152 | 53 | 12 | 151 |  | 100 |  | placebo |  |  | 0 |  |  | 1 |
| Genthon R. et al Int J Clin Pharm Res. 1994 | A007 | 185 | 54.5 | 0.84 | 166.7 |  | 102.2 |  | ramipril |  |  | 2.5 |  |  | 1 |
|  | A007 | 183 | 54.2 | 0.87 | 167.9 |  | 102.9 |  | hidroclorotiazida |  |  | 12.5 |  |  | 1 |
|  | A007 | 167 | 56 | 0.84 | 167.5 |  | 102.1 |  | ramipril | HCTZ |  | 2.5 | 12.5 |  | 1 |
| Thijs L. et al . J Cardiovasc Pharmacol.1995 | A008 | 209 | 53 | 10 | 164 | 17 | 104 | 6 | ramipril |  |  | 5 |  |  | 1 |
|  | A008 | 201 | 57 | 10 | 164 | 18 | 104 | 7 | ramipril | Piretanida |  | 5 | 6 |  | 1 |
| Guthrie R. et al Am J Hypertens. 1996 | A009 | 30 | 52 | 9.9 | 152 | 15.4 | 101 | 4.3 | placebo |  |  | 0 |  |  | 1 |
|  | A009 | 31 | 53 | 10.6 | 149 | 13.9 | 100 | 5 | placebo |  |  | 0 |  |  | 1 |
| Nash DT. Et al Current Med Res Opin. 2008 | A010 | 258 | 52.2 | 10.8 | 151.2 | 14.4 | 100 | 4.4 | placebo |  |  | 0 |  |  | 1 |
|  | A010 | 267 | 52.6 | 11.8 | 152.2 | 14.2 | 100.1 | 4.2 | hidroclorotiazida |  |  | 12.5 |  |  | 1 |
|  | A010 | 264 | 52.9 | 11.2 | 150.8 | 13.7 | 99.6 | 3.9 | hidroclorotiazida |  |  | 25 |  |  | 1 |
|  | A010 | 263 | 52.2 | 10.9 | 151 | 13.9 | 99.9 | 4 | valsartan |  |  | 160 |  |  | 1 |
|  | A010 | 261 | 53 | 10.6 | 151.6 | 13.5 | 99.8 | 4 | valsartan | HCTZ |  | 160 | 12.5 |  | 1 |
| Chrysant SG. Et al Arch Intern Med. 1994 | A011 | 81 | 53 |  | 155 |  | 103 |  | placebo |  |  | 0 |  |  | 1 |
|  | A011 | 85 | 54 |  | 154 |  | 104 |  | lisinopril |  |  | 10 |  |  | 1 |
|  | A011 | 87 | 54 |  | 155 |  | 103 |  | hidroclorotiazida |  |  | 12.5 |  |  | 1 |
|  | A011 | 84 | 53 |  | 155 |  | 104 |  | hidroclorotiazida |  |  | 25 |  |  | 1 |
|  | A011 | 85 | 51 |  | 152 |  | 104 |  | lisinopril | HCTZ |  | 10 | 12.5 |  | 1 |
|  | A011 | 83 | 53 |  | 154 |  | 104 |  | lisinopril | HCTZ |  | 10 | 25 |  | 1 |
| Kayanakis JG. Et al Br J Clin Pharmac. 1987 | A012 | 83 | 52.8 | 9 | 172 |  | 102.5 |  | placebo |  |  | 0 |  |  | 1 |
|  | A012 | 41 | 55.1 | 10.7 | 176.6 |  | 103.2 |  | hidroclorotiazida |  |  | 25 |  |  | 1 |
| Persson B. et al Eur J Clin Pharmacol.1996 | A013 | 48 | 70.7 |  | 172 |  | 103 |  | placebo |  |  | 0 |  |  | 1 |
|  | A013 | 50 | 70 |  | 171 |  | 102 |  | hidroclorotiazida |  |  | 25 |  |  | 1 |
| Izzo JL Jr. Et al . J Clin Hypertens (Greenwich). 2011 | A014 | 112 | 57.9 | 11.4 | 169.5 | 14.7 | 100.4 | 7.7 | olmesartan | amlodipino |  | 40 | 10 |  | 1 |
|  | A014 | 116 | 55.8 | 9.2 | 166.3 | 13 | 100.9 | 8 | olmesartan | HCTZ |  | 40 | 25 |  | 1 |
|  | A014 | 117 | 57.6 | 11.1 | 168.3 | 12.5 | 100.1 | 6.4 | olmesartan | amlodipino | HCTZ | 40 | 10 | 25 | 1 |
| Andrejak M. et al Fundam Clin Pharmacol. 1991 | A015 | 82 | 52.4 |  | 170.8 |  | 105.4 |  | perindopril |  |  | 4 |  |  | 1 |
|  | A015 | 83 | 53.9 |  | 171.1 |  | 105 |  | hidroclorotiazida | amiloride |  | 50 | 5 |  | 1 |
| Hart W. et al J Hum Hypertens. 1991 | A016 | 98 | 71 |  | 177 |  | 107 |  | lisinopril |  |  | 20 |  |  | 1 |
|  | A016 | 97 | 71 |  | 180 |  | 106 |  | lisinopril | HCTZ |  | 20 | 12.5 |  | 1 |
|  | A016 | 104 | 71 |  | 175 |  | 105 |  | HCTZ |  |  | 12.5 |  |  | 1 |
| Edes I. et al Clin Drugs Invest. 2009 | A017 | 92 | 53 | 11 | 153.8 | 11.1 | 97.8 | 5.1 | Placebo |  |  |  |  |  | 1 |
|  | A017 | 486 | 53 | 10 | 154 | 13.1 | 97.5 | 5.6 | Candesartan cilexetil | HCTZ |  | 32 | 25 |  | 1 |
|  | A017 | 457 | 52 | 11 | 152.9 | 12.8 | 97.4 | 5.6 | Candesartan cilexetil |  |  | 32 |  |  | 1 |
|  | A017 | 464 | 51 | 10 | 153.2 | 13.3 | 97.8 | 5.5 | HCTZ |  |  | 25 |  |  | 1 |
| William B. White. J Clin Hypertens (Greenwich). 2006 | A018 | 485 | 54 | 11 | 155 | 12 | 102 | 4 | Telmisartan | HCTZ |  | 80 | 25 |  | 1 |
|  | A018 | 498 | 53 | 10 | 154 | 12 | 102 | 4 | Valsartan | HCTZ |  | 160 | 25 |  | 1 |
|  | A018 | 126 | 53 | 11 | 155 | 13 | 102 | 4 | Placebo |  |  |  |  |  | 1 |
| Romero R. J Cardiovasc Pharmacol. 1995 | A019 | 106 | 53 |  | 168.4 |  | 109.1 |  | quinapril |  |  | 20 |  |  | 1 |
|  | A019 | 108 | 52 |  | 167.4 |  | 109.2 |  | quinapril | HCTZ |  | 20 | 12.5 |  | 1 |
|  | A019 | 109 | 55 |  | 167 |  | 109.1 |  | HCTZ |  |  | 12.5 |  |  | 1 |
| Alan H. Gradman. Clinical Therapeutics. 1999 | A020 | 123 | 54 | 1 | 149.3 | 1.2 | 100.4 | 0.4 | Eprosartan |  |  | 6.25 |  |  | 1 |
|  | A020 | 120 | 53.3 | 0.9 | 151.3 | 1.3 | 101.2 | 0.4 | Placebo |  |  |  |  |  | 1 |
| Pool JL. Et al J Cardiovasc Pharmacol. 1987 | A021 | 162 | 52.9 |  | 148.4 |  | 98.1 |  | lisinopril |  |  | 20 |  |  | 1 |
|  | A021 | 158 | 53.1 |  | 150.7 |  | 98.6 |  | hidroclorotiazida |  |  | 12.5 |  |  | 1 |
|  | A021 | 74 | 53.2 |  | 146.5 |  | 97.5 |  | lisinopril | HCTZ |  | 20 | 12.5 |  | 1 |
| Weissel M. et al J Cardiovasc Pharmacol. 1990 | A022 | 68 | 66.1 | 4.7 | 170 | 14 | 101 | 5 | hidroclorotiazida |  |  | 25 |  |  | 1 |
|  | A022 | 66 | 65.6 | 5.1 | 171 | 16 | 101 | 8 | felodipino |  |  | 5 |  |  | 1 |
| Pareek A. et al Curr Med Res Opin. 2008 | A023 | 98 | 48.49 | 12.98 | 149.47 | 7.69 | 93.23 | 3.62 | atenolol |  |  | 25 |  |  | 1 |
|  | A023 | 100 | 46.44 | 11.79 | 149.43 | 6.99 | 93.81 | 4.33 | clortalidona |  |  | 6.25 |  |  | 1 |
|  | A023 | 102 | 48.98 | 10.83 | 149.66 | 7.20 | 93.50 | 4.48 | amlodipino |  |  | 2.5 |  |  | 1 |
| Neutel JM. Et al J Hum Hypertens. 2008 | A024 | 328 | 55.1 |  | 161.7 |  | 97.5 |  | irbesartan | HCTZ |  | 150 | 12.5 |  | 1 |
|  | A024 | 106 | 55.3 |  | 161.4 |  | 97.9 |  | irbesartan |  |  | 150 |  |  | 1 |
|  | A024 | 104 | 56.0 |  | 162 |  | 97.6 |  | hidroclorotiazida |  |  | 12.5 |  |  | 1 |
| Pordy RC. Et al Cardiology. 1994 | A025 | 94 | 53.0 | 11.9 |  |  | 100.3 |  | placebo |  |  |  |  |  | 1 |
|  | A025 | 97 | 53.9 | 12.1 |  |  | 100.1 |  | cilazapril |  |  | 0.5 |  |  | 1 |
|  | A025 | 94 | 53.9 | 12.1 |  |  | 100.3 |  | cilazapril |  |  | 5 |  |  | 1 |
|  | A025 | 92 | 53.9 | 12.1 |  |  | 100.8 |  | cilazapril |  |  | 10 |  |  | 1 |
|  | A025 | 98 | 54.7 | 11.7 |  |  | 100.9 |  | hidroclorotiazida |  |  | 12.5 |  |  | 1 |
|  | A025 | 95 | 54.7 | 11.7 |  |  | 99.9 |  | hidroclorotiazida |  |  | 25 |  |  | 1 |
|  | A025 | 94 | 53.9 | 12.1 |  |  | 100.2 |  | cilazapril | HCTZ |  | 0.5 | 12.5 |  | 1 |
|  | A025 | 96 | 53.9 | 12.1 |  |  | 100.2 |  | cilazapril | HCTZ |  | 0.5 | 25 |  | 1 |
|  | A025 | 92 | 53.9 | 12.1 |  |  | 99.4 |  | cilazapril | HCTZ |  | 5 |  |  | 1 |
|  | A025 | 93 | 53.9 | 12.1 |  |  | 100.4 |  | cilazapril | HCTZ |  | 5 | 25 |  | 1 |
|  | A025 | 97 | 53.9 | 12.1 |  |  | 100.5 |  | cilazapril | HCTZ |  | 10 | 12.5 |  | 1 |
|  | A025 | 93 | 53.9 | 12.1 |  |  | 100.7 |  | cilazapril | HCTZ |  | 10 | 25 |  | 1 |
| Wikstrand J. et al . JAMA. 1986 | A026 | 281 | 66.4 | 4.4 | 186 | 18 | 108 | 6 | metoprolol |  |  | 100 |  |  | 1 |
|  | A026 | 281 | 67.2 | 4.7 | 188 | 18 | 108 | 7 | hidroclorotiazida |  |  | 25 |  |  | 1 |
| Cocke TB. Et al J Clin Pharmacol. 1977 | A027 | 46 |  |  | 166 |  | 105 |  | triamtereno | HCTZ |  | 50 | 25 |  | 1 |
|  | A027 | 46 |  |  | 166 |  | 109 |  | espironolactona | HCTZ |  | 25 | 25 |  | 1 |
| Schnaper HW. Et al Gerontology. 1987 | A028 | 93 | 70.4 | 0.5 | 166.7 |  | 93.9 |  | enalapril |  |  | 10 |  |  | 1 |
|  | A028 | 81 | 69.7 | 0.6 | 166.6 |  | 93.6 |  | hidroclorotiazida |  |  | 12.5 |  |  | 1 |
| Ruilope LM. Et al Blood Press. 1996 | A029 | 80 | 52.0 |  | 164 | 18 | 106 | 7 | losartan | PLACEBO |  | 50 |  |  | 1 |
|  | A029 | 80 | 52.3 |  | 163 | 18 | 105 | 7 | losartan | HCTZ |  | 50 | 6.25 |  | 1 |
|  | A029 | 80 | 51.1 |  | 161 | 18 | 105 | 7 | losartan | HCTZ |  | 50 | 12.5 |  | 1 |
|  | A029 | 80 | 52.5 |  | 167 | 17 | 106 | 7 | losartan | HCTZ |  | 50 | 25 |  | 1 |
| Palú CD. Et al Current The Res. 1993 | A030 | 48 |  |  | 178.8 |  | 101.8 |  | lisinopril |  |  | 10 |  |  | 1 |
|  | A030 | 48 |  |  | 174.5 |  | 101.8 |  | hidroclorotiazida |  |  | 12.5 |  |  | 1 |
| Burris JF. Et al Arch Intern Med. 1989 | A031 | 93 | 51 | 1 | 161 | 1.8 | 105 | 0.5 | Betaxolol |  |  | 20 |  |  | 1 |
|  | A031 | 93 | 51 | 1.2 | 161 | 2 | 105 | 0.5 | Clortalidon |  |  | 25 |  |  | 1 |
| Schoenberger JA. Et al . J Hypertens Suppl. 1995 | A032 | 140 | 54 |  | 152.3 | 15 | 101.2 | 5.3 | Placebo |  |  |  |  |  | 1 |
|  | A032 | 142 | 52 |  | 152.2 | 14.4 | 100.9 | 4.9 | HCTZ |  |  | 12.5 |  |  | 1 |
|  | A032 | 139 | 55 |  | 152.2 | 15.2 | 100.9 | 5 | Losartan |  |  | 50 |  |  | 1 |
|  | A032 | 144 | 53 |  | 152.6 | 16.6 | 101.2 | 5.3 | Losartan | HCTZ |  | 50 | 6.25 |  | 1 |
|  | A032 | 138 | 53 |  | 151.3 | 14.2 | 101.7 | 5.5 | Losartan | HCTZ |  | 50 | 12.5 |  | 1 |
| London G. et al Am J Hypertens. 2006 | A033 | 439 | 59 | 10.1 | 164.6 | 8.5 | 96.3 | 8.7 | Placebo |  |  |  |  |  | 1 |
|  | A033 | 440 | 58.4 | 10.3 | 164.4 | 8.4 | 96.5 | 8.4 | Indapamida SR |  |  | 1.5 |  |  | 1 |
|  | A033 | 435 | 59.4 | 10.3 | 164.2 | 8.4 | 96.7 | 7.9 | Candesartan |  |  | 8 |  |  | 1 |
|  | A033 | 444 | 58.9 | 9.9 | 164.6 | 8.4 | 97.1 | 8.5 | Amlodipino |  |  | 5 |  |  | 1 |
| WH Frishman. Journal of Clinical Pharmacology. 1995 | A034 | 150 |  |  | 151.8 | 13 | 100.6 | 4.4 | Placebo |  |  |  |  |  | 1 |
|  | A034 | 75 |  |  | 152 | 13.2 | 100.4 | 3.8 | Bisoprolol |  |  | 5 |  |  | 1 |
|  | A034 | 151 |  |  | 149.9 | 13.3 | 100.2 | 4.2 | HCTZ |  |  | 25 |  |  | 1 |
|  | A034 | 133 |  |  | 150.5 | 13.4 | 100.7 | 4.3 |  |  |  |  |  |  |  |
| L. Widmann. European Journal of Clinical Pharmacology. 1990 | A035 | 59 | 57 |  | 167 |  | 105 |  | Carvedilol |  |  | 25 |  |  | 1 |
|  | A035 | 59 | 55 |  | 168 |  | 105 |  | Atenolol |  |  | 50 |  |  | 1 |
| B.H. Meyer. Journal of Cardiovascular Pharmacology. 1994 | A036 | 68 | 50 |  | 157.1 | 1.62 | 102.1 | 0.66 | Trandolapril |  |  | 2 |  |  | 1 |
|  | A036 | 68 | 51 |  | 160.4 | 1.66 | 103.6 | 0.78 | HCTZ |  |  | 2 |  |  | 1 |
|  | A036 | 69 | 51 |  | 159.5 | 1.42 | 103 | 0.62 | Trandolapril | HCTZ |  | 2 | 25 |  | 1 |
| T. Lenz. European Heart Journal. 1994 | A037 | 120 |  |  | 173.5 |  | 109.5 |  | Quinapril |  |  | 15 |  |  | 1 |
|  | A037 | 124 |  |  | 169.8 |  | 109.7 |  | Quinapril | HCTZ |  | 15 | 18.75 |  | 1 |
|  | A037 | 124 |  |  | 170.9 |  | 109.2 |  | HCTZ |  |  | 18.75 |  |  | 1 |
| David A. Calhoun. Hypertension. 2009 | A038 | 583 |  |  | 169.6 | 14.5 | 106.4 | 5.1 | Amlodipino | Valsartan | HCTZ | 10 | 320 | 25 | 1 |
|  | A038 | 559 |  |  | 169.5 | 13.8 | 106.2 | 5.1 | Valsartan | HCTZ |  | 320 | 25 |  | 1 |
|  | A038 | 568 |  |  | 169.6 | 13.7 | 106.6 | 5.1 | Amlodipino | Valsartan |  | 10 | 320 |  | 1 |
|  | A038 | 561 |  |  | 170.8 | 14.3 | 107.1 | 5.1 | Amlodipino | HCTZ |  | 10 | 25 |  | 1 |
| Christen A. Zuschke. Clinical Therapeutics. 1999 | A039 | 91 | 54 | 11 | 150.1 | 14.1 | 100.2 | 3.9 | Candesartan cilexetil |  |  | 16 |  |  | 1 |
|  | A039 | 94 | 53 | 12 | 151.9 | 13.6 | 99.8 | 4 | Candesartan cilexetil |  |  | 8 |  |  | 2 |
|  | A039 | 92 | 53 | 11 | 152.8 | 15.5 | 99.9 | 3.4 | Placebo |  |  |  |  |  | 1 |
| Flack JM. Et al J Hum Hypertens. 2009 | A040 | 286 | 52.9 |  | 170.4 | 9.6 | 98.5 | 10.9 | Amlodipino | Valsartan |  | 5 | 160 |  | 1 |
|  | A040 | 286 | 53.6 |  | 170.5 | 8.9 | 98.2 | 10.1 | Amlodipino |  |  | 5 |  |  | 1 |
| Morisco. C. 1997 | A041 | 107 | 52.95 | 10.14 | 156.96 | 10.63 | 100.03 | 3.07 | Lecarnidipino |  |  | 10 |  |  | 1 |
|  | A041 | 110 | 52.64 | 10.1 | 156.81 | 10.78 | 99.71 | 3.63 | Atenolol |  |  | 50 |  |  | 1 |
| Bortel V. et al Am J Hypertens 2005 | A042 | 147 | 56 | 9 | 166 | 16 | 103 | 6 | Nevibolol |  |  | 5 |  |  | 1 |
|  | A042 | 151 | 56 | 8 | 165 | 18 | 102 | 5 | Losartan |  |  | 50 |  |  | 1 |
| Czuriga I. et al Cardiovasc Drugs Ther 2003 | A043 | 138 | 50 | 8.4 | 153 | 11.7 | 99 | 3.1 | Nevibolol |  |  | 5 |  |  | 1 |
|  | A043 | 135 | 49 | 8.2 | 153 | 11.5 | 100 | 3.1 | Bisoprolol |  |  | 5 |  |  | 1 |
| Wald DS. Et al Clin Ther. 2008 | A044 | 47 | 62 | 11 | 145 |  | 82 |  | atenolol |  |  | 25 |  |  | 1 |
|  | A044 | 47 | 62 | 11 | 145 |  | 82 |  | lisinopril |  |  | 5 |  |  | 1 |
| Lacourcière Y. et al J Clin Pharmacol. 1992 | A045 | 26 | 51.5 | 10.4 | 153.1 | 19.8 | 99.2 | 3.6 | nebivolol |  |  | 5 |  |  | 1 |
|  | A045 | 25 | 53 | 7.7 | 151.2 | 12.3 | 99.7 | 2.7 | nifedipino |  |  | 20 |  |  | 2 |
| Toal CB. Et al Can J Cardiol. 1997 | A046 | 94 | 55 | 11 | 148 | 7.8 | 98.7 | 3.5 | placebo |  |  | 0 |  |  | 1 |
|  | A046 | 93 | 55 | 11 | 149.1 | 9.3 | 98.4 | 3.3 | nifedipino |  |  | 20 |  |  | 1 |
| Van Bortel L. J Cardiovasc Pharmacol. 1993 | A047 | 32 |  |  | 165 |  | 101 |  | nebivolol |  |  | 5 |  |  | 1 |
| Van Nueten L. et al J Hum Hypertens. 1997 | A048 | 32 |  |  | 165 |  | 101 |  | nebivolol |  |  | 5 |  |  | 1 |
|  | A048 | 83 |  |  | 158.8 |  | 101.3 |  | placebo |  |  | 0 |  |  | 1 |
|  | A048 | 83 |  |  | 157.7 |  | 100.9 |  | nebivolol |  |  | 0.5 |  |  | 1 |
|  | A048 | 87 |  |  | 160.9 |  | 101.8 |  | nebivolol |  |  | 1 |  |  | 1 |
|  | A048 | 84 |  |  | 161.2 |  | 101.8 |  | nebivolol |  |  | 2.5 |  |  | 1 |
|  | A048 | 86 |  |  | 160.3 |  | 101.6 |  | nebivolol |  |  | 5 |  |  | 1 |
|  | A048 | 84 |  |  | 157.9 |  | 101.2 |  | nebivolol |  |  | 10 |  |  | 1 |
| Mazza A. et al Blood Press. 2002 | A049 | 81 | 70.1 | 5.1 | 161 |  | 100.5 |  | nebivolol |  |  | 5 |  |  | 1 |
|  | A049 | 87 | 70.6 | 5.1 | 162 |  | 100 |  | amlodipino |  |  | 10 |  |  | 1 |
| Dahlöf B. et al J Cardiovasc Pharmacol | A050 | 48 | 56 | 9 | 163 | 14 | 103 | 4 | metoprolol | felodipino |  | 100 | 10 |  | 1 |
|  | A050 | 47 | 55 | 8 | 164 | 16 | 102 | 5 | felodipino |  |  | 10 |  |  | 1 |
|  | A050 | 50 | 56 | 8 | 163 | 16 | 103 | 5 | metoprolol |  |  | 100 |  |  | 1 |
| Hoffman J. et al J. Drug. Dev. 1991 | A051 | 62 | 52 | 7 | 176 | 12 | 105 | 5 | metoprolol | felodipino |  | 50 | 5 |  | 1 |
|  | A051 | 62 | 51 | 7 | 172 | 10 | 104 | 4 | felodipino |  |  | 5 |  |  | 1 |
|  | A051 | 62 | 53 | 7 | 170 | 8 | 102 | 4 | metoprolol |  |  | 50 |  |  | 1 |
| Frishman WH. Et al Am J Hypertens. 2006 | A052 | 95 |  |  | 151.4 |  | 99.3 |  | placebo |  |  | 0 |  |  | 1 |
|  | A052 | 88 | 54 |  | 151.6 |  | 99.7 |  | metoprolol |  |  | 25 |  |  | 1 |
|  | A052 | 44 | 54 |  | 151.9 |  | 99.3 |  | metoprolol |  |  | 100 |  |  | 1 |
|  | A052 | 90 | 54 |  | 152.4 |  | 100.3 |  | metoprolol |  |  | 400 |  |  | 1 |
|  | A052 | 93 | 54 |  | 154.2 |  | 100.3 |  | felodipino |  |  | 2.5 |  |  | 1 |
|  | A052 | 46 | 54 |  | 153.7 |  | 100.2 |  | felodipino |  |  | 10 |  |  | 1 |
|  | A052 | 89 | 54 |  | 152.9 |  | 99.7 |  | felodipino |  |  | 20 |  |  | 1 |
|  | A052 | 88 | 54 |  | 154 |  | 99.6 |  | metoprolol | felodipino |  | 25 | 2.5 |  | 1 |
|  | A052 | 45 | 54 |  | 152.3 |  | 99.6 |  | metoprolol | felodipino |  | 25 | 10 |  | 1 |
|  | A052 | 43 | 54 |  | 150.9 |  | 99.7 |  | metoprolol | felodipino |  | 25 | 20 |  | 1 |
|  | A052 | 51 | 54 |  | 152 |  | 99.1 |  | metoprolol | felodipino |  | 100 | 2.5 |  | 1 |
|  | A052 | 93 | 54 |  | 152.5 |  | 100.3 |  | metoprolol | felodipino |  | 100 | 10 |  | 1 |
|  | A052 | 47 | 54 |  | 153.2 |  | 100.8 |  | metoprolol | felodipino |  | 100 | 20 |  | 1 |
|  | A052 | 46 | 54 |  | 149.8 |  | 99.9 |  | metoprolol | felodipino |  | 400 | 2.5 |  | 1 |
|  | A052 | 45 | 54 |  | 155.1 |  | 100.2 |  | metoprolol | felodipino |  | 400 | 10 |  | 1 |
|  | A052 | 84 | 54 |  | 152.8 |  | 100.2 |  | metoprolol | felodipino |  | 400 | 20 |  | 1 |
| Maclean D. et al Br J Clin Pharmacol. 1990 | A053 | 32 | 48.8 | 11.1 | 171 |  | 108 |  | placebo |  |  | 0 |  |  | 1 |
|  | A053 | 37 | 49.8 | 11.2 | 167 |  | 107 |  | atenolol |  |  | 50 |  |  | 1 |
| Rosei EA. Et al Blood Press Suppl. 2003 | A054 | 36 | 50.1 | 8.2 | 159.3 | 13.7 | 101 | 5.1 | nebivolol |  |  | 5 |  |  | 1 |
|  | A054 | 32 | 48.3 | 9.6 | 156.4 | 14.5 | 98.6 | 3.1 | lisinopril |  |  | 20 |  |  | 1 |
| Oparil S. et al Current The Res. 1980 | A055 | 34 | 54 |  | 163 |  | 103 |  | timolol | HCTZ |  | 10 | 25 |  | 2 |
|  | A055 | 33 | 48.8 |  | 160 |  | 105 |  | timolol |  |  | 10 |  |  | 2 |
| Dietz R. et al . J Renin Angiotensin Aldosterone Syst. 2008 | A056 | 231 | 55.8 | 11.9 | 157.6 | 12.3 | 99.7 | 3.8 | Aliskiren |  |  | 225 |  |  | 1 |
|  | A056 | 231 | 54.7 | 11.5 | 155.9 | 12.9 | 99.4 | 3.8 | Atenolol |  |  | 75 |  |  | 1 |
|  | A056 | 232 | 55.2 | 10.9 | 157.3 | 12.3 | 99.5 | 3.8 | Aliskiren | Atenolol |  | 225 | 75 |  | 1 |
| Uhlíř O. et al Drug Invest. 1991 | A057 | 67 |  |  | 157 |  | 107 |  | metoprolol |  |  | 100 |  |  | 2 |
|  | A057 | 73 |  |  | 160 |  | 106 |  | nebivolol |  |  | 5 |  |  | 1 |
| Klein G. et al J Clin Pharmacol. 1990 | A058 | 96 | 49.9 | 8.7 | 166 | 13 | 102 | 4 | atenolol |  |  | 50 |  |  | 1 |
|  | A058 | 95 | 49.9 | 8.7 | 166 | 13 | 102 | 4 | atenolol |  |  | 100 |  |  | 1 |
| Havinga TK. Et al Neth J Med. 1991 | A059 | 62 | 51 | 8 | 170 | 19 | 107 | 8 | atenolol |  |  | 50 |  |  | 1 |
|  | A059 | 63 | 53 | 9 | 170 | 20 | 107 | 7 | captopril |  |  | 25 |  |  | 2 |
| Dahlöf B. et al Blood Press Suppl. 1993 | A060 | 54 | 56 | 9 | 163 | 14 | 103 | 4 | felodipino | metoprolol |  | 10 | 100 |  | 1 |
|  | A060 | 53 | 55 | 8 | 164 | 16 | 102 | 5 | felodipino |  |  | 10 |  |  | 1 |
|  | A060 | 52 | 56 | 8 | 163 | 16 | 103 | 5 | metoprolol |  |  | 100 |  |  | 1 |
| Mallion JM. Et al Clin Exp Hypertens. 2000 | A061 | 222 | 56.1 | 11.6 | 163.2 | 10.5 | 100.9 | 4.3 | perindopril | indapamida |  | 2 | 0.625 |  | 1 |
|  | A061 | 224 | 55.5 | 10.4 | 163.4 | 11.1 | 100.4 | 3.8 | atenolol |  |  | 50 |  |  | 1 |
| Parrinello G. et al Clin Drugs Invest. 2009 | A062 | 36 | 51 | 12 | 154 | 16.5 | 92.1 | 11.6 | losartan |  |  | 50 |  |  | 1 |
|  | A062 | 36 | 49 | 11 | 156 | 15.5 | 94.7 | 10.6 | bisoprolol |  |  | 5 |  |  | 1 |
| Ravid M. et al Arch Intern Med. 1985 | A063 | 66 |  |  | 172.3 |  | 109.2 |  | atenolol |  |  | 100 |  |  | 1 |
|  | A063 | 66 |  |  | 172.3 |  | 109.2 |  | placebo |  |  | 0 |  |  | 1 |
|  | A063 | 68 |  |  | 170 |  | 109.3 |  | atenolol |  |  | 100 |  |  | 1 |
|  | A063 | 68 |  |  | 170 |  | 109.3 |  | placebo |  |  | 0 |  |  | 1 |
| Suzanne Oparil. Clinical Therapeutics. 2010 | A064 | 628 |  |  | 168.1 | 13.5 | 100.9 | 7.8 | Olmesartan | Amlodipino |  | 40 | 10 |  | 1 |
|  | A064 | 637 |  |  | 169 | 14.9 | 100.7 | 8.2 | Olmesartan | HCTZ |  | 40 | 25 |  | 1 |
|  | A064 | 600 |  |  | 168.9 | 14.5 | 101.3 | 7.6 | Amlodipino | HCTZ |  | 10 | 25 |  | 1 |
| Finnerty FA Jr. Et al Angiology. 1976 | A065 | 26 | 42.0 |  | 152.3 |  | 104.1 |  | clortalidona |  |  | 50 |  |  | 2 |
|  | A065 | 28 | 41.0 |  | 153.1 |  | 104.5 |  | hidroclorotiazida |  |  | 100 |  |  | 2 |
| Gradman AH. Et al Clin Ther. 2002 | A066 | 173 | 52.3 | 9.88 | 159.9 | 13.59 | 107.9 | 2.78 | losartan | HCTZ |  | 100 | 25 |  | 1 |
|  | A066 | 184 | 53.2 | 9.27 | 160.4 | 13.76 | 108.0 | 2.76 | losartan | HCTZ |  | 100 | 12.5 |  | 1 |
|  | A066 | 89 | 52.8 | 9.42 | 161.0 | 13.82 | 108.1 | 3.27 | placebo |  |  |  |  |  | 1 |
| Mengden T. et al Eur J Clin Pharmacol. 1992 | A067 | 27 |  |  | 155 | 17 | 102 | 5 | nitrendipino |  |  | 20 |  |  | 1 |
|  | A067 | 26 |  |  | 150 | 15 | 100 | 5 | bisoprolol |  |  | 10 |  |  | 1 |
| Nugent LW. Et al J Clin Pharmacol. 1987 | A068 | 62 | 49 |  | 159.6 |  | 101.5 |  | enalapril |  |  | 5 |  |  | 1 |
|  | A068 | 67 | 53 |  | 158.3 |  | 101.2 |  | metoprolol |  |  | 50 |  |  | 1 |
| Talseth T. et al Am Heart J. 1988 | A069 | 110 | 51.2 |  | 159 |  | 104 |  | Doxazosina |  |  | 6.3 |  |  | 1 |
|  | A069 | 118 | 52.6 |  | 161 |  | 104 |  | Atenolol |  |  | 66.5 |  |  | 1 |
| Davidov ME. Et al Clin Cardiol. 1994 | A070 | 69 | 53 | 11 | 151.9 | 1.8 | 99.4 | 0.6 | Placebo |  |  |  |  |  | 1 |
|  | A070 | 67 | 53 | 11 | 149.4 | 2.1 | 100.2 | 0.6 | Bisoprolol |  |  | 5 |  |  | 1 |
|  | A070 | 69 | 53 | 10 | 146.6 | 2 | 100 | 0.6 | Bisoprolol |  |  | 10 |  |  | 1 |
|  | A070 | 71 | 54 | 11 | 149.4 | 2.1 | 99.8 | 0.6 | Bisoprolol |  |  | 20 |  |  | 1 |
| Mark Greathouse. Clinical Investigations. 2010 | A071 | 75 | 51.2 | 10 | 149.9 | 12.5 | 98.7 | 3.3 | Placebo |  |  |  |  |  | 1 |
|  | A071 | 244 | 53.9 | 11.1 | 151.8 | 13.2 | 99.1 | 3.8 | Nebivolol |  |  | 5 |  |  | 1 |
|  | A071 | 244 | 53.8 | 11.2 | 150.5 | 13.1 | 98.9 | 4.4 | Nebivolol |  |  | 10 |  |  | 1 |
|  | A071 | 244 | 53.4 | 11.1 | 151.9 | 14.8 | 99.2 | 3.7 | Nebivolol |  |  | 20 |  |  | 1 |
| N.K. Gostick. Current Medical Research and Opinion | A072 | 84 | 50.1 |  | 165.6 | 2 | 101.9 | 0.75 | Placebo |  |  |  |  |  | 1 |
|  | A072 | 84 | 50.1 |  | 165.6 | 2 | 101.9 | 0.75 | Atenolol |  |  | 50 |  |  | 1 |
|  | A072 | 84 | 50.1 |  | 165.6 | 2 | 101.9 | 0.75 | Atenolol |  |  | 100 |  |  | 1 |
|  | A072 | 84 | 50.1 |  | 165.6 | 2 | 101.9 | 0.75 | Atenolol |  |  | 200 |  |  | 1 |
| Dahlöf B. et al Am J Hypertens. 1995 | A073 | 134 | 52.4 |  | 156.9 | 19.8 | 103.1 | 5.8 | Losartan |  |  | 65.75 |  |  | 1 |
|  | A073 | 68 | 53.9 |  | 160.2 | 16.7 | 103.6 | 6.1 | Atenolol |  |  | 64.75 |  |  | 1 |
| Letellier P. et al J Hum Hypertens. 1994 | A074 | 57 | 53.8 |  | 160 |  | 101 |  | perindopril | nifedipino |  | 8 | 20 |  | 1 |
|  | A074 | 53 | 56.8 |  | 161 |  | 101 |  | perindopril | HCTZ |  | 8 | 12.5 |  | 1 |
| Van Neuten L. et al Am J Ther. 1998 | A075 | 211 | 54 |  | 161 |  | 104.2 |  | nebivolol |  |  | 5 |  |  | 1 |
|  | A075 | 209 | 53 |  | 160 |  | 104.5 |  | nifedipino |  |  | 20 |  |  | 2 |
| Strocchi E. et al Clin. Ther. 1991 | A076 | 81 | 52.4 | 9.2 | 166.5 | 17.1 | 108.5 | 11.1 | enalapril |  |  | 10 |  |  | 1 |
|  | A076 | 81 | 52.4 | 9.2 | 166.5 | 17.1 | 108.5 | 11.1 | enalapril |  |  | 20 |  |  | 1 |
|  | A076 | 81 | 52.4 | 9.2 | 166.5 | 17.1 | 108.5 | 11.1 | enalapril | HCTZ |  | 20 | 12.5 |  | 1 |
|  | A076 | 81 | 52.4 | 9.2 | 166.5 | 17.1 | 108.5 | 11.1 | enalapril | HCTZ |  | 40 | 25 |  | 1 |
| Klein G. et al Blood Press. 1998 | A077 | 54 | 57 | 9.8 | 163.9 | 13.9 | 102.3 | 5.4 | felodipino | metoprolol |  | 10 | 100 |  | 1 |
|  | A077 | 55 | 55.1 | 10.8 | 163.6 | 13.4 | 102.5 | 5.7 | captopril | HCTZ |  | 50 | 25 |  | 1 |
| P.K. Zachariah. Journal of Cardiovascular Pharmacology. 1987 | A078 | 118 | 53 |  | 168 |  | 111 |  | Lisinopril |  |  | 40 |  |  | 1 |
|  | A078 | 61 | 52.6 |  | 168 |  | 110 |  | Metoprolol |  |  | 100 |  |  | 1 |
| Elijah Saunders. Arch Intern Med. 1990 | A079 | 133 |  |  | 152 | 13.6 | 100.9 | 5.9 |  |  |  |  |  |  |  |
|  | A079 | 131 |  |  | 150.2 | 15.4 | 100.4 | 5.5 |  |  |  |  |  |  |  |
|  | A079 | 130 |  |  | 151 | 14.8 | 100.8 | 4.6 |  |  |  |  |  |  |  |
| Policicchio D. et al J. Cardiovasc. Pharm 1997 | A080 | 64 | 57.1 | 9 | 162.57 | 12.29 | 100.99 | 4.62 | Lecarnidipino |  |  | 10 |  |  | 1 |
|  | A080 | 66 | 58.4 | 7 | 163.03 | 13.52 | 101.08 | 4.46 | Nifedipino |  |  | 20 |  |  | 2 |
| Romito R. et al J Clin Hypertens 2003 | A081 | 89 | 57.5 | 9.3 | 154.7 | 11.4 | 98.7 | 3.4 | Lecarnidipino |  |  | 10 |  |  | 1 |
|  | A081 | 79 | 55.6 | 8.3 | 155.1 | 11.5 | 98.7 | 3 | Felodipino |  |  | 10 |  |  | 1 |
|  | A081 | 82 | 58.4 | 8.6 | 155.1 | 12.1 | 98.6 | 3.2 | Nifedipino |  |  | 30 |  |  | 1 |
| Kloner RA. Et al Am J Cardiol. 2001 | A082 | 123 | 53 | 11 | 144.9 | 14.5 | 94.2 | 2.9 | Candesartan Cilexetil |  |  | 24 |  |  | 1 |
|  | A082 | 128 | 54 | 13 | 145.8 | 13.5 | 94.1 | 3 | Amlodipino |  |  | 7.5 |  |  | 1 |
| Ambrosioni E. et al J Hypertens. 1998 | A083 | 58 | 53 | 8 | 164.4 | 13.5 | 102.5 | 5.3 | Placebo |  |  |  |  |  | 1 |
|  | A083 | 59 | 55 | 10 | 164.4 | 16.2 | 101.2 | 4.6 | Indapamida |  |  | 2.5 |  |  | 1 |
|  | A083 | 57 | 55 | 11 | 161 | 16.3 | 101 | 4.4 | Indapamida SR |  |  | 1.5 |  |  | 1 |
|  | A083 | 55 | 54 | 11 | 164.5 | 15 | 101.7 | 5.5 | Indapamida SR |  |  | 2 |  |  | 1 |
|  | A083 | 56 | 53 | 10 | 161.8 | 16.7 | 101.5 | 5 | Indapamida SR |  |  | 2.5 |  |  | 1 |
|  | A083 | 200 | 53 | 10 | 161.7 | 16 | 100.6 | 4 | Indapamida SR |  |  | 1.5 |  |  | 1 |
|  | A083 | 205 | 57 | 10 | 164.4 | 15.7 | 101.5 | 4.7 | Indapamida |  |  | 2.5 |  |  | 1 |
| Philipp T. et al . Clin Ther. 2007 | A084 | 128 | 53.7 |  | 151.6 | 12.57 | 99.4 | 3.72 | Placebo |  |  |  |  |  | 1 |
|  | A084 | 127 | 55.3 |  | 152.5 | 12.25 | 99.3 | 3.75 | Amlodipino | Valsartan |  | 5 | 320 |  | 1 |
|  | A084 | 127 | 54.9 |  | 153 | 13.07 | 99.4 | 3.59 | Amlodipino | Valsartan |  | 5 | 160 |  | 1 |
|  | A084 | 128 | 54.2 |  | 153.2 | 12.75 | 99.1 | 3.32 | Amlodipino | Valsartan |  | 5 | 80 |  | 1 |
|  | A084 | 125 | 53.4 |  | 153 | 13.68 | 99.4 | 3.48 | Amlodipino | Valsartan |  | 5 | 40 |  | 1 |
|  | A084 | 129 | 55.1 |  | 152.3 | 12.49 | 99.4 | 3.71 | Amlodipino | Valsartan |  | 2.5 | 320 |  | 1 |
|  | A084 | 127 | 55.1 |  | 152.1 | 13.29 | 99 | 3.42 | Amlodipino | Valsartan |  | 2.5 | 160 |  | 1 |
|  | A084 | 130 | 54.1 |  | 151.8 | 13.71 | 99.5 | 3.88 | Amlodipino | Valsartan |  | 2.5 | 80 |  | 1 |
|  | A084 | 129 | 54.5 |  | 153.1 | 13.21 | 99.6 | 3.84 | Amlodipino | Valsartan |  | 2.5 | 40 |  | 1 |
|  | A084 | 128 | 53.8 |  | 152.6 | 12.7 | 99 | 3.49 | Amlodipino |  |  | 5 |  |  | 1 |
|  | A084 | 126 | 54.4 |  | 153.9 | 12.86 | 99.5 | 3.73 | Amlodipino |  |  | 2.5 |  |  | 1 |
|  | A084 | 128 | 56.8 |  | 154.6 | 11.41 | 99.3 | 3.59 | Valsartan |  |  | 320 |  |  | 1 |
|  | A084 | 128 | 53 |  | 152 | 14.19 | 98.9 | 3.54 | Valsartan |  |  | 160 |  |  | 1 |
|  | A084 | 124 | 53.1 |  | 153.2 | 11.63 | 99.2 | 3.55 | Valsartan |  |  | 80 |  |  | 1 |
|  | A084 | 127 | 55 |  | 153.7 | 12.56 | 99.2 | 3.22 | Valsartan |  |  | 40 |  |  | 1 |
|  | A084 | 209 | 58 |  | 156.4 | 11.5 | 99 | 3.3 | Placebo |  |  |  |  |  | 1 |
|  | A084 | 210 | 58 |  | 157.2 | 12 | 99.2 | 3.6 | Amlodipino | Valsartan |  | 10 | 320 |  | 1 |
|  | A084 | 209 | 56.7 |  | 157.4 | 11.5 | 99.3 | 3.6 | Amlodipino | Valsartan |  | 10 | 160 |  | 1 |
|  | A084 | 207 | 55.4 |  | 156.2 | 12.6 | 98.8 | 3.2 | Amlodipino |  |  | 10 |  |  | 1 |
|  | A084 | 208 | 56.7 |  | 157.5 | 11.5 | 99.1 | 3.6 | Valsartan |  |  | 320 |  |  | 1 |
|  | A084 | 207 | 56.8 |  | 155.6 | 11.3 | 98.9 | 3.3 | Valsartan |  |  | 160 |  |  | 1 |
| Malacco E. et al Int J Clin Pharmacol Ther. 2002 | A085 | 201 | 56 | 10 | 164.9 | 14.6 | 101.9 | 7.4 | benazepril | amlodipino |  | 10 | 5 |  | 1 |
|  | A085 | 196 | 57 | 11 | 163.2 | 15.3 | 101.4 | 7.6 | captopril | HCTZ |  | 50 | 25 |  | 1 |
| Chrysant SG. Et al Clin Ther. 2008 | A086 | 161 | 53.8 | 11 | 162.9 | 16.7 | 101.8 | 5.9 | olmesartan |  |  | 10 |  |  | 1 |
|  | A086 | 161 | 53.6 | 10.8 | 164.1 | 16.5 | 101.5 | 4.6 | olmesartan |  |  | 20 |  |  | 1 |
|  | A086 | 162 | 53.9 | 10.6 | 162.8 | 15.7 | 101.2 | 5.1 | olmesartan |  |  | 40 |  |  | 1 |
|  | A086 | 161 | 53.4 | 11.4 | 162.6 | 17.2 | 101.5 | 5.2 | amlodipino |  |  | 5 |  |  | 1 |
|  | A086 | 163 | 54.1 | 10.6 | 163.5 | 15.9 | 101.6 | 4.8 | amlodipino |  |  | 10 |  |  | 1 |
|  | A086 | 163 | 53.7 | 11.8 | 165.5 | 15.6 | 102.1 | 5.4 | olmesartan | amlodipino |  | 10 | 5 |  | 1 |
|  | A086 | 161 | 54.3 | 10.9 | 163.8 | 14.9 | 101.7 | 5.1 | olmesartan | amlodipino |  | 20 | 5 |  | 1 |
|  | A086 | 162 | 53.7 | 11.3 | 161.7 | 14.8 | 100.9 | 4.8 | olmesartan | amlodipino |  | 40 | 5 |  | 1 |
|  | A086 | 162 | 54.3 | 10.5 | 162.5 | 15.6 | 101.4 | 5.5 | olmesartan | amlodipino |  | 10 | 10 |  | 1 |
|  | A086 | 160 | 54.3 | 11.6 | 164.1 | 14.9 | 101.2 | 4.7 | olmesartan | amlodipino |  | 20 | 10 |  | 1 |
|  | A086 | 162 | 54.1 | 12.2 | 165.7 | 16.7 | 102.4 | 5.8 | olmesartan | amlodipino |  | 40 | 0 |  | 1 |
|  | A086 | 162 | 54.7 | 10.9 | 166.5 | 17.6 | 102.3 | 4.8 | placebo |  |  | 0 |  |  | 1 |
| DeQuattro V. et al Am J Hypertens. 1997 | A087 | 54 |  |  | 154 |  | 100 |  | placebo |  |  | 0 |  |  | 1 |
|  | A087 | 35 |  |  | 148 |  | 100 |  | verapamil |  |  | 120 |  |  | 1 |
|  | A087 | 57 |  |  | 153 |  | 99 |  | verapamil |  |  | 180 |  |  | 1 |
|  | A087 | 48 |  |  | 150 |  | 101 |  | verapamil |  |  | 240 |  |  | 1 |
| Kuschnir E. et al Clin Ther. 1996 | A088 | 77 | 56.8 | 9.6 | 167.7 |  | 106.3 |  | amlodipino | benazepril |  | 5 | 20 |  | 1 |
|  | A088 | 77 | 56.2 | 9.4 | 165.6 |  | 106.5 |  | amlodipino |  |  | 5 |  |  | 1 |
|  | A088 | 77 | 57.2 | 9.5 | 166.4 |  | 106.9 |  | placebo |  |  | 0 |  |  | 1 |
| Levine JH. Et al Am J Hypertens. 1995 | A089 | 30 | 52 |  | 150.4 |  | 101.2 |  | verapamil |  |  | 120 |  |  | 1 |
|  | A089 | 31 | 56 |  | 152.5 |  | 102.5 |  | enalapril |  |  | 10 |  |  | 1 |
|  | A089 | 32 | 53 |  | 151 |  | 100.4 |  | verapamil |  |  | 240 |  |  | 1 |
|  | A089 | 29 | 53 |  | 149.8 |  | 100.2 |  | placebo |  |  | 0 |  |  | 1 |
| Messerli F. et al Am J Hypertens. 1998 | A090 | 152 | 53.8 |  | 153.6 | 13.4 | 100.5 | 4.5 | placebo |  |  | 0 |  |  | 1 |
|  | A090 | 159 | 54.3 |  | 151.8 | 14.8 | 101.3 | 5 | trandolapril |  |  | 4 |  |  | 1 |
|  | A090 | 157 | 53.8 |  | 151.1 | 14.6 | 100.8 | 4.7 | verapamil |  |  | 240 |  |  | 1 |
| Mancia G. J Hypertens. 2011 | A091 | 164 | 58.3 | 10.4 | 151.1 | 11.8 | 90.9 | 9.4 | nifedipino | Telmisartan |  | 20 | 80 |  | 1 |
|  | A091 | 89 | 59.4 | 10.4 | 151.3 | 11.9 | 90.7 | 7.7 | nifedipino |  |  | 20 |  |  | 1 |
|  | A091 | 74 | 59.3 | 9.4 | 151.7 | 11.8 | 92 | 9 | telmisartan |  |  | 80 |  |  | 1 |
| Lombardo D. et al J Cardiovasc Pharmacol. 1994 | A092 | 40 | 54.9 |  | 177 |  | 104 |  | lacidipino |  |  | 4 |  |  | 1 |
|  | A092 | 40 | 53 |  | 179 |  | 104 |  | amlodipino |  |  | 10 |  |  | 1 |
| Predel H-G. et al J Hum Hypertens. 1994 | A093 | 145 |  |  | 171 |  | 104 |  | lisinopril |  |  | 20 |  |  | 1 |
|  | A093 | 145 |  |  | 170 |  | 104 |  | nifedipino |  |  | 20 |  |  | 2 |
| Nicaise J. et al J Int Med Res. 1995 | A094 | 50 |  |  | 177 | 2 | 102 | 1 | diltiazem |  |  | 200 |  |  | 1 |
|  | A094 | 48 |  |  | 178 | 2 | 103 | 1 | captopril |  |  | 12.5 |  |  | 1 |
| Chan JC. Et al J Hum Hypertens. 1995 | A095 | 89 | 70.4 | 4.5 |  |  | 102.0 | 5.6 | losartan |  |  | 50 |  |  | 1 |
|  | A095 | 43 | 69.9 | 5.3 |  |  | 102.5 | 5.6 | felodipino |  |  | 5 |  |  | 1 |
| Leon AS. Et al Clin Ther. 1993 | A096 | 117 | 55 | 12 | 153 | 17 | 99 | 4 | enalapril |  |  | 5 |  |  | 1 |
|  | A096 | 113 | 55 | 12 | 157 | 17 | 99 | 5 | nifedipino |  |  | 30 |  |  | 1 |
| Pool PE. Et al Pharmacotherapy. 1993 | A097 | 57 | 53.5 | 10.5 | 152.9 | 1.9 | 99.9 | 0.5 | Placebo |  |  |  |  |  | 2 |
|  | A097 | 67 | 56.1 | 10.7 | 156.1 | 1.4 | 99.7 | 0.4 | Diltiazem SR | HCTZ |  | 120 | 12.5 |  | 2 |
|  | A097 | 63 | 55.4 | 9.2 | 152.7 | 1.6 | 99.4 | 0.3 | Diltiazem SR |  |  | 120 |  |  | 2 |
|  | A097 | 67 | 52.7 | 10.6 | 153.4 | 2.2 | 100.2 | 0.5 | HCTZ |  |  | 12.5 |  |  | 2 |
| Ferdinand KC. Et al J Clin Hypertens. 2011 | A098 | 166 | 52 | 11.3 | 168.2 | 8.5 | 96 | 9.8 | Aliskiren | HCTZ |  | 281.25 | 23.44 |  | 1 |
|  | A098 | 166 | 53.1 | 11.6 | 168.1 | 8 | 95 | 8.9 | Amlodipino |  |  | 9.38 |  |  | 1 |
| Lederle RM. ET AL J Cardiovasc Pharmacol. 1991 | A099 | 68 | 54.8 | 10 | 161.4 | 13 | 99.6 | 3 | Placebo |  |  |  |  |  | 1 |
|  | A099 | 73 | 54.9 | 11 | 161.3 | 13 | 99.6 | 3 | Nitrendipino |  |  | 10 |  |  | 1 |
| Corea L. et al Clin Pharmacol Ther. 1996 | A100 | 84 | 52.8 | 10.5 | 157.5 | 13.3 | 101.1 | 3.7 | Valsartan |  |  | 80 |  |  | 1 |
|  | A100 | 84 | 52.9 | 8.9 | 161.8 | 12.8 | 101.8 | 4.5 | Amlodipino |  |  | 5 |  |  | 1 |
| Cherubini A. et al Arch Gerontol Geriatr. 2003 | A101 | 84 | 74 | 8 | 166.6 | 11.2 | 97.6 | 4.6 | Lecarnidipino |  |  | 51.64 |  |  | 1 |
|  | A101 | 93 | 74 | 7 | 167.8 | 11.6 | 97.7 | 4.4 | Lacidipino |  |  | 2.35 |  |  | 1 |
|  | A101 | 84 | 72 | 6 | 167.3 | 11.1 | 97.2 | 4.2 | Nifedipino GITS |  |  | 33.03 |  |  | 1 |
| Chrysant SG. Et al J Hum Hypertens. 2003 | A102 | 66 | 52 |  | 154.2 |  | 103.3 |  | Placebo |  |  |  |  |  | 1 |
|  | A102 | 188 | 51.7 |  | 154.9 |  | 104 |  | Olmesartan medoxomil |  |  | 20 |  |  | 1 |
|  | A102 | 186 | 51.1 |  | 155.1 |  | 103.8 |  | Amlodipino |  |  | 5 |  |  | 1 |
| Witchitz S. et al J Hum Hypertens. 1989 | A103 | 137 | 54 | 11 | 167 | 16 | 102 | 5 | Lisinopril |  |  | 20 |  |  | 1 |
|  | A103 | 137 | 53 | 10 | 165 | 15 | 102 | 5 | Nifedipino SR |  |  | 20 |  |  | 2 |
| Ove K Andersson. Journal of Human Hypertension. 1999 | A104 | 58 | 56 | 9 | 166 | 16 | 100 | 6 | Felodipino | Metoprolol |  | 5 | 50 |  | 1 |
|  | A104 | 59 | 55 | 11 | 163 | 15 | 101 | 5 | Enalapril |  |  | 10 |  |  | 1 |
| M.Heikki Frick. Journal of Internal Medicine. 1989 | A105 | 55 | 48 |  | 159.1 |  | 101.7 |  | Amlodipino |  |  | 1.25 |  |  | 1 |
|  | A105 | 50 | 50.3 |  | 159.8 |  | 101.7 |  | Amlodipino |  |  | 2.5 |  |  | 1 |
|  | A105 | 49 | 50.5 |  | 165.4 |  | 103 |  | Amlodipino |  |  | 5 |  |  | 1 |
|  | A105 | 51 | 52.2 |  | 159.6 |  | 101.1 |  | Placebo |  |  |  |  |  | 1 |
| Alberto Zachetti. Journal of Cardiovascular Pharmacology. 2001 | A106 | 245 | 52.4 | 8.7 | 158.2 | 13.5 | 101.8 | 5.3 | Manidipino |  |  | 10 |  |  | 1 |
|  | A106 | 244 | 54 | 9.2 | 158.1 | 12.9 | 100.9 | 5 | Amlodipino |  |  | 5 |  |  | 1 |
| Suzanne Oparil. Clinical Therapeutics. 1996 | A107 | 97 | 55 | 13 | 153.7 | 15.5 | 101.1 | 5.3 | Losartan |  |  | 50 |  |  | 1 |
|  | A107 | 93 | 54 | 12 | 154.9 | 16.7 | 101 | 5.1 | Amlodipino |  |  | 5 |  |  | 1 |
| Enrico Agabiti Rosei. Blood Pressure. 2005 | A108 | 134 | 54 |  | 151 | 11 | 97 | 6 | Nifedipino GITS |  |  | 20 |  |  | 1 |
|  | A108 | 133 | 53 |  | 151 | 11 | 96 | 6 | Enalapril |  |  | 20 |  |  | 1 |
| Joel M Neutel. Journal of the Renin-Angiotensin-Aldosterone System. 2005 | A109 | 89 | 50.9 | 8.6 | 150.7 | 12.4 | 99.7 | 3.5 | Irbesartan |  |  | 150 |  |  | 1 |
|  | A109 | 92 | 51 | 9 | 149.6 | 12.8 | 99.8 | 3.7 | Amlodipino |  |  | 5 |  |  | 1 |
| P.U. Feig. Clinical Therapeutics. 1993 | A110 | 61 | 55 |  | 153 |  | 99 |  | Placebo |  |  |  |  |  | 1 |
|  | A110 | 60 | 55 |  | 151 |  | 99 |  | Nifedipino coat-core |  |  | 30 |  |  | 1 |
|  | A110 | 57 | 54 |  | 152 |  | 100 |  | Nifedipino coat-core |  |  | 60 |  |  | 1 |
|  | A110 | 55 | 57 |  | 155 |  | 100 |  | Nifedipino coat-core |  |  | 90 |  |  | 1 |
| Richard F.Wright. The Journal of Clinical Hypertension. 2011 | A111 | 241 | 55 | 9.83 | 166.8 | 7.03 | 98.6 | 7.71 | Valsartan | Amlodipino |  | 160 | 5 |  | 1 |
|  | A111 | 247 | 57.1 | 9.69 | 168.5 | 9.03 | 97.6 | 7.88 | Losartan |  |  | 100 |  |  | 1 |
|  | A111 | 247 | 57.1 | 9.69 | 168.5 | 9.03 | 97.6 | 7.88 | Losartan | HCTZ |  | 100 | 25 |  | 1 |
| P. Gueret. Drugs. 1990 | A112 | 68 | 55.9 |  | 169.7 |  | 103 |  | Enalapril |  |  | 20 |  |  | 1 |
|  | A112 | 68 | 58 |  | 168.9 |  | 102.6 |  | Nifedipino |  |  | 20 |  |  | 2 |
| Castaigne A. et al Clin Exp Hypertens. 1999 | A113 | 190 | 71.8 | 5.6 | 171.6 | 10.7 | 95.9 | 8.8 | placebo |  |  | 0 |  |  | 1 |
|  | A113 | 193 | 72.9 | 5.3 | 171.1 | 11.4 | 95.1 | 7.7 | perindopril | indapamida |  | 2 | 0.625 |  | 1 |
| Rump LC. Et al J Hum Hypertens. 2006 | A114 | 315 |  |  | 169.7 | 8.7 | 104.7 | 3.8 | olmesartan | HCTZ |  | 20 | 12.5 |  | 1 |
|  | A114 | 314 |  |  | 169.3 | 8.4 | 104.4 | 3.4 | losartan | HCTZ |  | 50 | 12.5 |  | 1 |
| Villamil A. et al J Hypertens. 2007 | A115 | 195 | 54.4 |  | 152.7 |  | 99.3 |  | Placebo |  |  |  |  |  | 1 |
|  | A115 | 184 | 55 |  | 153.2 |  | 99.4 |  | Aliskiren |  |  | 75 |  |  | 1 |
|  | A115 | 185 | 53.5 |  | 153.4 |  | 98.8 |  | Aliskiren |  |  | 150 |  |  | 1 |
|  | A115 | 183 | 54.2 |  | 154.4 |  | 99.3 |  | Aliskiren |  |  | 300 |  |  | 1 |
|  | A115 | 194 | 55.2 |  | 153.4 |  | 99.3 |  | HCTZ |  |  | 6.25 |  |  | 1 |
|  | A115 | 188 | 55.4 |  | 153.4 |  | 99.1 |  | HCTZ |  |  | 12.5 |  |  | 1 |
|  | A115 | 176 | 55.1 |  | 154.5 |  | 99.1 |  | HCTZ |  |  | 25 |  |  | 1 |
|  | A115 | 188 | 55.1 |  | 154.5 |  | 98.9 |  | Aliskiren | HCTZ |  | 75 | 6.25 |  | 1 |
|  | A115 | 193 | 54.4 |  | 154 |  | 100 |  | Aliskiren | HCTZ |  | 75 | 12.5 |  | 1 |
|  | A115 | 186 | 54.7 |  | 152.9 |  | 99 |  | Aliskiren | HCTZ |  | 75 | 25 |  | 1 |
|  | A115 | 176 | 53.9 |  | 153.3 |  | 99 |  | Aliskiren | HCTZ |  | 150 | 6.25 |  | 1 |
|  | A115 | 186 | 54.7 |  | 154.1 |  | 99.1 |  | Aliskiren | HCTZ |  | 150 | 12.5 |  | 1 |
|  | A115 | 188 | 53.7 |  | 153.2 |  | 98.4 |  | Aliskiren | HCTZ |  | 150 | 25 |  | 1 |
|  | A115 | 181 | 55.5 |  | 153.2 |  | 99.5 |  | Aliskiren | HCTZ |  | 300 | 12.5 |  | 1 |
|  | A115 | 173 | 54.8 |  | 154.6 |  | 99.3 |  | Aliskiren | HCTZ |  | 300 | 25 |  | 1 |
| Waeber B. et al Am J Hypertens. 1999 | A116 | 304 | 51 | 10.7 | 157.2 | 15.3 | 101 | 4.4 | placebo |  |  | 0 |  |  | 1 |
|  | A116 | 321 | 52.4 | 10.2 | 158 | 15.4 | 100.9 | 4.6 | enalapril |  |  | 10 |  |  | 1 |
| Guitard C. et al Blood Press Suppl. 1994 | A117 | 64 | 57 | 11 | 167 | 11 | 105 | 3 | placebo |  |  | 0 |  |  | 1 |
|  | A117 | 66 | 58 | 11 | 171 | 12 | 106 | 4 | espirapril |  |  | 6 |  |  | 1 |
|  | A117 | 64 | 58 | 9 | 168 | 14 | 105 | 4 | espirapril |  |  | 12 |  |  | 1 |
|  | A117 | 66 | 58 | 11 | 170 | 12 | 106 | 4 | espirapril |  |  | 24 |  |  | 1 |
| Vreugdenhil G. et al Blood Press Suppl. 1994 | A118 | 25 |  |  | 168 |  | 106 |  | espirapril |  |  | 3 |  |  | 1 |
|  | A118 | 27 |  |  | 164 |  | 105 |  | espirapril |  |  | 12 |  |  | 1 |
| Carlsen J.E. et al Drug Investigation. 1991 | A119 | 133 | 53 |  | 165 |  | 103 |  | espirapril |  |  | 24 |  |  | 1 |
|  | A119 | 133 | 54 |  | 163 |  | 104 |  | nitrendipino |  |  | 40 |  |  | 1 |
| Koenig W. et al Drug Investigation 1992 | A120 | 59 | 55 | 10.9 | 173.8 | 12.3 | 104.1 | 5.4 | ramipril |  |  | 2.5 |  |  | 1 |
|  | A120 | 57 | 56 | 10 | 171.8 | 12.8 | 103 | 5.6 | lisinopril |  |  | 10 |  |  | 1 |
|  | A120 | 36 | 55 | 9.3 | 168.3 | 11 | 102.7 | 5.2 | lisinopril |  |  | 5 |  |  | 1 |
| Ruddy MC. Et al Pharmacotherapy. 1993 | A121 | 28 | 50.3 |  | 151.7 |  | 103.6 |  | ramipril |  |  | 2.5 |  |  | 1 |
|  | A121 | 27 | 51.3 |  | 155.2 |  | 103.8 |  | ramipril |  |  | 5 |  |  | 1 |
|  | A121 | 26 | 51.9 |  | 156.7 |  | 104.3 |  | ramipril |  |  | 10 |  |  | 1 |
|  | A121 | 26 | 53 |  | 159.5 |  | 104.5 |  | enalapril |  |  | 5 |  |  | 1 |
|  | A121 | 25 | 51.6 |  | 156.4 |  | 104.9 |  | enalapril |  |  | 10 |  |  | 1 |
|  | A121 | 27 | 53 |  | 157.2 |  | 103.5 |  | enalapril |  |  | 20 |  |  | 1 |
| Witte PU. Et al Am J Cardiol. 1987 | A122 | 129 | 50.4 | 12.4 | 168.5 | 18.5 | 103.4 | 4.8 | ramipril |  |  | 10 |  |  | 1 |
|  | A122 | 119 | 50.9 | 11.5 | 167.2 | 18.2 | 103.3 | 5 | captopril |  |  | 50 |  |  | 2 |
| Heidbreder D. et al Clin Cardiol. 1992 | A123 | 54 | 53.4 | 11.3 | 158.9 | 17.4 | 96.6 | 6.1 | ramipril |  |  | 5 |  |  | 1 |
|  | A123 | 53 | 55 | 10.5 | 160.7 | 16.5 | 97.5 | 6.8 | ramipril |  |  | 10 |  |  | 1 |
|  | A123 | 58 | 54.7 | 8.8 | 160.5 | 16.1 | 97.6 | 6.4 | ramipril | HCTZ |  | 5 | 25 |  | 1 |
| Clementy J. et al Drug Investigation 1991 | A124 | 49 | 68.2 | 5.5 | 183 |  | 104 |  | fosinopril |  |  | 10 |  |  | 1 |
|  | A124 | 50 | 68.8 | 5.8 | 182 |  | 102 |  | nifedipino |  |  | 20 |  |  | 2 |
| Goldstein RJ. Et al Drug Investigation 1991 | A125 | 115 | 51.9 |  | 147.9 |  | 99.9 |  | fosinopril |  |  | 10 |  |  | 1 |
|  | A125 | 116 | 51.7 |  | 150.4 |  | 101 |  | enalapril |  |  | 5 |  |  | 1 |
| Applegate WB. Et al Am J Cardiol. 1996 | A126 | 58 | 54.2 | 10.19 | 152.5 | 13 | 100.4 | 4.8 | placebo |  |  | 0 |  |  | 1 |
|  | A126 | 56 | 52.5 | 11.23 | 152.8 | 17.3 | 100.5 | 5.2 | enalapril |  |  | 5 |  |  | 1 |
| Chan P. et al Am J Hypertens. 1997 | A127 | 25 | 70.9 |  | 167.9 |  | 105.5 |  | placebo |  |  | 0 |  |  | 1 |
|  | A127 | 26 | 72.3 |  | 167.9 |  | 104.5 |  | diltiazem |  |  | 120 |  |  | 1 |
|  | A127 | 26 | 70.5 |  | 170 |  | 105.9 |  | diltiazem |  |  | 240 |  |  | 1 |
|  | A127 | 26 | 72.8 |  | 163.8 |  | 104.9 |  | lisinopril |  |  | 10 |  |  | 1 |
| Chrysant SG. Et al Clin Pharmacol Ther. 1993 | A128 | 59 | 51 | 12 | 153 | 10 | 101 | 5 | placebo |  |  | 0 |  |  | 1 |
|  | A128 | 117 | 55 | 10 | 157 | 16 | 100 | 5 | perindopril |  |  | 4 |  |  | 1 |
|  | A128 | 113 | 53 | 12 | 152 | 15 | 100 | 4 | perindopril |  |  | 2 |  |  | 2 |
| Cushman WC. Et al Am J Hypertens. 1998 | A129 | 150 | 55.8 | 11.4 | 155.4 |  | 101.7 |  | placebo |  |  | 0 |  |  | 1 |
|  | A129 | 144 | 56.1 | 10 | 155.2 |  | 101.6 |  | enalapril |  |  | 5 |  |  | 1 |
|  | A129 | 150 | 53.3 | 10.7 | 152.6 |  | 101.7 |  | diltiazem |  |  | 120 |  |  | 1 |
|  | A129 | 152 | 53.4 | 10.8 | 155.4 |  | 102.2 |  | diltiazem |  |  | 180 |  |  | 1 |
| De Brujin JH. Et al J Cardiovasc Pharmacol. 1994 | A130 | 44 | 50 | 1 | 157.3 |  | 99.2 |  | placebo |  |  | 0 |  |  | 1 |
|  | A130 | 41 | 49 | 2 | 163.8 |  | 99.5 |  | trandolapril |  |  | 0.5 |  |  | 1 |
|  | A130 | 42 | 48 | 2 | 159.9 |  | 99.9 |  | trandolapril |  |  | 1 |  |  | 1 |
|  | A130 | 43 | 46 | 2 | 161.1 |  | 99.8 |  | trandolapril |  |  | 2 |  |  | 1 |
| Drayer JI. Et al Hypertension. 1983 | A131 | 77 | 53 |  | 157 |  | 102 |  | placebo |  |  | 0 |  |  | 2 |
|  | A131 | 77 | 52 |  | 156 |  | 101 |  | captopril |  |  | 25 |  |  | 2 |
|  | A131 | 71 | 52 |  | 154 |  | 101 |  | captopril |  |  | 50 |  |  | 2 |
|  | A131 | 69 | 55 |  | 158 |  | 102 |  | captopril |  |  | 100 |  |  | 2 |
| Gomez HJ. Et al Br J Clin Pharmacol. 1989 | A132 | 47 | 56 |  | 160.5 |  | 100.4 |  | placebo |  |  | 0 |  |  | 1 |
|  | A132 | 41 | 58 |  | 157.6 |  | 100.3 |  | lisinopril |  |  | 1.25 |  |  | 1 |
|  | A132 | 41 | 56 |  | 159 |  | 99.6 |  | lisinopril |  |  | 5 |  |  | 1 |
|  | A132 | 44 | 54 |  | 158.4 |  | 102.4 |  | lisinopril |  |  | 20 |  |  | 1 |
|  | A132 | 43 | 57 |  | 160.6 |  | 101.1 |  | lisinopril |  |  | 80 |  |  | 1 |
| Matthew R. Weir. Journal of Human Hypertens | A133 | 54 | 50.50 | 9.70 | 164.40 | 20.90 | 110.90 | 6.00 | Dilitiazem |  |  | 120.00 |  |  | 2 |
|  | A133 | 56 | 50.70 | 10.00 | 159.00 | 18.40 | 110.00 | 5.50 | Lisinopril |  |  | 20.00 |  |  | 1 |
| Gradman AH. Et al Hypertension. 1995 | A134 | 78 | 53 |  | 157.9 |  | 103.3 |  | placebo |  |  | 0 |  |  | 1 |
|  | A134 | 83 | 53 |  | 155.4 |  | 103.1 |  | enalapril |  |  | 20 |  |  | 1 |
| Guitard C. et al Cardiovasc Drugs Ther. 1997 | A135 | 50 | 56.5 | 8.2 | 161.3 | 18.2 | 98.2 | 6.9 | placebo |  |  | 0 |  |  | 1 |
|  | A135 | 100 | 58 | 7.9 | 161.8 | 16.3 | 99.7 | 6.6 | espirapril |  |  | 6 |  |  | 1 |
|  | A135 | 101 | 56.2 | 9.7 | 163.2 | 16.4 | 99.5 | 6.1 | enalapril |  |  | 20 |  |  | 1 |
| Prager G. et al J Cardiovasc Pharmacol. 1994 | A136 | 53 | 58.1 | 9.5 | 161.4 | 16.5 | 102.1 | 5.7 | placebo |  |  | 0 |  |  | 1 |
|  | A136 | 54 | 55.6 | 10.1 | 162.5 | 15.1 | 102.4 | 5.4 | cilazapril |  |  | 2.5 |  |  | 1 |
|  | A136 | 55 | 55.6 | 10.8 | 158.8 | 16.5 | 100.8 | 4.3 | cilazapril |  |  | 5 |  |  | 1 |
| Sassano P. et al Am J Med. 1984 | A137 | 47 | 46.8 |  | 163.5 | 13.8 | 104.6 | 7 | placebo |  |  | 0 |  |  | 1 |
|  | A137 | 53 | 47.4 |  | 161.4 | 13 | 103.3 | 6.3 | enalapril |  |  | 20 |  |  | 1 |
| Smith DH. Et al J Clin Pharmacol. 2000 | A138 | 43 | 52 |  | 159.5 |  | 104.9 |  | placebo |  |  | 0 |  |  | 1 |
|  | A138 | 42 | 52 |  | 155.3 |  | 103.3 |  | enalapril |  |  | 20 |  |  | 1 |
| VA Study Group Arch Intern Med 1984 | A139 | 83 | 54.4 | 8 | 146.3 |  | 97.8 |  | placebo |  |  | 0 |  |  | 3 |
|  | A139 | 83 | 55.7 | 9.8 | 147.8 |  | 97 |  | captopril |  |  | 12.5 |  |  | 3 |
|  | A139 | 84 | 55.7 | 8.1 | 147.4 |  | 97.9 |  | captopril |  |  | 25 |  |  | 3 |
|  | A139 | 88 | 54.9 | 7.9 | 149 |  | 97.5 |  | captopril |  |  | 37.5 |  |  | 2 |
|  | A139 | 89 | 55.1 | 8 | 148.2 |  | 98.1 |  | captopril |  |  | 50 |  |  | 3 |
| Villamil AS. Et al Am J Cardiol. 1987 | A140 | 27 | 52 |  | 166.6 |  | 101.5 |  | placebo |  |  | 0 |  |  | 1 |
|  | A140 | 28 | 54 |  | 162 |  | 101.1 |  | ramipril |  |  | 2.5 |  |  | 1 |
|  | A140 | 29 | 53 |  | 166.8 |  | 103.2 |  | ramipril |  |  | 5 |  |  | 1 |
| Jensen Aerenlund H. et al Clin. And Exper. Hyper. - Theory and Practice. 1992 | A141 | 108 | 55.9 | 11.9 | 166.3 | 17.5 | 102.9 | 5.8 | lisinopril |  |  | 10 |  |  | 1 |
|  | A141 | 111 | 55 | 13.2 | 166.7 | 18.3 | 103.3 | 5.4 | felodipino |  |  | 5 |  |  | 1 |
| Sánchez S. et al Clin. Ther. 1991 | A142 | 26 | 52.3 | 10.4 | 160 |  | 104 |  | quinapril |  |  | 10 |  |  | 1 |
|  | A142 | 27 | 53.3 | 10 | 159 |  | 105 |  | enalapril |  |  | 10 |  |  | 1 |
| Taylor SH. Et al Angiology. 1989 | A143 | 131 | 53 |  | 160 |  | 101 |  | enalapril |  |  | 10 |  |  | 1 |
|  | A143 | 127 | 55 |  | 162 |  | 103 |  | quinapril |  |  | 10 |  |  | 1 |
| Yasky J. et al BJCP. 1996 | A144 | 30 | 55 |  | 153.7 |  | 96.3 |  | ramipril |  |  | 2.5 |  |  | 1 |
|  | A144 | 30 | 55 |  | 153.7 |  | 96.3 |  | ramipril |  |  | 5 |  |  | 1 |
|  | A144 | 30 | 55 |  | 153.5 |  | 96.2 |  | enalapril |  |  | 10 |  |  | 1 |
|  | A144 | 30 | 55 |  | 153.5 |  | 96.2 |  | enalapril |  |  | 20 |  |  | 1 |
| Hansson L. et al J Cardiovasc Pharmacol. 1996 | A145 | 98 | 54.2 | 1.1 | 157.3 | 1.5 | 100.0 | 0.4 | fosinopril |  |  | 20 |  |  | 1 |
|  | A145 | 97 | 54.4 | 1.1 | 158.6 | 1.7 | 100.2 | 0.3 | enalapril |  |  | 10 |  |  | 1 |
| Pauly NC. Et al J Cardiovasc Pharmacol. 1994 | A146 | 88 | 52 |  | 158.3 | 1.50 | 99.7 | 0.56 | trandolapril |  |  | 4 |  |  | 1 |
|  | A146 | 92 | 54 |  | 159.5 | 1.37 | 100.1 | 0.53 | captopril |  |  | 50 |  |  | 2 |
| Sassano P. et al Am J Med. 1987 | A147 | 100 | 47 | 9 | 160 | 15 | 103 | 8 | placebo |  |  |  |  |  | 1 |
|  | A147 | 105 | 47 | 9 | 162 | 14 | 104 | 7 | enalapril |  |  | 20 |  |  | 1 |
| Ruff D. et al J Hypertens. 1996 | A148 | 50 | 49.8 | 11.6 | 173.7 | 14.5 | 118.5 | 3.5 | losartan |  |  | 50 |  |  | 1 |
|  | A148 | 25 | 53.0 | 11.5 | 176.5 | 14.9 | 119.3 | 3.1 | enalapril |  |  | 20 |  |  | 1 |
| Kellaway GS. Et al Eur J Clin Pharmacol. 1993 | A149 | 47 | 53.8 |  | 161.2 |  | 101.5 |  | cilazapril |  |  | 2.5 |  |  | 1 |
|  | A149 | 40 | 55.5 |  | 158.3 |  | 99.3 |  | cilazapril | HCTZ |  | 2.5 | 12.5 |  | 1 |
| Tikkanen I. et al J Hypertens. 1995 | A150 | 202 |  |  | 156.5 | 16.4 | 102.9 | 6.0 | losartan |  |  | 50 |  |  | 1 |
|  | A150 | 205 |  |  | 158.3 | 16.7 | 103.4 | 5.9 | enalapril |  |  | 20 |  |  | 1 |
| Merrill D. et al AJH 1991 | A151 | 52 | 73.2 |  | 170 |  | 101 |  | placebo |  |  |  |  |  | 1 |
|  | A151 | 55 | 73.9 |  | 168 |  | 101 |  | lisinopril |  |  | 5 |  |  | 1 |
|  | A151 | 53 | 74.0 |  | 168 |  | 101 |  | lisinopril |  |  | 10 |  |  | 1 |
|  | A151 | 54 | 72.8 |  | 171 |  | 101 |  | lisinopril |  |  | 20 |  |  | 1 |
| Poisson P. et al Curr Med Res Opin 1996 | A152 | 213 | 57.6 |  | 164.9 |  | 101 |  | felodipino |  |  | 2.5 |  |  | 1 |
|  | A152 | 213 | 57.7 |  | 165.5 |  | 100.7 |  | ramipril |  |  | 2.5 |  |  | 1 |
|  | A152 | 216 | 57.3 |  | 162.5 |  | 100 |  | felodipino | ramipril |  | 2.5 | 2.5 |  | 1 |
| Gosse P. et al J Hum Hypertens. 1989 | A153 | 150 | 52 | 11 | 165 | 13 | 101 | 4 | lisinopril |  |  | 20 |  |  | 2 |
|  | A153 | 154 | 52 | 10 | 165 | 13 | 102 | 5 | captopril |  |  | 50 |  |  | 1 |
| Andersen K. et al J Hypertens. 2008 | A154 | 420 | 53.4 | 10.8 | 151.3 | 11.7 | 98.8 | 3.4 | Aliskiren |  |  | 300 |  |  | 1 |
|  | A154 | 422 | 53.1 | 11.2 | 151.5 | 11.7 | 98.9 | 3.5 | Ramipril |  |  | 10 |  |  | 1 |
| Zappe DH. Et al J Hum Hypertens. 2010 | A155 | 218 | 52.2 | 10.7 | 160.7 | 7.4 | 97.7 | 5.5 | Valsartan |  |  | 80 |  |  | 1 |
|  | A155 | 221 | 53.1 | 9 | 161.1 | 7.9 | 98.2 | 5.1 | Valsartan |  |  | 160 |  |  | 1 |
|  | A155 | 213 | 52.6 | 10.4 | 161 | 7.8 | 98.2 | 5.1 | Valsartan | HCTZ |  | 160 | 12.5 |  | 1 |
| Roberto Fogari. Clin Drug Investig. 2010 | A156 | 282 | 56.00 | 11.50 | 168.10 | 7.60 | 104.50 | 4.00 | Olmesartan |  |  | 40.00 |  |  | 1 |
|  | A156 | 556 | 55.40 | 10.69 | 168.50 | 8.40 | 104.60 | 4.20 | Olmesartan | HCTZ |  | 40.00 | 12.50 |  | 1 |
| Reif M. et al Am J Cardiol. 1998 | A157 | 64 | 55 | 12 | 154 | 13 | 101 | 5 | Placebo |  |  |  |  |  | 1 |
|  | A157 | 59 | 54 | 10 | 152 | 12 | 99 | 4 | Candesartan Cilexetil |  |  | 2 |  |  | 1 |
|  | A157 | 63 | 55 | 11 | 152 | 17 | 100 | 5 | Candesartan Cilexetil |  |  | 4 |  |  | 1 |
|  | A157 | 60 | 55 | 11 | 154 | 17 | 101 | 6 | Candesartan Cilexetil |  |  | 8 |  |  | 1 |
|  | A157 | 60 | 55 | 11 | 153 | 18 | 100 | 5 | Candesartan Cilexetil |  |  | 16 |  |  | 1 |
|  | A157 | 59 | 55 | 12 | 152 | 17 | 100 | 5 | Candesartan Cilexetil |  |  | 32 |  |  | 1 |
| Vidt DG. Et al J Hum Hypertens. 2001 | A158 | 307 | 55.5 | 9.9 | 153.6 | 11.7 | 100.4 | 4.3 | Candesartan Cilexetil |  |  | 28 |  |  | 1 |
|  | A158 | 304 | 55.1 | 11 | 152.2 | 12.3 | 100.2 | 4.3 | Losartan |  |  | 71.43 |  |  | 1 |
| Critchley J.A.J.H. et al Curr Ther Res Clin Exp 1996 | A159 | 216 | 59.1 | 10.9 | 163.1 | 17.3 | 102.0 | 5.8 | losartan | HCTZ |  | 50 | 12.5 |  | 1 |
|  | A159 | 109 | 59.5 | 11.0 | 162.0 | 17.5 | 102.0 | 5.8 | captopril | HCTZ |  | 50 | 25 |  | 1 |
| Hedner T. et al Am J Hypertens. 1999 | A160 | 273 | 55.2 | 10.5 | 157.8 | 16.3 | 101.9 | 5.2 | Placebo |  |  |  |  |  | 1 |
|  | A160 | 551 | 55.7 | 10.9 | 157 | 16.3 | 101.4 | 4.6 | Valsartan |  |  | 120 |  |  | 1 |
|  | A160 | 545 | 54.9 | 10.5 | 157.4 | 15.9 | 101.6 | 5.1 | Losartan |  |  | 75 |  |  | 1 |
| Andres HimmelMann. Blood Pressure. 2001 | A161 | 196 | 54.50 | 10.10 | 164.60 | 16.00 | 102.20 | 6.20 | Candesartan |  |  | 16.00 |  |  | 1 |
|  | A161 | 194 | 55.70 | 10.70 | 164.30 | 15.80 | 102.10 | 6.10 | Enalapril |  |  | 20.00 |  |  | 1 |
| Pool JL. Et al Clin Ther. 2007 | A162 | 169 | 52.4 | 11.2 | 150.4 | 12.7 | 99.1 | 3.7 | Placebo |  |  |  |  |  | 1 |
|  | A162 | 166 | 52.2 | 11.2 | 149.6 | 13 | 98.9 | 3.2 | Valsartan |  |  | 160 |  |  | 1 |
|  | A162 | 170 | 52.5 | 11.8 | 149.5 | 13.1 | 99 | 4 | Valsartan |  |  | 320 |  |  | 1 |
|  | A162 | 169 | 52.8 | 12.1 | 151.2 | 12.7 | 99.6 | 3.9 | HCTZ |  |  | 12.5 |  |  | 1 |
|  | A162 | 170 | 53.3 | 11.2 | 150 | 12.4 | 98.9 | 3.3 | HCTZ |  |  | 25 |  |  | 1 |
|  | A162 | 168 | 52.9 | 10.4 | 150 | 12 | 99.1 | 3.6 | Valsartan | HCTZ |  | 160 | 12.5 |  | 1 |
|  | A162 | 168 | 52.1 | 11.4 | 150.7 | 12.7 | 99.2 | 3.7 | Valsartan | HCTZ |  | 320 | 12.5 |  | 1 |
|  | A162 | 169 | 53.5 | 11.1 | 152.5 | 11.7 | 99.4 | 3.9 | Valsartan | HCTZ |  | 320 | 25 |  | 1 |
| Pool JL. Et al J Hum Hypertens. 1999 | A163 | 25 | 53 | 9.3 | 156.4 | 17.6 | 101.7 | 4.9 | placebo |  |  | 0 |  |  | 1 |
|  | A163 | 25 | 54.3 | 10.1 | 157.3 | 13.8 | 102.6 | 5.8 | valsartan |  |  | 10 |  |  | 1 |
|  | A163 | 25 | 52.4 | 9.3 | 150.7 | 13.5 | 101.8 | 5.3 | valsartan |  |  | 40 |  |  | 1 |
|  | A163 | 23 | 52.3 | 12.9 | 152.7 | 13.4 | 100.7 | 5 | valsartan |  |  | 80 |  |  | 1 |
|  | A163 | 24 | 52.2 | 10.2 | 155.1 | 15.7 | 101 | 5.2 | valsartan |  |  | 160 |  |  | 1 |
| Baguet JP. Et al Int J Clin Pract. 2006 | A164 | 87 | 54 | 11 | 160 | 14 | 101 | 6 | candesartan |  |  | 8 |  |  | 1 |
|  | A164 | 80 | 56 | 11 | 162 | 16 | 100 | 5 | placebo |  |  | 0 |  |  | 1 |
| George Bakris. Journal Of Clinical Hypertension. 2001 | A165 | 322 | 54.2 | 11.1 | 152.6 | 12.3 | 100.1 | 3.9 | Candesartan |  |  | 16 |  |  | 1 |
|  | A165 | 332 | 54.1 | 10.4 | 152 | 12.6 | 99.9 | 4.2 | Losartan |  |  | 50 |  |  | 1 |
| Giles TD. Et al J Clin Hypertens. 2007 | A166 | 199 | 52.2 | 9.6 | 155.4 | 11.2 | 103.5 | 3.1 | olmesartan |  |  | 20 |  |  | 1 |
|  | A166 | 200 | 51.3 | 10.5 | 155 | 11.5 | 103.6 | 2.8 | losartan |  |  | 50 |  |  | 1 |
|  | A166 | 197 | 52.2 | 10.3 | 154.3 | 10.6 | 103.3 | 3.2 | valsartan |  |  | 80 |  |  | 1 |
|  | A166 | 100 | 52.4 | 10 | 153.9 | 11 | 103.2 | 2.8 | placebo |  |  | 0 |  |  | 1 |
|  | A166 | 189 |  |  | 155.4 | 11.2 | 103.5 | 3.1 | olmesartan |  |  | 40 |  |  | 1 |
|  | A166 | 189 |  |  | 154.3 | 10.6 | 103.3 | 3.2 | valsartan |  |  | 160 |  |  | 1 |
|  | A166 | 94 |  |  | 153.9 | 11 | 103.2 | 2.8 | placebo |  |  | 0 |  |  | 1 |
| Oparil S. et al J Clin Hypertens. 2001 | A167 | 145 | 52.4 | 8.95 | 157 | 13.3 | 104 | 3.5 | olmesartan |  |  | 20 |  |  | 1 |
|  | A167 | 146 | 51.6 | 9.3 | 157 | 11.9 | 104 | 3.5 | losartan |  |  | 50 |  |  | 1 |
|  | A167 | 142 | 51.7 | 9.62 | 155 | 12.1 | 104 | 3.3 | valsartan |  |  | 80 |  |  | 1 |
| Oparil S. et al J Clin Hypertens (Greenwich). 2008 | A168 | 137 | 55.9 | 9.5 | 155.3 | 9.7 | 93.7 | 7.4 | placebo |  |  | 0 |  |  | 1 |
|  | A168 | 137 | 55.9 | 10.8 | 156.9 | 9.2 | 94.2 | 7.7 | olmesartan |  |  | 20 |  |  | 1 |
|  | A168 | 132 | 55.9 | 10.8 | 156.9 | 9.2 | 94.2 | 7.7 | olmesartan |  |  | 40 |  |  | 1 |
| Weir MR. Et al Postgrad Med. 2011 | A169 | 465 | 51.7 | 9.8 | 158.2 | 10.4 | 101.1 | 4 | olmesartan |  |  | 40 |  |  | 1 |
|  | A169 | 469 | 52.1 | 9.8 | 158.3 | 10.2 | 101.3 | 4.1 | losartan |  |  | 100 |  |  | 1 |
| Kuschnir E. et al J Cardiovasc Pharmacol. 2004 | A170 | 100 | 55 |  | 161.2 | 11.4 | 101.8 | 4.9 | losartan | nifedipino |  | 50 | 20 |  | 1 |
|  | A170 | 100 | 56 |  | 161.8 | 13.3 | 102.2 | 5.3 | losartan |  |  | 50 |  |  | 1 |
|  | A170 | 100 | 57 |  | 162.7 | 13.1 | 101.4 | 4.5 | nifedipino |  |  | 20 |  |  | 1 |
| Barrios V. et al Clin Drug Investig. 2007 | A171 | 2306 | 55 | 10.9 | 156.2 | 13.8 | 97.7 | 5.3 | olmesartan |  |  | 20 |  |  | 1 |
|  | A171 | 302 | 54.8 | 10.9 | 160.8 | 14.2 | 100.8 | 5.6 | olmesartan |  |  | 40 |  |  | 1 |
|  | A171 | 325 | 54.9 | 10.1 | 160.8 | 14 | 100.2 | 5.1 | olmesartan | HCTZ |  | 20 | 12.5 |  | 1 |
| Holwerda NJ. Et al J Hypertens. 1996 | A172 | 137 | 53.1 | 12.4 | 161.7 | 11.6 | 101.2 | 4.5 | valsartan |  |  | 80 |  |  | 1 |
|  | A172 | 69 | 52.5 | 10.3 | 161.5 | 10.4 | 102.2 | 4.2 | enalapril |  |  | 20 |  |  | 1 |
|  | A172 | 142 | 53.1 | 12.9 | 161 | 11.5 | 101.8 | 4.4 | placebo |  |  | 0 |  |  | 1 |
| Lacourcière Y. et al Clin Ther. 2005 | A173 | 261 | 60.4 | 10.6 | 167.9 | 8 | 93.2 | 8.9 | valsartan |  |  | 160 |  |  | 1 |
|  | A173 | 254 | 60.8 | 11.5 | 167.4 | 8.3 | 93.4 | 9.6 | valsartan | HCTZ |  | 160 | 12.5 |  | 1 |
|  | A173 | 252 | 60.7 | 11.6 | 167.2 | 7.9 | 93.7 | 8.8 | valsartan | HCTZ |  | 160 | 25 |  | 1 |
| Sellin L. et al J Hypertens. 2005 | A174 | 174 | 54 | 9.5 | 162.9 |  | 104.5 |  | olmesartan |  |  | 20 |  |  | 1 |
|  | A174 | 184 | 52.1 | 9.9 | 162.4 |  | 104.5 |  | olmesartan | HCTZ |  | 20 | 12.5 |  | 1 |
|  | A174 | 177 | 53.6 | 10 | 163.7 |  | 104.7 |  | olmesartan | HCTZ |  | 20 | 25 |  | 1 |
| Destro M. et al Adv Ther. 2005 | A175 | 52 |  |  | 162.5 | 8.5 | 103.2 | 3.9 | olmesartan |  |  | 20 |  |  | 1 |
|  | A175 | 55 |  |  | 163.7 | 6.4 | 103.5 | 3.8 | valsartan |  |  | 160 |  |  | 1 |
| Oparil S. et al Clin Ther. 1996 | A176 | 148 | 53.6 |  | 152.4 |  | 100.8 |  | placebo |  |  | 0 |  |  | 1 |
|  | A176 | 140 | 53.8 |  | 151.6 |  | 100.8 |  | valsartan |  |  | 20 |  |  | 1 |
|  | A176 | 150 | 53.6 |  | 152.1 |  | 100.9 |  | valsartan |  |  | 80 |  |  | 1 |
|  | A176 | 148 | 52 |  | 149.9 |  | 101.4 |  | valsartan |  |  | 160 |  |  | 1 |
|  | A176 | 150 | 53.7 |  | 151 |  | 101.3 |  | valsartan |  |  | 320 |  |  | 1 |
| Brunner HR. Et al Clin Drug Investig. 2003 | A177 | 312 | 51.5 | 10.5 | 162.4 | 9.4 | 104.8 | 3.7 | olmesartan |  |  | 20 |  |  | 1 |
|  | A177 | 323 | 51.8 | 9.8 | 162.4 | 9.2 | 104.3 | 3.5 | candesartan |  |  | 8 |  |  | 1 |
| Palatini P. et al J Hypertens. 2001 | A178 | 342 |  |  | 168.0 | 9.9 | 101.0 | 4.5 | valsartan |  |  | 80 |  |  | 1 |
|  | A178 | 348 |  |  | 168.1 | 9.9 | 100.7 | 5.1 | amlodipino |  |  | 4 |  |  | 1 |
| Gradman AH. Et al Heart Dis. 1999 | A179 | 162 | 54 | 11 | 152.9 |  | 100.3 |  | candesartan cilexetil |  |  | 16 |  |  | 1 |
|  | A179 | 170 | 57 | 10 | 154.1 |  | 100.5 |  | losartan |  |  | 50 |  |  | 1 |
| Oparil S. et al Curr Med Res Opin. 2008 | A180 | 127 | 50.1 | 10.4 | 151.6 | 8.2 | 99.1 | 4.2 | losartan |  |  | 50 |  |  | 1 |
|  | A180 | 134 | 51.6 | 9.7 | 152.4 | 9.0 | 99.0 | 4.0 | placebo |  |  |  |  |  | 1 |
| Calhoun DA. Et al Curr Med Res Opin. 2008 | A181 | 307 | 51.6 | 10.5 | 168.7 | 14 | 112.2 | 3.1 | valsartan | HCTZ |  | 160 | 12.5 |  | 1 |
|  | A181 | 301 | 51.6 | 11.2 | 168.2 | 13.7 | 112.4 | 2.9 | valsartan |  |  | 160 |  |  | 1 |
| Mallion JM. Et al J Hypertens Suppl. 1995 | A182 | 109 | 53.5 |  | 159.3 | 16.8 | 103.1 | 5.3 | losartan |  |  | 50 |  |  | 1 |
|  | A182 | 54 | 55.3 |  | 159.4 | 16.2 | 103.7 | 5.5 | captopril |  |  | 50 |  |  | 1 |
| Luccioni R. et al . J Hypertens. 1995 | A183 | 175 | 54.4 | 11.1 |  |  | 101.2 | 4.6 | perindopril | indapamida |  | 4 | 1.25 |  | 1 |
|  | A183 | 177 | 54.5 | 11.2 |  |  | 101.5 | 4.5 | enalapril | HCTZ |  | 20 | 12.5 |  | 1 |
| Mallion J. et al J Hum Hypertens. 1999 | A184 | 55 | 54 |  | 156.5 | 14.7 | 99.2 | 3.9 | placebo |  |  |  |  |  | 1 |
|  | A184 | 57 | 56 |  | 162.4 | 16.3 | 100.7 | 4.5 | losartan |  |  | 50 |  |  | 1 |
|  | A184 | 57 | 58 |  | 161.9 | 14.7 | 100.8 | 4.2 | telmisartan |  |  | 40 |  |  | 1 |
|  | A184 | 54 | 57 |  | 164.2 | 15.3 | 101.8 | 4.9 | telmisartan |  |  | 80 |  |  | 1 |
| Mancia G. et al Blood Press Monit. 2002 | A185 | 215 | 55.4 | 9.9 | 158 |  | 100.8 |  | valsartan |  |  | 80 |  |  | 1 |
|  | A185 | 211 | 55.1 | 9.7 | 159.3 |  | 100.7 |  | irbesartan |  |  | 150 |  |  | 1 |
| Kassler-Taub K. et al Am J Hypertens 1998 | A186 | 147 | 53.8 | 9.6 | 152.4 | 14.7 | 100.3 | 4.3 | Placebo |  |  |  |  |  | 1 |
|  | A186 | 138 | 55 | 10.7 | 153.3 | 15.5 | 100.6 | 4.4 | Losartan |  |  | 100 |  |  | 1 |
|  | A186 | 142 | 53.1 | 10.5 | 155.3 | 16.2 | 101.1 | 4.6 | Ibesartan |  |  | 150 |  |  | 1 |
|  | A186 | 140 | 55.6 | 10.4 | 155.4 | 16 | 100.4 | 4.5 | Ibesartan |  |  | 300 |  |  | 1 |
| Elliot WJ. Et al Clin Ther. 2001 | A187 | 247 | 53 | 10 | 152.8 |  | 100.9 |  | losartan |  |  | 50 |  |  | 1 |
|  | A187 | 248 | 54 | 10 | 151.9 |  | 100.5 |  | valsartan |  |  | 80 |  |  | 1 |
| Andersson OK. ET AL Blood Press. 1998 | A188 | 85 | 60 | 10 | 170 | 14 | 103 | 5 | placebo |  |  |  |  |  | 1 |
|  | A188 | 82 | 60 | 11 | 169 | 14 | 102 | 5 | candesartan cilexetil |  |  | 8 |  |  | 1 |
|  | A188 | 84 | 59 | 10 | 168 | 15 | 103 | 5 | candesartan cilexetil |  |  | 16 |  |  | 1 |
|  | A188 | 83 | 59 | 9 | 168 | 16 | 104 | 5 | losartan |  |  | 50 |  |  | 1 |
| Monterroso VH. Et al Adv Ther. 2000 | A189 | 93 | 54.6 | 11.0 |  |  | 100.8 | 5.6 | losartan |  |  | 50 |  |  | 1 |
|  | A189 | 94 | 54.1 | 8.3 |  |  | 100.8 | 6.6 | valsartan |  |  | 80 |  |  | 1 |
| Lacourcière Y. et al Am J Hypertens. 1999 | A190 | 37 |  |  | 163.3 |  | 99.7 |  | placebo |  |  |  |  |  | 1 |
|  | A190 | 116 |  |  | 162.1 |  | 101.3 |  | candesartan cilexetil |  |  | 8 |  |  | 1 |
|  | A190 | 115 |  |  | 160.6 |  | 100.1 |  | losartan |  |  | 50 |  |  | 1 |
| Smith DH. Et al Am J Cardiovasc Drugs. 2005 | A191 | 136 | 52.3 |  | 152.1 |  | 94.3 |  | olmesartan medoxomil |  |  | 20 |  |  | 1 |
|  | A191 | 134 | 52.0 |  | 152.3 |  | 95.2 |  | losartan |  |  | 50 |  |  | 1 |
|  | A191 | 130 | 51.9 |  | 152.2 |  | 94.7 |  | valsartan |  |  | 80 |  |  | 1 |
|  | A191 | 134 | 52.1 |  | 151.6 |  | 94.8 |  | irbesartan |  |  | 150 |  |  | 1 |
| White WB. Et al Blood Press Monit. 2008 | A192 | 130 | 54 | 10 | 152.2 | 11.9 | 101.3 | 4 | Placebo |  |  |  |  |  | 1 |
|  | A192 | 528 | 53 | 10 | 154.1 | 12.6 | 101.8 | 4.2 | Telmisartan | HCTZ |  | 80 | 18.75 |  | 1 |
|  | A192 | 523 | 53 | 10 | 154.9 | 12.5 | 101.6 | 4.3 | Valsartan | HCTZ |  | 160 | 18.75 |  | 1 |
| Flack JM. Et al Clin Ther. 2001 | A193 | 188 | 50.6 | 10.2 | 151.4 | 12.1 | 99.8 | 3.9 | Placebo |  |  |  |  |  | 1 |
|  | A193 | 193 | 50.4 | 10.5 | 149.1 | 11.3 | 100.2 | 11.3 | Losartan |  |  | 50 |  |  | 1 |
|  | A193 | 59 | 47.2 | 9.8 | 150.9 | 10.6 | 99.9 | 10.6 | Losartan |  |  | 50 |  |  | 1 |
| Fogari R. et al Eur J Clin Pharmacol. 2004 | A194 | 73 | 70.37 | 5.7 | 165.9 | 7.3 | 100.8 | 3.7 | Valsartan |  |  | 160 |  |  | 1 |
|  | A194 | 71 | 70.34 | 5.7 | 165.8 | 6.8 | 100.9 | 3.9 | Enalapril |  |  | 20 |  |  | 1 |
| Black HR. Et al . J Clin Hypertens (Greenwich) 2011 | A195 | 220 | 53.2 | 9.3 | 167.4 | 8.2 | 96.2 | 9.5 | Aliskiren | Amlodipino |  | 281.25 | 9.38 |  | 1 |
|  | A195 | 223 | 52.4 | 10.6 | 167.4 | 8.1 | 97.2 | 8.2 | Amlodipino |  |  | 9.38 |  |  | 1 |
| de Bruijn B. et al J Cardiovasc Pharmacol. 1988 | A196 | 64 | 53 |  | 164 | 2 | 102 | 1 | Placebo |  |  |  |  |  | 1 |
|  | A196 | 65 | 54.7 |  | 165 | 2 | 102 | 1 | Amlodipino |  |  | 7.7 |  |  | 1 |
|  | A196 | 78 | 51.9 |  | 162 | 2 | 102 | 1 | Atenolol |  |  | 69.9 |  |  | 1 |
| W Krone. Journal of Human Hypertension. 2011 | A197 | 75 | 58.6 | 8.9 | 155.8 | 12.1 | 94.1 | 7.8 | Aliskiren |  |  | 300 |  |  | 1 |
|  | A197 | 66 | 59.2 | 9.1 | 154.2 | 13.8 | 91.5 | 9.9 | Irbesartan |  |  | 300 |  |  | 1 |
| Hayduk K. et al Blood Press Suppl. 1994 | A198 | 40 | 53.8 | 9.8 | 160 | 17.9 | 104.5 | 3.7 | espirapril |  |  | 1 |  |  | 1 |
|  | A198 | 44 | 53.4 | 9.7 | 160 | 14.3 | 106.4 | 5.4 | espirapril |  |  | 6 |  |  | 1 |
|  | A198 | 44 | 53.1 | 8.5 | 159.3 | 15.6 | 104.8 | 4.6 | espirapril |  |  | 12 |  |  | 1 |
|  | A198 | 43 | 53.7 | 10.1 | 161 | 17.9 | 106.1 | 4.4 | espirapril |  |  | 24 |  |  | 1 |
| Lorimer AR. Et al J Cardiovasc Pharmacol. 1988 | A199 | 53 | 55.1 |  | 163.8 |  | 103.9 |  | amlodipino |  |  | 10 |  |  | 1 |
|  | A199 | 54 | 55.7 |  | 170.3 |  | 104.2 |  | verapamil |  |  | 160 |  |  | 2 |
|  | A199 | 53 | 55.5 |  | 163.4 |  | 103 |  | placebo |  |  | 0 |  |  | 1 |
| Smith DH. Et al J Clin Pharmacol. 2000 | A200 | 43 | 52 |  | 159.5 |  | 104.9 |  | placebo |  |  | 0 |  |  | 1 |
|  | A200 | 40 | 54.3 |  | 154.6 |  | 102.4 |  | telmisartan |  |  | 40 |  |  | 1 |
|  | A200 | 41 | 50.6 |  | 154.2 |  | 103.1 |  | telmisartan |  |  | 80 |  |  | 1 |
|  | A200 | 41 | 52 |  | 153.9 |  | 102 |  | telmisartan |  |  | 120 |  |  | 1 |
|  | A200 | 42 | 52 |  | 155.3 |  | 103.3 |  | enalapril |  |  | 20 |  |  | 1 |
| Ollivier JP. Et al Cardiology. 1995 | A201 | 78 | 57.3 | 1.1 | 167 | 2 | 101 | 1 | diltiazem |  |  | 200 |  |  | 1 |
|  | A201 | 80 | 56.4 | 1.0 | 166 | 2 | 101 | 1 | placebo |  |  |  |  |  | 1 |
| Michael A.Weber. Clinical Pharmacol Therapeutics. 1994 | A202 | 68 | 55 |  | 154.4 |  | 101 |  | Placebo |  |  |  |  |  | 1 |
|  | A202 | 71 | 55 |  | 151.4 |  | 100.6 |  | Felodipino |  |  | 2.5 |  |  | 1 |
|  | A202 | 72 | 55 |  | 147.9 |  | 100.6 |  | Felodipino |  |  | 5 |  |  | 1 |
|  | A202 | 70 | 55 |  | 153.8 |  | 101.2 |  | Felodipino |  |  | 10 |  |  | 1 |
| Oparil S. et al Lancet. 2007 | A203 | 459 | 52.6 | 10.4 | 154.1 | 12.8 | 100.4 | 4.2 | Placebo |  |  |  |  |  | 1 |
|  | A203 | 437 | 51.9 | 10.9 | 153.9 | 11.7 | 100.2 | 3.9 | Aliskiren |  |  | 225 |  |  | 1 |
|  | A203 | 455 | 52.4 | 10.4 | 154.2 | 12.7 | 100.3 | 3.8 | Valsartan |  |  | 240 |  |  | 1 |
|  | A203 | 446 | 52.1 | 10.3 | 152.8 | 12.2 | 100.1 | 4 | Aliskiren | Valsartan |  | 225 | 240 |  | 1 |
| Os I. et al Am J Hypertens. 1997 | A204 | 186 | 51 |  | 159.1 | 15.5 | 102.7 | 5.5 | Enalapril | HCTZ |  | 20 | 6 |  | 1 |
|  | A204 | 188 | 51.6 |  | 159.9 | 14.9 | 102.4 | 4.9 | Atenolol |  |  | 50 |  |  | 1 |
| Chanudet X. et al Int J Clin Pract. 2001 | A205 | 146 | 59 | 11.4 | 165.2 | 10.9 | 97.7 | 7.8 | perindopril | indapamida |  | 2 | 0.625 |  | 1 |
|  | A205 | 131 | 58.9 | 12.8 | 165.7 | 12.6 | 98.7 | 7.5 | losartan |  |  | 50 |  |  | 1 |
| Chalmers J. et al Am J Hypertens. 1989 | A206 | 190 | 71.8 | 5.6 | 171.6 | 10.7 | 100.5 | 3.8 | placebo |  |  | 0 |  |  | 1 |
|  | A206 | 193 | 72.9 | 5.3 | 171.1 | 11.4 | 95.1 | 7.7 | perindopril | indapamida |  | 2 | 0.625 |  | 1 |
| Lessemn JN. Et al Am J Hypertens. 1989 | A207 | 68 |  |  | 154.8 |  | 101 |  | nicardipino |  |  | 30 |  |  | 3 |
|  | A207 | 70 |  |  | 153.7 |  | 100.8 |  | propranolol |  |  | 40 |  |  | 3 |
|  | A207 | 68 |  |  | 154.6 |  | 99.9 |  | propranolol | nicardipino |  | 40 | 30 |  | 3 |
| Maclean D. et al Angiology. 1989 | A208 | 89 | 52 |  | 162 |  | 105 |  | placebo |  |  | 0 |  |  | 1 |
|  | A208 | 90 | 51 |  | 164 |  | 106 |  | quinapril |  |  | 80 |  |  | 2 |
|  | A208 | 91 | 49 |  | 163 |  | 107 |  | quinapril |  |  | 80 |  |  | 1 |

**Supplementary Table 5. Excluded studies**

Exclusion cause:

1. Insufficient sample size

2. Specific hypertensive population different to the ones defined

3. Non-available essential data

4. Inadequate endpoint variables

5. Study design different from randomized double blind clinical trial

6. Other

| **Ref. #** | **Reference** | **Exclusion cause** | |
| --- | --- | --- | --- |
| e1 | Horwitz LD, Weinberger HD, Clegg L. Comparison of amlodipine and long-acting diltiazem in the treatment of mild or moderate hypertension. Am J Hypertens. 1997;10:1263-9 | | 5 |
| e2 | Puig JG, Marre M, Kokot F, Fernandez M, Jermendy G, Opie L, Moyseev V, Scheen A, Ionescu-Tirgoviste C, Saldanha MH, Halabe A, Williams B, Mion D Jr, Ruiz M, Hermansen K, Tuomilehto J, Finizola B, Gallois Y, Amouyel P, Ollivier JP, Asmar R. Efficacy of indapamide SR compared with enalapril in elderly hypertensive patients with type 2 diabetes. Am J Hypertens. 2007;20:90-7 | | 5 |
| e3 | The Heart Outcomes Prevention Evaluation Study Investigators. Effects of an Angiotensin-Converting–Enzyme Inhibitor, Ramipril, on Cardiovascular Events in High-Risk Patients. N Engl J Med 2000; 342:145-153 | | 5 |
| e4 | Gavras H, Chrysant SG, Niederman AL, Marbury TC, Goldstein R, Conradi E. Evaluation of enalapril combined with diltiazem ER in patients with stage 3-4 essential hypertension. Clin Exp Hypertens. 1998;20:41-52 | | 5 |
| e5 | Bönner G, Fuchs W. Fixed combination of candesartan with hydrochlorothiazide in patients with severe primary hypertension. Curr Med Res Opin. 2004;20+E34:597-602 | | 5 |
| e6 | Fowler G, Webster J, Lyons D, Witte K, Crichton WA, Jeffers TA, Wickham EA, Sanghera SS, Cornish R, Petrie JC. A comparison of amlodipine with enalapril in the treatment of moderate/severe hypertension. Br J Clin Pharmacol 1993; 35: 491–498 | | 1, 5 |
| e7 | Messerli FH, Oparil S, Feng Z. Comparison of efficacy and side effects of combination therapy of angiotensin-converting enzyme inhibitor (benazepril) with calcium antagonist (either nifedipine or amlodipine) versus high-dose calcium antagonist monotherapy for systemic hypertension. Am J Cardiol. 20001;86:1182-7 | | 5 |
| e8 | Zhu JR, Sun NL, Yang K, Hu J, Xu G, Hong H, Wang R, Tu YM, Ritter S, Keefe D; trial investigators. Efficacy and safety of aliskiren, a direct renin inhibitor, compared with ramipril in Asian patients with mild to moderate hypertension. Hypertens Res. 2012;35:28-3 | | 2 |
| e9 | Devi P, Xavier D, Sigamani A, Pandey S, Thomas T, Murthy S, Sharma K, Bosco B, Mehta K, Joshi S, Gupta R, Singh G, Hiremath J, Ds C, Nambiar A, Pais P. Effect of fixed dose combinations of metoprolol and amlodipine in essential hypertension: MARS--a randomized controlled trial. Blood Press Suppl. 2011;2:5-12 | | 2 |
| e10 | Jones DW, Sands CD. Treatment of essential hypertension in Asians: enalapril as monotherapy versus combination therapy with hydrochlorothiazide. Pharmacotherapy. 1991;11:127-30 | | 2 |
| e11 | Kushiro T, Itakura H, Abo Y, Gotou H, Terao S, Keefe DL. Aliskiren, a Novel Oral Renin Inhibitor, Provides Dose-Dependent Efficacy and Placebo-Like Tolerability in Japanese Patients with Hypertension. Hypertens Res. 2006;29:997-1005 | | 2, 6 |
| e12 | Woo KS, Pun CO. Long-acting nifedipine versus metoprolol as monotherapy for essential hypertension. A randomized, controlled crossover study . West J Med. 1990;152:149-52 | | 1 |
| e13 | Grimm RH Jr, Black H, Rowen R, Lewin A, Shi H, Ghadanfar M; Amlodipine Study Group. Amlodipine versus chlorthalidone versus placebo in the treatment of stage I isolated systolic hypertension. Am J Hypertens. 2002;15:31-6 | | 2 |
| e14 | Staessen JA, Fagard R, Thijs L, Celis H, Arabidze GG, Birkenhäger WH, Bulpitt CJ, de Leeuw PW, Dollery CT, Fletcher AE, Forette F, Leonetti G, Nachev C, O'Brien ET, Rosenfeld J, Rodicio JL, Tuomilehto J, Zanchetti A. Randomised double-blind comparison of placebo and active treatment for older patients with isolated systolic hypertension. The Systolic Hypertension in Europe (Syst-Eur) Trial Investigators. Lancet. 1997, 13;350:757-64 | | 2,4 |
| e15 | Emeriau JP, Knauf H, Pujadas JO, Calvo-Gomez C, Abate G, Leonetti G, Chastang C; European Study Investigators. A comparison of indapamide SR 1.5 mg with both amlodipine 5 mg and hydrochlorothiazide 25 mg in elderly hypertensive patients: a randomized double-blind controlled study. J Hypertens. 2001;19:343-50 | | 2 |
| e16 | [No authors listed]. Cardiovascular risk and risk factors in a randomized trial of treatment based on the beta-blocker oxprenolol: the International Prospective Primary Prevention Study in Hypertension (IPPPSH). J Hypertens. 1985;3:379-92 | | 2 |
| e17 | Benetos A, Consoli S, Safavian A, Dubanchet A, Safar M. Efficacy, safety, and effects on quality of life of bisoprolol/hydrochlorothiazide versus amlodipine in elderly patients with systolic hypertension. m Heart J. 2000;140:E11 | | 2 |
| e18 | Soininen K, Gerlin-Piira L, Suihkonen J, Kyllönen T, Parviainen R, Kyllönen E, Hämäläinen K, Lonka R, Tikkanen T, Selonen R, et al. A study of the effects of lisinopril when used in addition to atenolol. J Hum Hypertens. 1992;6:321-4 | | 1, 2 |
| e19 | Pathé M. Lisinopril-hydrochlorothiazide combination vs lisinopril for the treatment of hypertension. J Hum Hypertens. 1991;5 Suppl 2:53-4 | | 2 |
| e20 | Lacourcière Y, Tytus R, O'Keefe D, Lenis J, Orchard R, Martin K. Efficacy and tolerability of a fixed-dose combination of telmisartan plus hydrochlorothiazide in patients uncontrolled with telmisartan monotherapy. J Hum Hypertens. 2001;15:763-70 | | 2 |
| e21 | Geiger H, Barranco E, Gorostidi M, Taylor A, Zhang X, Xiang Z, Zhang J. Combination therapy with various combinations of aliskiren, valsartan, and hydrochlorothiazide in hypertensive patients not adequately responsive to hydrochlorothiazide alone. J Clin Hypertens (Greenwich). 2009;11:324-32 | | 2 |
| e22 | Imbs JL, Nisse-Durgeat S; French Collaborative Candesartan Study Group. Efficacy and tolerability of candesartan cilexetil vs. amlodipine as assessed by home blood pressure in hypertensive patients. Int J Clin Pract. 2005 ;59:78-84 | | 2 |
| e23 | Hair PI, Scott LJ, Perry CM. Fixed-dose combination lercanidipine/enalapril. Drugs. 2007;67:95-106; discussion 107-8 | | 2 |
| e24 | Guerrero P, Fuchs FD, Moreira LM, Martins VM, Bertoluci C, Fuchs SC, Gus M. Blood pressure-lowering efficacy of amiloride versus enalapril as add-on drugs in patients with uncontrolled blood pressure receiving hydrochlorothiazide. Clin Exp Hypertens. 2008;30:553-64 | | 2 |
| e25 | Fogari R, Zoppi A, Mugellini A, Preti P, Destro M, Rinaldi A, Derosa G. Hydrochlorothiazide added to valsartan is more effective than when added to olmesartan in reducing blood pressure in moderately hypertensive patients inadequately controlled by monotherapy. Adv Ther. 2006;23:680-95 | | 2 |
| e26 | Hasebe N, Kikuchi K; NICE Combi Study Group. Controlled-release nifedipine and candesartan low-dose combination therapy in patients with essential hypertension: the NICE Combi (Nifedipine and Candesartan Combination) Study. J Hypertens. 2005;23:445-53 | | 2 |
| e27 | Axthelm C, Sieder C, Meister F, Kaiser E. Efficacy and tolerability of the single-pill combination of aliskiren 300 mg/amlodipine 10 mg in hypertensive patients not controlled by olmesartan 40 mg/amlodipine 10 mg. Curr Med Res Opin. 2012;28:69-78 | | 2 |
| e28 | Brachmann J, Ansari A, Mahla G, Handrock R, Klebs S. Effective and safe reduction of blood pressure with the combination of amlodipine 5 mg and valsartan 160 mg in hypertensive patients not controlled by calcium channel blocker monotherapy. Adv Ther. 2008;25:399-411 | | 2 |
| e29 | Chrysant SG, Sugimoto DH, Lefkowitz M, Salko T, Khan M, Arora V, Shi V. The effects of high-dose amlodipine/benazepril combination therapies on blood pressure reduction in patients not adequately controlled with amlodipine monotherapy. Blood Press Suppl. 2007;1:10-7 | | 2 |
| e30 | Allemann Y, Fraile B, Lambert M, Barbier M, Ferber P, Izzo JL Jr. Efficacy of the combination of amlodipine and valsartan in patients with hypertension uncontrolled with previous monotherapy: the Exforge in Failure after Single Therapy (EX-FAST) study. J Clin Hypertens (Greenwich). 2008;10:185-94 | | 2 |
| e31 | Agabiti-Rosei E, Ambrosioni E, Finardi G, Folino P, Gambassi G, Malini P, Marchesi E, Muiesan ML, Semplicini A, Pessina AC. Perindopril versus captopril: efficacy and acceptability in an Italian multicenter trial. Am J Med. 1992, 27;92:79S-83S | | 3 |
| e32 | Vlasses PH, Rotmensch HH, Swanson BN, Irvin JD, Lee RB, Koplin JR, Ferguson RK. Comparative antihypertensive effects of enalapril maleate and hydrochlorothiazide, alone and in combination. J Clin Pharmacol. 1983;23:227-33 | | 1 |
| e33 | Pandhi P, Sethi V, Sharma BK, Wahi PL, Sharma PL. Double blind cross-over clinical trial of acebutolol and propranolol in angina pectoris. Int J Clin Pharmacol Ther Toxicol. 1985;23:598-600 | | 1 |
| e34 | Mancia G, Grassi G. Antihypertensive effects of combined lisinopril and hydrochlorothiazide in elderly patients with systodiastolic or systolic hypertension: results of a multicenter trial. J Cardiovasc Pharmacol. 1997 ;30:548-53 | | 6 |
| e35 | Woo J, Woo KS, Vallance-Owen J. Captopril versus hydrochlorothiazide/triamterene in mild-to-moderate hypertension in the elderly. Lancet. 1986;2:924 | | 1 |
| e36 | Thulin T, Hedner T, Gustafsson S, Olsson SO. Diltiazem compared with metoprolol as add-on-therapies to diuretics in hypertension. J Hum Hypertens. 1991;5:107-14 | | 3 |
| e37 | Silke B, Guy S, Humphreys JE. Comparison of antihypertensive and lipid actions of terazosin and atenolol in essential hypertension. J Hum Hypertens. 1992;6:221-5 | | 1,3 |
| e38 | [No authors listed] Comparison of propranolol and hydrochlorothiazide for the initial treatment of hypertension. II. Results of long-term therapy. Veterans Administration. JAMA. 1982;248:2004-11 | | 3 |
| e39 | Trenkwalder P, Schaetzl R, Borbas E, Handrock R, Klebs S. Combination of amlodipine 10 mg and valsartan 160 mg lowers blood pressure in patients with hypertension not controlled by an ACE inhibitor/CCB combination. Blood Press Suppl. 2008;2:13-21 | | 2, 3 |
| e40 | Mancia G, Omboni S; CARDIO (CAndesaRtan combined with DIuretic in hypertensiOn) Study Group. Candesartan plus hydrochlorothiazide fixed combination vs previous monotherapy plus diuretic in poorly controlled essential hypertensive patients. Blood Press Suppl. 2004;2:11-7 | | 5 |
| e41 | Weir RJ, Lee PS, Clegg DS, Hemingray S, Belgrave GP, Walter E. A multicentre study to compare the therapeutic efficacy of sustained-release diltiazem and enalapril in the treatment of patients with mild to moderate hypertension. Br J Clin Pract. 1994;48:287-92 | | 3 |
| e42 | van der Byl KV, Maharaj B, Leary WP. A comparison of captopril and atenolol in addition to hydrochlorothiazide for the treatment of hypertension. S Afr Med J 1988; 74 :425 | | 1 |
| e43 | Zachariah PK, Sheps SG, Oshrain C, Schirger A, Stein WJ. Antihypertensive efficacy of sustained-release verapamil. J Clin Hypertens. 1987 ;3:536-46 | | 1 |
| e44 | Smith DH, Neutel JM, Jankelow D, Pretorius JJ, Myburgh DP. A comparative study of atenolol, nifedipine and their combination in the treatment of hypertension. S Afr Med J. 1991 Jan 5;79:12-5 | | 1 |
| e45 | Veterans Administration Cooperative Study Groups on Antihypertensive Agents. Low-dose captopril therapy in mild and moderate hypertension. Hypertension. 1983;5:III139-144 | | 1 |
| e46 | Smith DH, Neutel JM, Black HR, Schoenberger JA, Weber MA. Once-daily monotherapy with trandolapril in the treatment of hypertension. J Hum Hypertens. 1996;10:129-34 | | 3 |
| e47 | Ruilope LM. Comparison of a new vasodilating beta-blocker, carvedilol, with atenolol in the treatment of mild to moderate essential hypertension. Am J Hypertens. 1994;7:129-36 | | 3 |
| e48 | Yoneda S, Kako T, Koketsu M, Hayano J, Asakawa T, Fujinami T, Kato T. Single administration of captopril and combined use with beta-blocker and/or thiazide diuretic in the treatment of essential hypertension. Angiology. 1991;42:914-23 | | 2 |
| e49 | Zachariah PK. Nitrendipine as an antihypertensive agent. Eur Heart J. 1987;8 Suppl K:63-7 | | 3 |
| e50 | Olvera R, Samaniego V, Moguel R, Meaney E. Efficacy and tolerability of amlodipine in patients with mild-to-moderate hypertension. Int J Clin Pharmacol Res. 1991;11:237-41 | | 1 |
| e51 | Ruilope L, Jäger B, Prichard B. Eprosartan versus enalapril in elderly patients with hypertension: a double-blind, randomized trial. Blood Press. 2001;10:223-9 | | 3 |
| e52 | Punzi H, Lewin A, Lukić T, Goodin T, Wei Chen. Efficacy and safety of nebivolol in Hispanics with stage I-II hypertension: a randomized placebo-controlled trial. Ther Adv Cardiovasc Dis. 2010;4:349-57 | | 3 |
| e53 | Philipp T, Glazer RD, Wernsing M, Yen J. Initial combination therapy with amlodipine/valsartan compared with monotherapy in the treatment of hypertension. J Am Soc Hypertens. 2011;5:417-24 | | 3 |
| e54 | Papademetriou V, Prisant LM, Neutel JM, Weir MR. Efficacy of low-dose combination of bisoprolol/hydrochlorothiazide compared with amlodipine and enalapril in men and women with essential hypertension. Am J Cardiol. 1981;81:1363-5 | | 5 |
| e55 | Pareek A, Chandurkar NB, Sharma R, Tiwari D, Gupta BS. Efficacy and tolerability of a fixed-dose combination of metoprolol extended release/amlodipine in patients with mild-to-moderate hypertension: a randomized, parallel-group, multicentre comparison with losartan plus amlodipine. Clin Drug Investig. 2010;30:123-31 | | 2 |
| e56 | Oparil S, Aurup P, Snavely D, Goldberg A. Efficacy and safety of losartan/hydrochlorothiazide in patients with severe hypertension. Am J Cardiol. 2001;87:721-6 | | 3 |
| e57 | Nissinen A, Tuomilehto J. Evaluation of the antihypertensive effect of atenolol in fixed or free combination with chlorthalidone. Pharmatherapeutica. 1980;2:462-8 | | 1 |
| e58 | Kundu SC, Bhattacharya A, Vakil HB. Ramipril and methyldopa compared in patients with mild to moderate hypertension. Clin Ther. 1990;12:393-7 | | 3 |
| e59 | Mengden T, Hübner R, Bramlage P. Office and ambulatory blood pressure control with a fixed-dose combination of candesartan and hydrochlorothiazide in previously uncontrolled hypertensive patients: results of CHILI CU Soon. Vasc Health Risk Manag. 2011;7:761-9 | | 3 |
| e60 | Imbs JL, Nisse-Durgeat S; French Collaborative Candesartan Study Group. Efficacy and tolerability of candesartan cilexetil vs. amlodipine as assessed by home blood pressure in hypertensive patients. Int J Clin Pract. 2005;59:78-84 | | 3 |
| e61 | Giles TD, Robinson TD. Effects of olmesartan medoxomil on systolic blood pressure and pulse pressure in the management of hypertension. Am J Hypertens. 2004;17:690-5 | | 5 |
| e62 | Teeuw AH, Leenen FH, Geyskes GG, Boer P. Atenolol and chlorthalidone on blood pressure, heart rate, and plasma renin activity in hypertension. Clin Pharmacol Ther. 1979;25:294-302 | | 5 |
| e63 | Okeahialam BN, Thacher TD, Ibrahim TM, Anjorin FI. Lacidipine in the treatment of hypertension in black African people: antihypertensive, biochemical and haematological effects. Curr Med Res Opin. 2000;16:184-9 | | 3 |
| e64 | Os I, Bratland B, Dahløf B, Gisholt K, Syvertsen JO, Tretli S. Lisinopril or nifedipine in essential hypertension? A Norwegian multicenter study on efficacy, tolerability and quality of life in 828 patients. J Hypertens. 1991;9:1097-104 | | 3 |
| e65 | Govind U, Munro BF, Robertson LI. A fixed combination of metoprolol and chlorthalidone in hypertension. A clinical trial in general practice. S Afr Med J. 1981;60:921-4 | | 1 |
| e66 | Bossini A, Di Veroli C, Cavallotti G, Cagli V. Felodipine ER formulation in the treatment of mild hypertension: efficacy and tolerability vs placebo. Br J Clin Pharmacol. 1990;30:567-71 | | 1 |
| e67 | Campbell LM, Cowen KJ, Cranfield FR, Goves JR, Jones DF, Lees CT, Richardson PD, Teal JA, Timerick SJ. Felodipine-ER once daily as monotherapy in hypertension. J Cardiovasc Pharmacol. 1990;15:569-73 | | 1 |
| e68 | Carr AA, Bottini PB, Prisant LM, Fisher LD, Devane JG, O'Brien DE, Rhoades RB. Once-daily verapamil in the treatment of mild-to-moderate hypertension: a double-blind placebo-controlled dose-ranging study. J Clin Pharmacol. 1991;31:144-50 | | 1 |
| e69 | Chalmers JP, Wing LM, Grygiel JJ, West MJ, Graham JR, Bune AJ. Effects of once daily indapamide and pindolol on blood pressure, plasma aldosterone concentration and plasma renin activity in a general practice setting. Eur J Clin Pharmacol. 1982;22:191-6 | | 1 |
| e70 | Biston P, Mélot C, Degaute JP, Clement D, Quoidbach A. Prolonged antihypertensive effect of amlodipine: a prospective double-blind randomized study. Blood Press. 1999;8:43-8 | | 1 |
| e71 | Bainbridge AD, Macfadyen RJ, Stark S, Lees KR, Reid JL. The antihypertensive efficacy and tolerability of a low dose combination of ramipril and felodipine ER in mild to moderate essential hypertension. Br J Clin Pharmacol. 1993;36:323-30 | | 1 |
| e72 | Chan TY, Woo KS, Nicholls MG. The application of nebivolol in essential hypertension: a double-blind, randomized, placebo-controlled study. Int J Cardiol. 1992 ;35:387-95 | | 1, 2 |
| e73 | Fritschka E, Distler A, Gotzen R, Thiede HM, Philipp T. Crossover comparison of nitrendipine with propranolol in patients with essential hypertension. J Cardiovasc Pharmacol. 1984;6 Suppl 7:S1100-4 | | 1 |
| e74 | Gennari C, Nami R, Pavese G, Gragnani S, Bianchini C, Buracchi P. Calcium-channel blockade (nitrendipine) in combination with ACE inhibition (captopril) in the treatment of mild to moderate hypertension. Cardiovasc Drugs Ther. 1989;3 Suppl 1:319-25 | | 1 |
| e75 | Giorgi G, Legramante JM, Fioravanti G, Paies G, Legramante A. A comparative study of doxazosin versus atenolol in mild-to-moderate hypertension. Am Heart J. 1988;116:1801-5 | | 1 |
| e76 | Giles TD, Sander GE, Roffidal LC, Thomas MG, Given MB, Quiroz AC. Comparison of nitrendipine and hydrochlorothiazide for systemic hypertension.. Am J Cardiol. 1987;60:103-6 | | 1 |
| e77 | Hansson L, Berglund G, Andersson O, Holm M. Controlled trial of acebutolol in hypertension. Eur J Clin Pharmacol. 197714;12:89-92 | | 1 |
| e78 | Grandinetti O, Feraco E. Middle term evaluation of amlodipine vs nitrendipine: efficacy, safety and metabolic effects in elderly hypertensive patients. Clin Exp Hypertens. 1993;15 Suppl 1:197-210 | | 1 |
| e79 | Ames R, Griffing G, Marbury T, Miller E, Schoenberger J, Glenn B, Benn V, Wilkinson D. Effectiveness of indapamide versus enalapril as second-step therapy of systemic hypertension. Am J Cardiol. 1992;69:267-70 | | 1 |
| e80 | Lijnen P, Fagard R, Staessen J, Amery A. Antihypertensive effect of doxazosin and atenolol in short- and long-term double-blind comparison.. Methods Find Exp Clin Pharmacol. 1990 ;12:563-73 | | 1 |
| e81 | McCormack PM, Latham AN, Mee F, Atkins N, O'Brien ET, O'Malley K. The efficacy and duration of action of sustained-release verapamil in essential hypertension. J Cardiovasc Pharmacol. 1989;13 Suppl 4:S34-7 | | 1 |
| e82 | Nash DT, Schonfeld G, Reeves RL, Black H, Weidler DJ. A double-blind parallel trial to assess the efficacy of doxazosin, atenolol and placebo in patients with mild to moderate systemic hypertension. Am J Cardiol. 1987;59:87G-90G | | 1 |
| e83 | Honoré P. Bisoprolol versus hydrochlorothiazide plus amiloride in essential hypertension, a randomized double-blind study. Eur Heart J. 1987;8 Suppl M:95-102 | | 1 |
| e84 | McPhillips JJ, Schwemer GT, Scott DI, Zinny M, Patterson D. Effects of carvedilol on blood pressure in patients with mild to moderate hypertension. A dose response study. Drugs. 1988;36 Suppl 6:82-91 | | 1 |
| e85 | Curry RC Jr, Schwartz KM, Urban PL. Atenolol and chlorthalidone therapy for hypertension: a double-blind comparison. South Med J. 1988;81:1401-6, 1411 | | 1 |
| e86 | Bracchetti D, Gradnik R, Alberti A, Brunelli A, Orselli L, Leonardi G, Pavesi PC, Cantelli I. A double-blind comparison of bisoprolol and captopril for treatment of essential hypertension in the elderly. Cardiovasc Drugs Ther. 1990;4:261-4 | | 1 |
| e87 | Ferguson RK, Vlasses PH, Irvin JD, Swanson BN, Lee RB. A comparative pilot study of enalapril, a new converting enzyme inhibitor, and hydrochlorothiazide in essential hypertension. J Clin Pharmacol. 1982;22:281-9 | | 1 |
| e88 | Glasser SP, Chrysant SG, Graves J, Rofman B, Koehn DK. Safety and efficacy of amlodipine added to hydrochlorothiazide therapy in essential hypertension. Am J Hypertens. 1989;2:154-7 | | 2 |
| e89 | Ernst ME, Carter BL, Zheng S, Grimm RH Jr. Meta-analysis of dose-response characteristics of hydrochlorothiazide and chlorthalidone: effects on systolic blood pressure and potassium. Am J Hypertens. 2010;23:440-6 | | 1, 5 |
| e90 | Martina B, Weinbacher M, Drewe J, Gasser P. Effects of losartan titrated to losartan/hydrochlorothiazide and amlodipine on blood pressure and peripheral capillary microcirculation in patients with mild-to-moderate hypertension. J Hum Hypertens. 1998;12:473-8 | | 1 |
| e91 | Lijnen P, Fagard R, Staessen J, Amery A. Short-term double-blind comparison of doxazosin and atenolol in patients with mild to moderate hypertension. J Cardiovasc Pharmacol. 1988;12:461-6 | | 1 |
| e92 | Nechwatal W, Berger J, Blumrich W, Bouzo H, Brandl K, Braun S, Laukaitis A, Müller G, Ryba W, Schreiegg J. A double-blind comparative study of doxazosin and nitrendipine in patients with mild-to-moderate essential hypertension. Am Heart J. 1988;116:1806-14 | | 1 |
| e93 | Gostick NK, Mayhew SR, Mukerji D, Bradley I, Ganvir P, Shepherd F, Davis A, MacKay D, Hopwood AM. A randomised comparative trial of nicardipine versus amiloride and hydrochlorothiazide in mild to moderate hypertension. A report from the General Practitioner Hypertension Study Group. J Hum Hypertens. 1989;3:141-4 | | 1 |
| e94 | M A Martin, C A Phillips, and A J Smith. Acebutolol in hypertension--double-blind trial against placebo. Br J Clin Pharmacol.1978; 6: 351–356 | | 1 |
| e95 | Nievel JG, Harvard CW. Assessment of the efficacy and acceptability of an acebutolol/hydrochlorothiazide combination in the treatment of mild to moderate essential hypertension. Curr Med Res Opin. 1981;7:526-35 | | 1 |
| e96 | Grell GA, Forrester TE, Alleyne GA. Comparison of the effectiveness of a beta blocker (atenolol) and a diuretic (chlorthalidone) in black hypertensive patients. South Med J. 1984;77:1524-9 | | 1 |
| e97 | Costa FV, Borghi C, Mussi A, Ambrosioni E. Antihypertensive efficacy of two low dosages of hydrochlorothiazide in patients treated with captopril. Clin Ther. 1985;6:717–724 | | 1 |
| e98 | Mohler ER, Herrington D, Ouyang P, Mangano C, Ritter S, Davis P, Purkayastha D, Gatlin M, Vogel RA; EXPLORE Investigators. A randomized, double-blind trial comparing the effects of amlodipine besylate/benazepril HCl vs amlodipine on endothelial function and blood pressure. J Clin Hypertens (Greenwich). 2006;8:692-8 | | 1 |
| e99 | M. A. Baez, D. C. Garg, N. S. Jallad, and D. J. Weidler. Antihypertensive effect of doxazosin in hypertensive patients. Br J Clin Pharmacol. 1986; 21(Suppl 1): 63S–67S | | 1 |
| e100 | Mörlin C, Baglivo H, Boeijinga JK, Breckenridge AM, Clement D, Johnston GD, Klein W, Kramer R, Luccioni R, Meurer KA, et al. Comparative trial of lisinopril and nifedipine in mild to severe essential hypertension. J Cardiovasc Pharmacol. 1987;9 Suppl 3:S49-52 | | 1 |
| e101 | Canter D, Frank GJ, Knapp LE, Phelps M, Quade M, Texter M. Quinapril and hydrochlorothiazide combination for control of hypertension: assessment by factorial design. J Hum Hypertens. 1994;8:155-62 | | 1 |
| e102 | Hartmann A, Schwietzer G, Stratmann D, Kaltenbach M, Kober G. Effects of nitrendipine and lisinopril on blood pressure and sodium excretion in ciclosporin-associated hypertension after heart transplantation. Cardiology. 1993;83:141-9 | | 1 |
| e103 | Ajayi AA, Akintomide AO. The efficacy and tolerability of amlodipine and hydrochlorothiazide in Nigerians with essential hypertension. J Natl Med Assoc. 1995;87:485-8 | | 1 |
| e104 | W. Hart and R. J. Clarke ACE inhibition versus calcium antagonism in the treatment of mild to moderate hypertension: a multicentre study. Postgrad Med J.1993; 69: 450–455 | | 1 |
| e105 | Goldberg AI, Dunlay MC, Sweet CS. Safety and tolerability of losartan potassium, an angiotensin II receptor antagonist, compared with hydrochlorothiazide, atenolol, felodipine ER, and angiotensin-converting enzyme inhibitors for the treatment of systemic hypertension. Am J Cardiol. 1995;75:793-5 | | 1 |
| e106 | Boulet AP, Chockalingam A, Fodor JG, Laplante L, Houde M, Lenis J, Logan AG, Robitaille MN, Ryan D, Spénard J. Treatment of mild-to-moderate hypertension: comparison between a calcium-channel blocker and a potassium-sparing diuretic. J Cardiovasc Pharmacol. 1991;18 Suppl 9:S45-50 | | 1 |
| e107 | Franz IW, Agrawal B, Wiewel D, Ketelhut R. Comparison of the antihypertensive effects of carvedilol and metoprolol on resting and exercise blood pressure Clin Investig. 1992;70 Suppl 1:S53-7 | | 1 |
| e108 | Mehta JL, Lopez LM, Vlachakis ND, Gradman AH, Nash DT, O'Connell MT, Garland WT, Pickering BI. Double-blind evaluation of the dose-response relationship of amlodipine in essential hypertension. Am Heart J. 1993;125:1704-10 | | 1 |
| e109 | Bell TP, DeQuattro V, Lasseter KC, Ruff D, Hardison JD, Cushing D, Kezer AE, Michelson EL. Effective dose range of candesartan cilexetil for systemic hypertension. Candesartan Cilexetil Study Investigators. Am J Cardiol. 1999;83:272-5, A6 | | 1 |
| e110 | Bursztyn M, Gavras I, Gourley L, DeSilva J, Whalen J, Gavras H. Effect of combination therapy with atenolol and the angiotensin-converting enzyme inhibitor benazepril. Clin Ther. 1994;16:429-36 | | 1 |
| e111 | Franz IW, Behr U, Ketelhut R. Resting and exercise blood pressure with atenolol, enalapril and a low-dose combination. J Hypertens Suppl. 1987;5:S37-41 | | 1 |
| e112 | Moser M, Abraham PA, Bennett WM, Brachfeld N, Goodman RP, McKenney JM, Hollifield JW, Kirkendall WM, Lasseter KC, Leon AS, et al. The effects of benazepril, a new angiotensin-converting enzyme inhibitor, in mild to moderate essential hypertension: a multicenter study. Clin Pharmacol Ther. 1991;49:322-9 | | 1 |
| e113 | Gordon RD, Klemm SA, Tunny TJ, Wicks JR, Elmfeldt DB. Effects of felodipine, metoprolol and their combination on blood pressure at rest and during exercise and on volume regulatory hormones in hypertensive patients. Blood Press. 1995;4:300-6 | | 1 |
| e114 | Frishman WH, Kirkendall W, Lunn J, McCarron D, Moser M, Schnaper H, Smith LK, Sowers J, Swartz S, Zawada E. Diuretics versus calcium-channel blockers in systemic hypertension: a preliminary multicenter experience with hydrochlorothiazide and sustained-release diltiazem. Am J Cardiol. 1985;56:92H-96H | | 1 |
| e115 | Smith TR, Philipp T, Vaisse B, Bakris GL, Wernsing M, Yen J, Glazer R. Amlodipine and valsartan combined and as monotherapy in stage 2, elderly, and black hypertensive patients: subgroup analyses of 2 randomized, placebo-controlled studies. J Clin Hypertens (Greenwich). 2007;9:355-64 | | 5, 2 |
| e116 | Coope J, Warrender TS. Randomised trial of treatment of hypertension in elderly patients in primary care. Br Med J (Clin Res Ed). 1986;293:1145-51 | | 2, 4 |
| e117 | ALLHAT Officers and Coordinators for the ALLHAT Collaborative Research Group. The Antihypertensive and Lipid-Lowering Treatment to Prevent Heart Attack Trial. Major outcomes in high-risk hypertensive patients randomized to angiotensin-converting enzyme inhibitor or calcium channel blocker vs diuretic: The Antihypertensive and Lipid-Lowering Treatment to Prevent Heart Attack Trial (ALLHAT). JAMA. 2002;288:2981-97 | | 2, 4 |
| e118 | Bosch J, Yusuf S, Pogue J, Sleight P, Lonn E, Rangoonwala B, Davies R, Ostergren J, Probstfield J; HOPE Investigators. Heart outcomes prevention evaluation.Use of ramipril in preventing stroke: double blind randomised trial BMJ. 2002;324:699-702 | | 2, 4 |
| e119 | Stumpe KO, Ludwig M. Antihypertensive efficacy of olmesartan compared with other antihypertensive drugs. J Hum Hypertens. 2002;16 Suppl 2:S24-8 | | 2, 4 |
| e120 | Oparil S. Comparative antihypertensive efficacy of olmesartan: comparison with other angiotensin II receptor antagonists. J Hum Hypertens. 2002;16 Suppl 2:S17-23 | | 2 |
| e121 | Frishman WH, Brobyn R, Brown RD, Johnson BF, Reeves RL, Wombolt DG. Amlodipine versus atenolol in essential hypertension. Am J Cardiol. 1994;73:50A-54A | | 1 |
| e122 | Brunner HR, Bidiville J, Waeber G, Porchet M, Nussberger J, Waeber | | 1 |
| e123 | Fagher B, Katzman P, Hulthén UL, Henningsen NC, Thulin T. Antihypertensive efficacy and tolerability of enalapril and slow-release verapamil in essential hypertension: a double-blind, cross-over study. J Intern Med. 1991;230:219-26 | | 1 |
| e124 | Wu SC, Liu CP, Chiang HT, Lin SL. Prospective and randomized study of the antihypertensive effect and tolerability of three antihypertensive agents, losartan, amlodipine, and lisinopril, in hypertensive patients. Heart Vessels. 2004;19:13-8 | | 1 |
| e125 | Webster J, Robb OJ, Witte K, Petrie JC. Single doses of enalapril and atenolol in hypertensive patients treated with bendrofluazid J Hypertens. 1987;5:457-60 | | 1 |
| e126 | Webster J, Petrie JC, Robb OJ, Trafford J, Burgess J, Richardson PJ, Davidson C, Fairhurst G, Vandenburg MJ, Cooper WD, et al. Enalapril in moderate to severe hypertension .A comparison with atenolol Br J Clin Pharmacol. 1986;21:489-95 | | 1 |
| e127 | Pandhi P, Sahai J, Wahi PL, Sharma BK, Sharma PL. Double-blind, randomized, cross-over clinical trial of metoprolol and propranolol in mild to moderate essential hypertension. Int J Clin Pharmacol Ther Toxicol. 1987;25:163-5 | | 1 |
| e128 | Wessels F. Double-blind comparison of doxazosin and enalapril in patients with mild or moderate essential hypertension. [Am Heart J.](http://www.ncbi.nlm.nih.gov/pubmed/1824653) 1991 Jan;121(1 Pt 2):299-303 | | 4 |
| e129 | Lucas C, Jenkins P, Mendels J, Due D, Forbes WP, Sirgo MA. The effectiveness of labetalol compared to hydrochlorothiazide in hypertensive black patients. J Natl Med Assoc. 1991 ;83:866-71 | | 1 |
| e130 | Eber B, Brussee H, Rotman B, Kramer R, Klein W. Evaluation of the antihypertensive effect of lisinopril compared with nifedipine in patients with mild to severe essential hypertension. Angiology. 1992;43:482-9 | | 1 |
| e131 | Chao CL, Lin YH, Lin LC, Lin LY, Tsai CT, Wang YC, Hwang JJ, Chen JC, Chiang FT. Efficacy and safety of valsartan/hydrochlorothiazide fixed-dose combination compared with amlodipine monotherapy as first-line therapy for mild to moderate hypertension. J Int Med Res. 2009;37:289-97 | | 1, 2 |
| e132 | Minami J, Furukata S, Ishimitsu T, Matsuoka H. Comparison of therapies between fixed-dose telmisartan/hydrochlorothiazide and losartan/hydrochlorothiazide in patients with mild to moderate hypertension. Int Heart J. 2009;50:85-93 | | 2 |
| e133 | Maclean D, Mitchell ET, Coulson RR, Fitzsimons TJ, McDevitt DG. Atenolol-nifedipine combinations compared to atenolol alone in hypertension: efficacy and tolerability. Br J Clin Pharmacol. 1988 ;25:425-31 | | 1 |
| e134 | Costa FV, Borghi C, Ambrosioni E. Captopril and oxprenolol in a fixed combination with thiazide diuretics: comparison of their antihypertensive efficacy and metabolic effects. Clin Ther. 1984;6(5):708-18 | | 1 |
| e135 | McGill JB, Reilly PA. Combination treatment with telmisartan and hydrochlorothiazide in black patients with mild to moderate hypertension. Clin Cardiol. 200;24:66-72 | | 1 |
| e136 | Gums JG, Lopez LM, Quay GP, Stein GH, McCarley DL. Comparative evaluation of enalapril and hydrochlorothiazide in elderly patients with mild to moderate hypertension. Drug Intell Clin Pharm. 1988 ;22:680- | | 1 |
| e137 | Hollifield JW, Heusner JJ, DesChamps M, Gray J, Spyker DA, Peace KE, Dickson B. Comparison of equal-weight oral dosages of verapamil hydrochloride and diltiazem hydrochloride in patients with mild to moderate hypertension. Clin Pharm. 1988;7:129-34 | | 1 |
| e138 | Rumboldt Z, Marinković M, Drinovec J. Enalapril versus captopril: a double-blind multicentre comparison in essential hypertension. Int J Clin Pharmacol Res. 1988;8:181-8 | | 1 |
| e139 | Pascoe J, Moon R, Gray D, Musgrove J. Enalapril maleate and atenolol combined with hydrochlorothiazide in moderate to severe essential hypertension. N Z Med J. 1985;98:951-3 | | 1 |
| e140 | Salako BL, Kadiri S, Walker O, Fehintola FA. Evaluation of lacidipine (a calcium blocker) in the treatment of hypertension in black African people: a double-blind comparison with hydrochlorothiazide. Afr J Med Med Sci. 1998;27:73-5 | | 1 |
| e141 | Shimosawa T, Gohchi K, Yatomi Y, Fujita T. Effectiveness of add-on low-dose diuretics in combination therapy for hypertension: losartan/hydrochlorothiazide vs. candesartan/amlodipine. Hypertens Res. 2007 ;30:831-7 | | 1 |
| e142 | Velasco M, Guevara J, Morillo J, Ramírez A, Urbina-Quintana A, Hernández-Pieretti O. Antihypertensive effect of atenolol alone or combined with chlorthalidone in patients with essential hypertension.Br J Clin Pharmacol. 1980 ;9:499-504 | | 1 |
| e143 | Taylor SH, Lee PS, Sharma SK. A comparison of doxazosin and enalapril in the treatment of mild and moderate essential hypertension. Am Heart J. 1988;116:1820-5 | | 1 |
| e144 | Rutan GH, Feig PU, May S, Kriegman AG, Brady EM. A comparison of once-daily atenolol and metoprolol using office and ambulatory blood pressure monitoring. J Clin Pharmacol. 1993;33:418-26 | | 1 |
| e145 | Velasco M, Urbina A, Silva H, Fonseca R, Guevara J, Hernandez R, Pieretti OH. A double-blind, parallel, comparative evaluation of amlodipine vs. captopril in the monotherapeutic treatment of mild and moderate essential hypertension. J Cardiovasc Pharmacol. 1991;17 Suppl 1:S19-21 | | 1 |
| e146 | Webster J, Jeffers TA, Galloway DB, Petrie JC, Barker NP. Atenolol, methyldopa, and chlorthalidone in moderate hypertension. Br Med J. 1977;1:76-8 | | 1 |
| e147 | Zanchetti A, Omboni S. Comparison of candesartan versus enalapril in essential hypertension. Italian Candesartan Study Group. Am J Hypertens. 2001;14:129-34 | | 1 |
| e148 | Yutaka M, Mifune M, Kubota E, Itoh H, Saito I. Comparison of effects of low dose of spironolactone and a thiazide diuretic in patients with hypertension treated with an angiotensin-converting enzyme inhibitor or an angiotensin type 1 receptor blocker. [Clin Exp HypertensHYPERLINK "http://www.ncbi.nlm.nih.gov/pubmed/20001457".](http://www.ncbi.nlm.nih.gov/pubmed/20001457) 2009 Nov;31(8):648-56 | | 1, 2 |
| e149 | Hua AS, Kalowski S, Whitworth JA, Kincaid-Smith P. Hydrallazine with beta blockade and diuretic in the treatment of hypertension. Med J Aust. 1980;1:226-8 | | 1 |
| e150 | Morley CA, Cavalcanti C, Perrins EJ, Sutton R. A comparison of once daily atenolol and metoprolol SA in mild to moderate hypertension. Br J Clin Pharmacol. 1983;15:715-7 | | 1 |
| e151 | Lacourcière Y, Poirier L, Provencher P. Comparison of the effects of amlodipine and captopril on clinic and ambulatory blood pressure. J. Hum Hypertens. 1992;6 Suppl 1:S25-8 | | 1 |
| e152 | Kuo SW, Pei-Dee, Hung YJ, Hsieh AT, Wu LY, Hsieh CH, He CT, Yang TC, Lian WC. Effect of indapamide SR in the treatment of hypertensive patients with type 2 diabetes. Am J Hypertens. 2003;16:623-8 | | 1 |
| e153 | Salako LA, Falase AO, Aderounmu AF, Walker O. Assessment of a fixed-dosage combination of atenolol and chlorthalidone (Tenoretic) in hypertensive Nigerians. Afr J Med Med Sci. 1990;19:57-61 | | 1 |
| e154 | Burgess ED, Lacourcière Y, Ruilope-Urioste LM, Oparil S, Kleiman JH, Krause S, Roniker B, Maurath C. Long-term safety and efficacy of the selective aldosterone blocker eplerenone in patients with essential hypertension. Clin Ther. 2003;25:2388-404 | | 1 |
| e155 | Young PH. A comparison of carvedilol with atenolol in the treatment of mild-to-moderate essential hypertension. J Cardiovasc Pharmacol. 1992;19 Suppl 1:S82-5 | | 1 |
| e156 | Weir MR, Lavin PT. Comparison of the efficacy and tolerability of lisinopril and sustained-release verapamil in older patients with hypertension . Clin Ther. 1991;13:401-8 | | 1 |
| e157 | Rumboldt Z, Simunić M, Bagatin J, Rumboldt M, Marinković M, Janezić A. Controlled multicentre comparison of captopril versus lisinopril in the treatment of mild-to-moderate arterial hypertension. Int J Clin Pharmacol Res. 1993;13:35-41 | | 1 |
| e158 | Webster J1, Robb OJ, Jeffers TA, Scott AK, Petrie JC. Once daily amlodipine in the treatment of mild to moderate hypertension. J Cardiovasc Pharmacol. 1988;12 Suppl 7:S72-5 | | 1 |
| e159 | Watts RW, Dufek A, Wing LM. Comparison of felodipine and enalapril monotherapy in essential hypertension. Blood Press. 1993;2:53-8 | | 1 |
| e160 | Okanga JB. Atenolol (Tenormin) compared with methyldopa (Aldomet) in the treatment of hypertension. East Afr Med J. 1978;55:447-52 | | 1 |
| e161 | Scott A K , Rigby J W , Webster J , Hawksworth G M, Petrie J C , and Lovell H G Atenolol and metoprolol once daily in hypertension. Br Med J (Clin Res Ed).1982; 284: 1514-1516 | | 1 |
| e162 | Leonetti G, Emeriau JP, Knauf H, Pujadas JO, Calvo-Gomez C, Abate G; European Study Investigators.82; 284: 1514-1516. Evaluation of long-term efficacy and acceptability of indapamide SR in elderly hypertensive patients. Curr Med Res Opin. 2005;21:37-46 | | 5 |
| e163 | Julius S, Kjeldsen SE, Weber M, Brunner HR, Ekman S, Hansson L, Hua T, Laragh J, McInnes GT, Mitchell L, Plat F, Schork A, Smith B, Zanchetti A; VALUE trial group. Outcomes in hypertensive patients at high cardiovascular risk treated with regimens based on valsartan or amlodipine: the VALUE randomised trial. Lancet. 2004;363:2022-31 | | 2, 4 |
| e164 | Bauer JH, Jones LB. Comparative studies: enalapril versus hydrochlorothiazide as first-step therapy for the treatment of primary hypertension . Am J Kidney Dis. 1984;4:55-62 | | 1 |
| e165 | Schweizer J, Ulmer HJ, Benduhn H, Klebs S. Efficacy and tolerability of aliskiren 300  mg/hydrochlorothiazide 25 mg (± amlodipine 5 mg) in hypertensive patients not controlled by candesartan 32 mg plus HCT 25mg. Curr Med Res Opin. 2011;27:131-40 | | 5 |
| e166 | Videbaek LM, Jacobsen IA. Crossover comparison of the pharmacokinetics of amlodipine and felodipine ER in hypertensive patients. Int J Clin Pharmacol Ther. 1997;35:514-8 | | 5, 1 |
| e167 | Ohman KP, Karlberg BE. Enalapril and atenolol in primary hypertension--a comparative study of blood pressure lowering and hormonal effects. Scand J Urol Nephrol Suppl. 1984;79:93-7 | | 1 |
| e168 | Jamerson KA, Nwose O, Jean-Louis L, Schofield L, Purkayastha D, Baron M. Initial angiotensin-converting enzyme inhibitor/calcium channel blocker combination therapy achieves superior blood pressure control compared with calcium channel blocker monotherapy in patients with stage 2 hypertension. Am J Hypertens. 2004;17:495-501 | | 5, 3 |
| e169 | Lai WT, Park JE, Dongre N, Wang J. Efficacy, safety, and tolerability of valsartan/hydrochlorothiazide in Asian patients with essential hypertension. Adv Ther. 201;28:427-38 | | 5, 2 |
| e170 | Menne J, Farsang C, Deák L, Klebs S, Meier M, Handrock R, Sieder C, Haller H. Valsartan in combination with lisinopril versus the respective high dose monotherapies in hypertensive patients with microalbuminuria: the VALERIA trial. J Hypertens. 2008;26:1860-7 | | 1 |
| e171 | Aberg H, Frithz G, Mörlin C. Comparison of captopril (SQ 14225) with hydrochlorothiazide in the treatment of essential hypertension. Int J Clin Pharmacol Ther Toxicol. 1981;19:368-71 | | 1 |
| e172 | Guul SJ, Os I, Jounela AJ. The efficacy and tolerability of enalapril in a formulation with a very low dose of hydrochlorothiazide in hypertensive patients resistent to enalapril monotherapy. Am J Hypertens. 1995 ;8:727-31 | | 3 |
| e173 | Ferguson RK, Vlasses PH, Irvin JD, Swanson BN, Lee RB. A comparative pilot study of enalapril, a new converting enzyme inhibitor, and hydrochlorothiazide in essential hypertension. J Clin Pharmacol. 1982;22:281-9 | | 1 |
| e174 | Fujiwara T, Ii Y, Hatsuzawa J, Murase H, Watanabe T, Murakami M, Kimura N, Buch J, Tsuchihashi T, Saruta T. The Phase III, double-blind, parallel-group controlled study of amlodipine 10 mg once daily in Japanese patients with essential hypertension who insufficiently responded to amlodipine 5 mg once daily. J Hum Hypertens. 2009;23:521-9 | | 2 |
| e175 | Abengowe CU. A double-blind comparison of acebutolol (Sectral) and propranolol (Inderal) in the treatment of hypertension in black Nigerian patients. J Int Med Res. 1985;13:116-21 | | 1, 2 |
| e176 | Davies J, Jensen H, Garsdal P. A double-blind comparison of amlodipine and placebo added to open enalapril in patients with moderate to severe essential hypertension. J Cardiovasc Pharmacol. 1991;17 Suppl 1:S16-8 | | 1 |
| e177 | Dixon MS, Thomas P, Sheridan DJ. A randomized double-blind study of bisoprolol versus atenolol in mild to moderate essential hypertension. Eur J Clin Pharmacol. 1990;38:21-4 | | 1, 3 |
| e178 | de Planque BA. A double-blind comparative study of doxazosin and prazosin when administered with beta-blockers or diuretics. Am Heart J. 1991;121:304-11 | | 1 |
| e179 | Cappuccio FP, Markandu ND, Singer DR, Buckley MG, Miller MA, Sagnella GA, MacGregor GA. A double-blind crossover study of the effect of concomitant diuretic therapy in hypertensive patients treated with amlodipine. Am J Hypertens. 1991;4:297-302 | | 1 |
| e180 | Fagard R, Lijnen P, Pardaens K, Thijs L, Vinck W. A randomised, placebo-controlled, double-blind, crossover study of losartan and enalapril in patients with essential hypertension. J Hum Hypertens. 2001;15:161-7 | | 1 |
| e181 | Andrén L, Svensson A, Hansson L. Captopril or atenolol in essential hypertension. Acta Med Scand Suppl. 1983;677:115-8 | | 1 |
| e182 | Boike SC, Durley Y, Cubberley RB. Atenolol and chlorthalidone administered alone and in combination for essential hypertension. Clin Pharm. 1982;1:449-53 | | 3 |
| e183 | Franco RJ, Goldflus S, McQuitty M, Oigman W; Valsartan/HCTZ Combination Therapy in Brazil Study Group. Efficacy and tolerability of the combination valsartan/hydrochlorothiazide compared with amlodipine in a mild-to-moderately hypertensive Brazilian population Blood Press Suppl. 2003;2:41-7 | | 2 |
| e184 | Cappuccio FP, Markandu ND, Singer DR, Crane M, Carney C, MacGregor GA. Double-blind comparison between nifedipine and amlodipine for the treatment of essential hypertension. J Hum Hypertens. 1993;7:365-8 | | 1 |
| e185 | Durel LA, Hayashi PJ, Weidler DJ, Schneiderman N. Effectiveness of antihypertensive medications in office and ambulatory settings: a placebo-controlled comparison of atenolol, metoprolol, chlorthalidone, verapamil, and an atenolol-chlorthalidone combination J Clin Pharmacol. 1992;32:564-70 | | 1 |
| e186 | Chrysant SG, Fagan T, Glazer R, Kriegman A. Effects of benazepril and hydrochlorothiazide, given alone and in low- and high-dose combinations, on blood pressure in patients with hypertension. Arch Fam Med. 1996;5:17-24 | | 3 |
| e187 | Caponnetto S, Alberti D, Bafico GL, Bertulla A, Camerieri A, Francucci BM, Gatto E, Gentile A, Livi S, Mereto PE, et al. Effects of two doses of the fixed-combination chlorthalidone and slow-release metoprolol on blood pressure at rest and during exercise: a multicenter study. Int J Clin Pharmacol Ther Toxicol. 1986;24:574-9 | | 3 |
| e188 | el Mangoush M1, Singh NK, Kumar S, Basha A, Gupta BS, Bolya YK, Gamati A. Efficacy of enalapril in essential hypertension and its comparison with atenolol. Postgrad Med J. 1990;66:446-9 | | 3 |
| e189 | Fogari R, Zoppi A, Mugellini A, Preti P, Perrone T, Maffioli P, Derosa G. Effects of valsartan versus olmesartan addition to amlodipine/hydrochlorothiazide combination in treating stage 2 hypertensive patients. Expert Opin Pharmacother. 2012;13:629-36 | | 3 |
| e190 | Bakris G, Sica D, Ram V, Fagan T, Vaitkus PT, Anders RJ. A comparative trial of controlled-onset, extended-release verapamil, enalapril, and losartan on blood pressure and heart rate changes. Am J Hypertens. 2002;15:53-7 | | 3 |
| e191 | Chrysant SG, Bakris GL. Amlodipine/benazepril combination therapy for hypertensive patients nonresponsive to benazepril monotherapy. Am J Hypertens. 2004;17:590-6 | | 3 |
| e192 | Bönner G; Multicentre Study Group. Antihypertensive efficacy and tolerability of candesartan-hydrochlorothiazide 32/12.5 mg and 32/25 mg in patients not optimally controlled with candesartan monotherapy. Blood Press Suppl. 2008;2:22-30 | | 3 |
| e193 | Destro M, Crikelair N, Yen J, Glazer R. Triple combination therapy with amlodipine, valsartan, and hydrochlorothiazide vs dual combination therapy with amlodipine and hydrochlorothiazide for stage 2 hypertensive patients. Vasc Health Risk Manag. 2010;6:821-7 | | 3 |
| e194 | Glasser SP, Neutel JM, Gana TJ, Albert KS. Efficacy and safety of a once daily graded-release diltiazem formulation in essential hypertension. Am J Hypertens. 2003 ;16:51-8 | | 4 |
| e195 | DeQuattro V, Lee D, Messerli F. Efficacy of combination therapy with trandolapril and verapamil sr in primary hypertension: a 4 x 4 trial design. Clin Exp Hypertens. 1997;19:373-87 | | 5, 3 |
| e196 | Elliott WJ, Montoro R, Smith D, Leibowitz M, Hwang C, Gradman AH, Schleman M, Klibaner M. Comparison of two strategies for intensifying antihypertensive treatment: low-dose combination (enalapril + felodipine ER) versus increased dose of monotherapy (enalapril). Am J Hypertens. 1999;12:691-6 | | 3 |
| e197 | Bahena JH, Estrella ME, Muñoz M. Quinapril versus atenolol in the treatment of mild to moderate essential hypertension. Clin Ther. 1992;14:527-36 | | 2 |
| e198 | Davidov M. Acebutolol in essential hypertension: results of two multicenter studies against placebo and propranolol. Am Heart J. 1985;109:1158-67 | | 3 |
| e199 | Bochsler JA, Simmons RL, Ward PJ, Chester PC, Latham AN. Verapamil SR and propranolol LA: a comparison of efficacy and side effects in the treatment of mild to moderate hypertension. J Hum Hypertens. 1988;1:305-10. | | 1, 3 |
| e200 | Bühler FR, Berglund G, Anderson OK, Brunner HR, Scherrer U, van Brummelen P, Distler A, Philipp T, Fogari R, Mimran A, et al. Double-blind comparison of the cardioselective beta-blockers bisoprolol and atenolol in hypertension: the Bisoprolol International Multicenter Study (BIMS). J Cardiovasc Pharmacol. 1986;8 Suppl 11:S122-7 | | 6 |
| e201 | Schmieder RE, Philipp T, Guerediaga J, Gorostidi M, Bush C, Keefe DL. Aliskiren-based therapy lowers blood pressure more effectively than hydrochlorothiazide-based therapy in obese patients with hypertension: sub-analysis of a 52-week, randomized, double-blind trial. J Hypertens. 2009;27:1493-501 | | 6 |
| e202 | Ferguson RK, Vlasses PH, Irvin JD, Swanson BN, Lee RB. A comparative pilot study of enalapril, a new converting enzyme inhibitor, and hydrochlorothiazide in essential hypertension. J Clin Pharmacol. 1982l;22:281-9 | | 1 |
| e203 | Holzgreve H, Distler A, Michaelis J, Philipp T, Wellek S. Hydrochlorothiazide and verapamil in the treatment of hypertension. J Cardiovasc Pharmacol. 1991;18 Suppl 6:S33-7 | | 3 |
| e204 | Holzgreve H.; Distler A.; Michaelis J.; Philipp T.; Wellek S. Verapamil versus hydrochlorothiazide in the treatment of hypertension: results of long term double blind comparative trial. BMJ.1989; 299: 881–886 | | 3 |
| e205 | Byyny RL, LoVerde M, Mitchell W. Treatment of hypertension in the elderly with a new calcium channel blocking drug, nitrendipine. Am J Med. 1989;86:49-55 | | 1 |
| e206 | Cappuccio FP, Markandu ND, Singer DR, MacGregor GA. Amlodipine and lisinopril in combination for the treatment of essential hypertension: efficacy and predictors of response. J Hypertens. 1993;11:839-47 | | 1 |
| e207 | Abson CP, Levy LM, Eyherabide G. Once-daily atenolol in hypertensive Zimbabwean blacks. A double-blind trial using two different doses. S Afr Med J. 1981;60:47-8 | | 1 |
| e208 | Cappuccio FP, Markandu ND, Tucker FA, Shore AC, MacGregor GA. A double-blind study of the blood pressure lowering effect of a thiazide diuretic in hypertensive patients already on nifedipine and a beta-blocker. J Hypertens. 1987;5:733-8 | | 1 |
| e209 | Bracchetti D, Gradnik R, Alberti A, Brunelli A, Orselli L, Leonardi G, Pavesi PC, Cantelli I. A double-blind comparison of bisoprolol and captopril for treatment of essential hypertension in the elderly. Cardiovasc Drugs Ther. 1990;4:261-4 | | 1 |
| e210 | Chen MF, Yang CY, Chen WJ, Lee CM, Wu CC, Liau CS, Lee YT. A double-blind comparison of once-daily metoprolol controlled-release and atenolol in the treatment of Chinese patients with mild to moderate hypertension. Cardiovasc Drugs Ther. 1995;9:401-6 | | 2 |
| e211 | Bueno J, Amiguet JA, Carasusan J, Cebollada J, Carretero J. Bisoprolol vs. chlorthalidone: a randomized, double-blind, comparative study in arterial hypertension. J Cardiovasc Pharmacol. 1990;16 Suppl 5:S189-92 | | 1 |
| e212 | Chatterji AN. A randomized crossover comparison of acebutolol and methyldopa in the treatment of mild to moderate essential hypertension. Curr Med Res Opin. 1978;5:675-81 | | 1 |
| e213 | Association of Black Cardiologists (ABC) Candesartan Study Group. Evaluation of candesartan cilexetil in black patients with systemic hypertension: the ABC Trial. Heart Dis. 2000;2:392-9 | | 3 |
| e214 | Bakris GL, Iyengar M, Lukas MA, Ordronneau P, Weber MA. Effect of combining extended-release carvedilol and lisinopril in hypertension: results of the COSMOS study. J Clin Hypertens (Greenwich). 2010;12:678-86 | | 3 |
| e215 | Fogari R, Mugellini A, Derosa G; CANDIA (CANdesartan and DIuretic vs. Amlodipine in hypertensive patients) Study Group. Efficacy and tolerability of candesartan cilexetil/hydrochlorothiazide and amlodipine in patients with poorly controlled mild-to-moderate essential hypertension. J Renin Angiotensin Aldosterone Syst. 2007;8:139-44 | | 2 |
| e216 | Motolese M, Muiesan G, Colombi A. Hypotensive effect of oxprenolol in mild to moderate hypertension: a multicentre controlled study. J Clin Pharmacol. 1975;8:21-31 | | 1 |
| e217 | Morgan T, Snowden R, Butcher L. Effect of carvedilol and metoprolol on blood pressure, blood flow, and vascular resistance. J Cardiovasc Pharmacol. 1987;10 Suppl 11:S124-9 | | 1 |
| e218 | De Divitiis O, Di Somma S, Petitto M, Fazio S, Ligouri V. Indapamide and atenolol in the treatment of hypertension: double-blind comparative and combination study. Curr Med Res Opin. 1983;8:493-500 | | 1 |
| e219 | Searle M, Dathan R, Dean S, Christensen CC, Westheim A. Doxazosin in combination with atenolol in essential hypertension: a double-blind placebo-controlled multicentre trial. Eur J Clin Pharmacol. 1990;39:299-300 | | 1 |
| e220 | Lacourcière Y, Poirier L, Boucher S, Spenard J. Comparative effects of diltiazem sustained-release formulation and metoprolol on ambulatory blood pressure and plasma lipoproteins. Clin Pharmacol Ther. 1990;48:318-24 | | 2 |
| e221 | Schrijver G, Weinberger MH. Hydrochlorothiazide and spironolactone in hypertension. Clin Pharmacol Ther. 1979;25:33-42 | | 3 |
| e222 | Oparil S. Candesartan cilexetil in combination with low-dose hydrochlorothiazide is effective in severe hypertension. Am J Cardiol. 1999;84:35S-41S | | 3 |
| e223 | Andrén L, Karlberg B, Ohman P, Svensson A, Asplund J, Hansson L. Captopril and atenolol combined with hydrochlorothiazide in essential hypertension. Br J Clin Pharmacol. 1982;14 Suppl 2:107S-111S | | 3 |
| e224 | The Canadian Enalapril Study Group. Comparison of monotherapy with enalapril and atenolol in mild to moderate hypertension. The Canadian Enalapril Study Group. CMAJ. 1987; 137: 803-808 | | 3 |
| e225 | Papademetriou V. Comparison of Nebivolol monotherapy versus Nebivolol in combination with other antihypertensive therapies for the treatment of hypertension. Am J Cardiol. 2009;103:273-8 | | 3 |
| e226 | Tarkiainen A, Saraste K, Seppälä T, Gordin A, Auvinen J. A controlled study of the antihypertensive effect of carteolol, a newβ-adrenergic receptor blocking drug, in combination with hydrochlorthiazide and amiloride. Eur J Clin Pharmacol. 1981;19:239-44 | | 1, 3 |
| e227 | Schmidt A, Adam SA, Kolloch R, Weidinger G, Handrock R. Antihypertensive effects of valsartan/hydrochlorothiazide combination in essential hypertension. Blood Press. 2001;10:230-7 | | 3 |
| e228 | Holland OB, Gomez-Sanchez CE, Kuhnert LV, Poindexter C, Pak CY. Antihypertensive comparison of furosemide with hydrochlorothiazide for black patients. Arch Intern Med. 1979;139:1015-21 | | 1, 3 |
| e229 | Leon AS, Hunninghake DB. A multiclinic double-blind comparison of timolol and hydrochlorothiazide alone and in combination in th e treatment of essential hypertension. J Clin Pharmacol. 1983;23:5-15 | | 3 |
| e230 | Woo J, Woo KS, Kin T, Vallance-Owen J. A single-blind, randomized, cross-over study of angiotensin-converting enzyme inhibitor and triamterene and hydrochlorothiazide in the treatment of mild to moderate hypertension in the elderly. Arch Intern Med. 1987;147:1386-9 | | 2 |
| e231 | Kochar MS, Bolek G, Kalbfleisch JH, Olzinski P. A 52-week comparison of lisinopril, hydrochlorothiazide, and their combination in hypertension. J Clin Pharmacol. 1987;27:373-7 | | 3, 1 |
| e232 | Tuomilehto J, Nissinen A. Double-blind comparison of metoprolol, alprenolol, and oxprenolol in hypertension. Eur J Clin Pharmacol. 1979;16:369-74 | | 3 |
| e233 | Pool JL, Guthrie RM, Littlejohn TW 3rd, Raskin P, Shephard AM, Weber MA, Weir MR, Wilson TW, Wright J, Kassler-Taub KB, Reeves RA. Dose-related antihypertensive effects of irbesartan in patients with mild-to-moderate hypertension. Am J Hypertens. 1998;11:462-70 | | 3 |
| e234 | Vander Elst E, Dombey SL, Lawrence J, Vlassak W. Controlled comparison of the effects of furosemide and hydrochlorothiazide added to propranolol in the treatment of hypertension. Am Heart J. 1981;102:734-40 | | 3 |
| e235 | Medical Research Council Working Party. Comparison of the antihypertensive efficacy and adverse reactions to two doses of bendrofluazide and hydrochlorothiazide and the effect of potassium supplementation on the hypotensive action of bendrofluazide: substudies of the Medical Research Council's trials of treatment of mild hypertension. J Clin Pharmacol. 1987;27:271-7 | | 6 |
| e236 | Trenkwalder P, Plaschke M, Aulehner R, Lydtin H. Felodipine or hydrochlorothiazide/triamterene for treatment of hypertension in the elderly: effects on blood pressure, hypertensive heart disease, metabolic and hormonal parameters. Blood Press. 1996;5:154-63 | | 3 |
| e237 | Sun NL, Zhu JR, Zhao Y, Tu YM; Co-Diovan Trial Investigators. Combination antihypertensive therapy with valsartan and hydrochlorothiazide in Chinese patients with mild-to-moderate hypertension. Curr Med Res Opin. 2008;24:2863-71 | | 2 |
| e238 | Wahl J, Singh BN, Thoden WR. Comparative hypotensive effects of acebutolol and hydrochlorothiazide in patients with mild to moderate essential hypertension: a double-blind multicenter evaluation. Am Heart J. 1986;111:353-62 | | 3 |
| e239 | Arriaga-Gracia J, Sánchez-Garcia JL, González-García CA. Nicardipine or propranolol combined with hydrochlorothiazide in patients with essential hypertension. Proc West Pharmacol Soc. 1993;36:39-43 | | 3 |
| e240 | Jäättelä A. Fixed combination of sotalol and hydrochlorothiazide in the treatment of uncomplicated hypertension. Eur J Clin Pharmacol. 1981;19:395-401 | | 3 |
| e241 | Dahlöf B, Hansson L, Acosta JH, Bolzano K, Fairhurst G, Ferreira C, Kaarsalo E, Silva MC, Simone A. Controlled trial of enalapril and hydrochlorothiazide in 200 hypertensive patients. Am J Hypertens. 1988 ;1:38-41 | | 3 |
| e242 | Rogstad B. A comparison of lisinopril and nifedipine in the treatment of mild to moderate hypertension. A multicentre study. Eur J Clin Pharmacol. 1994;46:487-9 | | 3 |
| e243 | The United Kingdom Lacidipine Study Group. A double-blind comparison of the efficacy and safety of lacidipine with atenolol in the treatment of essential hypertension. J Cardiovasc Pharmacol. 1991;17 Suppl 4:S27-30 | | 3 |
| e244 | Radevski IV, Valtchanova ZP, Candy GP, Hlatswayo MN, Sareli P. Antihypertensive effect of low-dose hydrochlorothiazide alone or in combination with quinapril in black patients with mild to moderate hypertension. J Clin Pharmacol. 2000;40:713-21 | | 3 |
| e245 | Poulter NR, Sanderson JE, Thompson AV, Sever PS, Chang CL. Comparison of nifedipine and propranolol as second line agent for hypertension in black Kenyans. BMJ. 1993;306:621-2 | | 6, 2 |
| e246 | Ernst ME, Carter BL, Goerdt CJ, Steffensmeier JJ, Phillips BB, Zimmerman MB, Bergus GR. Comparative antihypertensive effects of hydrochlorothiazide and chlorthalidone on ambulatory and office blood pressure. Hypertension. 2006;47:352-8 | | 1 |
| e247 | Campo C, Fernández G, González-Esteban J, Segura J, Ruilope LM; ESPADA Study Group. Comparative study of home and office blood pressure in hypertensive patients treated with enalapril/HCTZ 20/6 mg: the ESPADA study. Blood Press. 2000;9:355-62 | | 6 |
| e248 | Nilsson OR, Atterhög JH, Castenfors J, Jorfelt L, Karlberg BE, Thulin T, Tolagen K, Wettre S, Ohman KP. A comparison of 100 mg atenolol and 100 mg metoprolol once a day at rest and during exercise in hypertensives. Acta Med Scand. 1984;216:301-7 | | 3 |
| e249 | Lavenius B, Hansson L. A double-blind comparison of spironolactone and hydrochlorothiazide in hypertensive patients treated with metoprolol. Int J Clin Pharmacol Ther Toxicol. 1982;20:291-5 | | 3 |
| e250 | Saruta T, Ogihara T, Matsuoka H, Suzuki H, Toki M, Hirayama Y, Nonaka K, Takahashi K. Antihypertensive efficacy and safety of fixed-dose combination therapy with losartan plus hydrochlorothiazide in Japanese patients with essential hypertension. Hypertens Res. 2007;30:729-39 | | 2 |
| e251 | Zhu JR, Bai J, Cai NS, Tang B, Fan WH, Guo JZ, Ke YN, Guo JX, Sheng LH, Lu ZY, Cheng NN. Efficacy and safety of telmisartan vs. losartan in control of mild-to-moderate hypertension: a multicentre, randomised, double-blind study. Int J Clin Pract Suppl. 2004;:46-9 | | 2 |
| e252 | Ding PY, Chu KM, Chiang HT, Shu KH. A double-blind ambulatory blood pressure monitoring study of the efficacy and tolerability of once-daily telmisartan 40 mg in comparison with losartan 50 mg in the treatment of mild-to-moderate hypertension in Taiwanese patients. Int J Clin Pract Suppl. 2004;:16-22 | | 2 |
| e253 | Li Y, Liu G, Jiang B, Gao R, Chen L, Su L, Li J. A comparison of initial treatment with losartan/HCTZ versus losartan monotherapy in chinese patients with mild to moderate essential hypertension. Int J Clin Pract. 2003;57:673-7 | | 2 |
| e254 | Bender AD, Carter CL, Hansen KB. Use of a diuretic combination of triamterene and hydrochlorothiazide in elderly patients . J Am Geriatr Soc 1967;15(2):166–73 | | 3 |
| e255 | Anderson J, Godfrey BE, Hill DM, Munro-Faure AD, Sheldon J. A comparison of the effects of hydrochlorothiazide and of frusemide in the treatment of hypertensive patients. Q J Med. 1971;40:541-60 | | 1, 3 |
| e256 | Weir MR, Weber MA, Punzi HA, Serfer HM, Rosenblatt S, Cady WJ. A dose escalation trial comparing the combination of diltiazem SR and hydrochlorothiazide with the monotherapies in patients with essential hypertension. J Hum Hypertens. 1992 Apr;6:133-8 | | 3 |
| e257 | Oparil S, Levine JH, Zuschke CA, Gradman AH, Ripley E, Jones DW, Hardison JD, Cushing DJ, Prasad R, Michelson EL. Effects of candesartan cilexetil in patients with severe systemic hypertension. Am J Cardiol. 1999;84:289-93 | | 3 |
| e258 | Captopril Research Group of Japan (Chairman: M. Murakami. Clinical effects of low-dose captopril plus a thiazide diuretic on mild to moderate essential hypertension: a multicenter double-blind comparison with propranolol. J Cardiovasc Pharmacol. 1985;7 Suppl 1:S77-81 | | 2 |
| e259 | The GLANT Study. Study Group on Long-term Antihypertensive Therapy. A 12-month comparison of ACE inhibitor and CA antagonist therapy in mild to moderate essential hypertension. Hypertens Res. 1995;18:235-44 | | 2 |
| e260 | Maxwell MH, Brachfeld J, Itskovitz H, Lunn JA, Moser M, Zawada ET. Blood pressure lowering and potassium conservation by triamterene-hydrochlorothiazide and amiloride-hydrochlorothiazide in hypertension. Clin Pharmacol Ther. 1985;37:61-5 | | 3 |
| e261 | Oparil S, Guthrie R, Lewin AJ, Marbury T, Reilly K, Triscari J, Witcher JA. An elective-titration study of the comparative effectiveness of two angiotensin II-receptor blockers, irbesartan and losartan. Clin Ther. 1998;20:398-409 | | 3 |
| e262 | Pool J1, Oparil S, Hedner T, Glazer R, Oddou-Stock P, Hester A. Dose-responsive antihypertensive efficacy of valsartan, a new angiotensin II-receptor blocker. Clin Ther. 1998;20:1106-14 | | 3 |
| e263 | Ramsay LE, Yeo WW; Losartan Cough Study Group. Double-blind comparison of losartan, lisinopril and hydrochlorothiazide in hypertensive patients with a previous angiotensin converting enzyme inhibitor-associated cough. J Hypertens Suppl. 1995;13:S73-6 | | 6 |
| e264 | Ohma KP, Milon H, Valnes K. Efficacy and tolerability of a combination tablet of candesartan cilexetil and hydrochlorothiazide in insufficiently controlled primary hypertension--comparison with a combination of losartan and hydrochlorothiazide. Blood Press. 2000;9:214-20 | | 3 |
| e265 | Hawkins DW, Hall WD, Douglas MB, Cotsonis G. A multi-center analysis of the use of enalapril and lisinopril in elderly hypertensive patient. J Am Geriatr Soc. 1994;42:1273-6 | | 3 |
| e266 | Gomez HJ, Smith SG 3rd, Moncloa F. Efficacy and safety of lisinopril in older patients with essential hypertension. Am J Med. 1988;85:35-7 | | 3 |
| e267 | Hall WD, Blackburn KL, Feig PU, Walsh PP, Mac Carthy EP Safety and efficacy of a new once-daily nifedipine slow-release formulation (NIF SR) compared to lisinopril (LIS) for the treatment of hypertension in elderly patients. Am J Hypertens 5:115A, 1992 | | 3 |
| e268 | Thomson M, Droussin AM, De Lame PA. The antihypertensive effect and safety of lisinopril in patients with mild to moderate essential hypertension. Acta Cardiol. 1990;45:297-309 | | 3 |
| e269 | Laher MS, Donohoe JF, Kelly JG, Doyle GD. Antihypertensive and renal effects of lisinopril in older patients with hypertension. Am J Med. 1988;85:38-43 | | 3 |
| e270 | Bakris G, Gradman A, Reif M, Wofford M, Munger M, Harris S, Vendetti J, Michelson EL, Wang R; CLAIM Study Investigators. Antihypertensive efficacy of candesartan in comparison to losartan: the CLAIM study. J Clin Hypertens (Greenwich). 2001;3:16-21 | | 3 |
| e271 | White WB, Lacourciere Y, Davidai G. Effects of the angiotensin II receptor blockers telmisartan versus valsartan on the circadian variation of blood pressure: impact on the early morning period. Am J Hypertens. 2004;17:347-53 | | 3 |
| e272 | Dahlöf B, Devereux RB, Kjeldsen SE, Julius S, Beevers G, de Faire U, Fyhrquist F, Ibsen H, Kristiansson K, Lederballe-Pedersen O, Lindholm LH, Nieminen MS, Omvik P, Oparil S, Wedel H; LIFE Study Group. Cardiovascular morbidity and mortality in the Losartan Intervention For Endpoint reduction in hypertension study (LIFE): a randomised trial against atenolol. Lancet. 2002;359:995-1003 | | 3 |
| e273 | Manolis AJ, Grossman E, Jelakovic B, Jacovides A, Bernhardi DC, Cabrera WJ, Watanabe LA, Barragan J, Matadamas N, Mendiola A, Woo KS, Zhu JR, Mejia AD, Bunt T, Dumortier T, Smith RD. Effects of losartan and candesartan monotherapy and losartan/hydrochlorothiazide combination therapy in patients with mild to moderate hypertension. Clin Ther. 2000;22:1186-203 | | 3 |
| e274 | Poirier L, de Champlain J, Larochelle P, Lamarre-Cliche M, Lacourcière Y. A comparison of the efficacy and duration of action of telmisartan, amlodipine and ramipril in patients with confirmed ambulatory hypertension. Blood Press Monit. 2004;9:231-6 | | 1 |
| e275 | Weber MA, Byyny RL, Pratt JH, Faison EP, Snavely DB, Goldberg AI, Nelson EB. Blood pressure effects of the angiotensin II receptor blocker, losartan. Arch Intern Med. 1995;155:405-11 | | 1 |
| e276 | Weir MR, Lavin PT. Comparison of the efficacy and tolerability of Prinivil and Procardia XL in black and white hypertensive patients. Clin Ther. 1992;14:730-9 | | 1 |
| e277 | Derosa G, Ragonesi PD, Mugellini A, Ciccarelli L, Fogari R. Effects of telmisartan compared with eprosartan on blood pressure control, glucose metabolism and lipid profile in hypertensive, type 2 diabetic patients: a randomized, double-blind, placebo-controlled 12-month study. Hypertens Res. 2004;27:457-64 | | 1 |
| e278 | Fogari R, Mugellini A, Zoppi A, Marasi G, Pasotti C, Poletti L, Rinaldi A, Preti P. Efficacy of losartan, valsartan, and telmisartan in patients with mild to moderate hypertension: A double-blind, placebo-controlled, crossover study using ambulatory blood pressure monitoring. Curr Med Res. 2002;63:1-14 | | 1 |
| e279 | Freier PA, Wollam GL, Hall WD, Unger DJ, Douglas MB, Bain RP. Freier PA, Wollam GL, Hall WD, Unger DJ, Douglas MB, Bain RP. Clin Pharmacol Ther. 1984;36:731-7 | | 1 |
| e280 | Scaglione R, Indovina A, Parrinello G, Lipari R, Mulè LG, Ganguzza A, Capuana G, Stampino CG, Licata G. Antihypertensive efficacy and effects of nitrendipine on cardiac and renal hemodynamics in mild to moderate hypertensive patients: randomized controlled trial versus hydrochlorothiazide. Cardiovasc Drugs Ther. 1992;6:141-6 | | 1 |
| e281 | Leary WP, Reyes AJ. Antihypertensive and metabolic effects of a combination of hydrochlorothiazide and amiloride. S Afr Med J. 1981;60:381-4 | | 1 |
| e282 | Stein CM, Neill P, Kusemamuriwo T. Antihypertensive effects of low doses of hydrochlorothiazide in hypertensive black Zimbabweans. Int J Cardiol. 1992;37:231-5 | | 1 |
| e283 | Middlemost SJ, Tager R, Davis J, Sareli P. Effectiveness of enalapril in combination with low-dose hydrochlorothiazide versus enalapril alone for mild to moderate systemic hypertension in black patients.Am J Cardiol. 1994;73:1092-7 | | 1 |
| e284 | Pedersen OL. Comparison of metoprolol as hydrochlorothiazide and antihypertensive agents. Eur J Clin Pharmacol. 1976;10:381-5 | | 1 |
| e285 | Forslund T, Franzén P, Backman R. Comparison of fosinopril and hydrochlorothiazide in patients with mild to moderate hypertension. J Intern Med. 1991;230:511-7 | | 1 |
| e286 | Liedholm H, Ursing D. Antihypertensive effect and tolerability of two fixed combination of metoprolol and hydrochlorothiazide followed by a long-term tolerance study with one combination. Ann Clin Res. 1981;13 Suppl 30:45-53 | | 1 |
| e287 | Poncelet P, Werquin S, Warembourg A, Ansquer JC, Boussac I, Lekieffre J, Carre A. A double-blind, randomized, comparative study of nitrendipine and enalapril in elderly hypertensive patients. J Cardiovasc Pharmacol. 1991;18 Suppl 1:S67-70 | | 1 |
| e288 | Radevski IV, Valtchanova SP, Candy GP, Tshele EF, Sareli P. Comparison of acebutolol with and without hydrochlorothiazide versus carvedilol with and without hydrochlorothiazide in black patients with mild to moderate systemic hypertension. Am J Cardiol. 1999;84:70-5 | | 1 |
| e289 | Chalmers JP, Horvath JS, Korner PI, Tiller DJ, Bune AJ, England JD, Fletcher PJ. Quantitative effects of timolol and hydrochlorothiazide on blood pressure, heart rate and plasma renin activity: results of a double-blind factorial trial in patients with essential hypertension. Clin Sci Mol Med Suppl. 197;3:517s-519s | | 1 |
| e290 | Chalmers JP, Korner PI, Tiller DJ, Bune AJ, Steiner JD, West MJ, Wing LM, Uther JF. Double-blind factorial trial of prindolol and hydrochlorothiazide in hypertension. Med J Aust. 1976;1:650-3 | | 1 |
| e291 | Rasmussen S, Arnung K, Eskildsen PC, Nielsen PE. A comparative study of atenolol and metoprolol in the treatment of hypertension. Br J Clin Pharmacol. 1981;12:887-91 | | 1 |
| e292 | Rasmussen S, Arnung K, Eskildsen PC, Nielsen PE. A double-blind multicentre study of piretanide and hydrochlorothiazide in patients with essential hypertension. J Int Med Res. 1984;12:81-6 | | 1 |
| e293 | Durley Y, Cubberley RB, Thomas S.Antihypertensive effect of oral timolol maleate and hydrochlorothiazide once daily compared with hydrochlorothiazide once daily. Am J Hosp Pharm. 1981;38:1161-4 | | 1 |
| e294 | Watt SJ, Lee MR. A comparative trial of fixed ratio beta-adrenoceptor blocker and diuretic combination products in moderate hypertension. Pharmatherapeutica. 1980;2:487-93 | | 1 |
| e295 | Gilchrist NL, Nicholls MG, Ewer TC, Livesey JH, Sainsbury R. A comparison of long acting nifedipine and enalapril in elderly hypertensives: a randomised, single-blind, cross-over study. J Hum Hypertens. 1988;2:33-9 | | 1 |
| e296 | Richardson PJ1, Meany TB, Johnston GD, Kondowe G, Grimmer SF, Breckenridge AM. Comparative efficacy of lisinopril and nifedipine retard in essential hypertension: a double-blind, placebo-controlled trial. J Cardiovasc Pharmacol. 1987;10 Suppl 10:S96-8 | | 1 |
| e297 | Johnston GD, Banks DC, Davies S, Duffin D, Garnham JC, Nicholls DP, Raj MV, Mansy S, Sloan P, Strouthidis TM, et al. A double blind comparative study of lisinopril and enalapril in patients with essential hypertension. J Hum Hypertens. 1991;5:405-10 | | 1 |
| e298 | Rössner S, Weiner L. Atenolol and metoprolol: comparison of effects on blood pressure and serum lipoproteins, and side effects. Eur J Clin Pharmacol. 1983;24:573-7 | | 1 |
| e299 | Rizzini P, Castello C, Salvi S, Recchia G. Efficacy and safety of lacidipine, a new long-lasting calcium antagonist, in elderly hypertensive patients. J Cardiovasc Pharmacol. 1991;17 Suppl 4:S38-43 | | 1 |
| e300 | Dews I, Wiseman WT, al-Khawaja I, Stephens J, VandenBurg M. A comparison of single doses of lisinopril and enalapril in hypertension. J Hum Hypertens. 1989;3 Suppl 1:35-9 | | 1 |
| e301 | Cox JP, Duggan J, O'Boyle CA, Mee F, Walsh JB, Coakley D, O'Brien E, O'Malley K. A double-blind evaluation of captopril in elderly hypertensives. J Hypertens. 1989;7:299-303 | | 1 |
| e302 | McInnes GT, Findlay IN, Murray G, Cleland JG, Dargie HJ. Cardiovascular responses to verapamil and propranolol in hypertensive patients. J Hypertens Suppl. 1985;3:S219-21 | | 1 |
| e303 | Pool PE, Seagren SC, Salel AF. Effects of diltiazem on serum lipids, exercise performance and blood pressure: randomized, double-blind, placebo-controlled evaluation for systemic hypertension. Am J Cardiol. 1985;56:86H-91H | | 1 |
| e304 | Oren S, Viskoper JR, Zilles P. Antihypertensive efficacy of a once a day verapamil SR/trandolapril combination. Int J Cardiol. 1996;55:97-102 | | 1 |
| e305 | O'Brien E, Barton J, Nussberger J, Mulcahy D, Jensen C, Dicker P, Stanton A. Aliskiren reduces blood pressure and suppresses plasma renin activity in combination with a thiazide diuretic, an angiotensin-converting enzyme inhibitor, or an angiotensin receptor blocker. Hypertension. 2007;49:276-84 | | 1 |
| e306 | Katzman PL, Henningsen NC, Hulthén UL. Amiloride compared with nitrendipine in treatment of essential hypertension. J Hum Hypertens. 1988;2:147-51 | | 1 |
| e307 | MacKay JH, Arcuri KE, Goldberg AI, Snapinn SM, Sweet CS. Losartan and low-dose hydrochlorothiazide in patients with essential hypertension. A double-blind, placebo-controlled trial of concomitant administration compared with individual components. Arch Intern Med. 1996;156:278-85 | | 1 |
| e308 | Soffer BA, Wright JT Jr, Pratt JH, Wiens B, Goldberg AI, Sweet CS. Effects of losartan on a background of hydrochlorothiazide in patients with hypertension. Hypertension. 1995;26:112-7 | | 1 |
| e309 | Khalil SI, El Zein O, El Mahadi Bella M. A double-blind, crossover study of acebutolol and hydrochlorothiazide/amiloride diuretic in Sudanese patients with essential hypertension. Curr Med Res Opin. 1982;8:39-43 | | 1 |
| e310 | Davies J, Jensen H, Garsdal P. A double-blind comparison of amlodipine and placebo added to open enalapril in patients with moderate to severe essential hypertension. J Cardiovasc Pharmacol. 1991;17 Suppl 1:S16-8 | | 1, 3 |
| e311 | Lewis R, Maclean D, Ioannides C, Johnston A, McDevitt DG. A comparison of bisoprolol and atenolol in the treatment of mild to moderate hypertension. Br J Clin Pharmacol. 1988;26:53-9 | | 1 |
| e312 | Herrick AL, Waller PC, Berkin KE, Pringle SD, Callender JS, Robertson MP, Findlay JG, Murray GD, Reid JL, Lorimer AR, et al. Comparison of enalapril and atenolol in mild to moderate hypertension. Am J Med. 1989;86:421-6 | | 1 |
| e313 | Kaplan NM, Sproul LE, Mulcahy WS. Large prospective study of ramipril in patients with hypertension. CARE Investigators. Clin Ther. 1993;15:810-8 | | 1 |
| e314 | Carlsen, J. E.; Buchmann, M.; Hoglund, C.; Pellinen, T.; Honkanen, T.; Soerensen, O. H.; Leth, A.; Maltbaek, N., 24-Hour Antihypertensive Effect of Oral Cilazapril? Clin Drug Invest. 1995;10:221-227 | | 1 |
| e315 | Himmelmann A, Keinänen-Kiukaanniemi S, Wester A, Redón J, Asmar R, Hedner T; Effect Study Group. The effect duration of candesartan cilexetil once daily, in comparison with enalapril once daily, in patients with mild to moderate hypertension. Blood Press. 2001;10(1):43-51 | | 1, 3 |
| e316 | Weir MR, Fagan T, Chrysant S, Flamenbaum W, Kaihlanen PM, Lueg M, Anzalone D. Comparison of the efficacy and tolerability of an angiotensin converting enzyme inhibitor (lisinopril) versus a calcium channel antagonist (diltiazem SR) in the treatment of moderate to severe hypertension. J Hum Hypertens. 1994;8:531-7 | | 1 |
| e317 | Schnaper HW, Stein G, Schoenberger JA, Leon AS, Tuck ML, Taylor AA, Liss C, Shapiro DA. Comparison of enalapril and thiazide diuretics in the elderly hypertensive patient. Gerontology. 1987;33 Suppl 1:24-35 | | 1 |
| e318 | Fogari R, Taddei S, Holm-Bentzen M, Baszak J, Melani L, Schumacher K. Efficacy and safety of olmesartan medoxomil 40 mg/hydrochlorothiazide 12.5 mg combination therapy versus olmesartan medoxomil 40 mg monotherapy in patients with moderate to severe hypertension: a randomized, double-blind, parallel-group, multicentre, multinational, phase III study. Clin Drug Investig. 2010;30:581-97 | | 1 |
| e319 | Frick MH, Halttunen P, Himanen P, Huttunen M, Pörsti P, Pitkäjärvi T, Pöyhönen L, Pyykönen ML, Reinikainen P, Salmela P, et al. A long-term double-blind comparison of doxazosin and atenolol in patients with mild to moderate essential hypertension. Br J Clin Pharmacol. 1986;21 Suppl 1:55S-62S | | 1 |
| e320 | Lacourcière Y. A multicenter, randomized, double-blind study of the antihypertensive efficacy and tolerability of irbesartan in patients aged > or = 65 years with mild to moderate hypertension. Clin Ther. 2000;22:1213-24 | | 1 |
| e321 | Jäättelä A, Pyörälä K. A controlled study on the antihypertensive effect of a new beta-adrenergic receptor blocking drug, metoprolol, in combination with chlorthalidone. Br J Clin Pharmacol. 1976;3:655-60 | | 1 |
| e322 | Kristensen BO, Brøns M, Christensen CK, Geday E, Jacobsen FK, Jensen SN, Linde NC. Antihypertensive effect of atenolol (100 mg once a day) and methyldopa (250 mg thrice a day). A double-blind cross-over multicentre study. Acta Med Scand. 1981;209:267-70 | | 1 |
| e323 | Lewis JE. Comparison of acebutolol and hydrochlorothiazide in essential hypertension. Am Heart J. 1985;109:1168-74 | | 1 |
| e324 | Ishimitsu T, Numabe A, Masuda T, Akabane T, Okamura A, Minami J, Matsuoka H. Angiotensin-II receptor antagonist combined with calcium channel blocker or diuretic for essential hypertension. Hypertens Res. 2009;32:962-8 | | 1 |
| e325 | Lyons D, Fowler G, Webster J, Hall ST, Petrie JC. An assessment of lacidipine and atenolol in mild to moderate hypertension. Br J Clin Pharmacol. 1994;37:45-51 | | 1 |
| e326 | Salerno CM, Demopoulos L, Mukherjee R, Gradman AH. Combination angiotensin receptor blocker/hydrochlorothiazide as initial therapy in the treatment of patients with severe hypertension. J Clin Hypertens (Greenwich). 2004;6):614-20 | | 1 |
| e327 | Dunlay MC, Fitzpatrick V, Chrysant S, Francischetti EA, Goldberg AI, Sweet CS. Losartan potassium as initial therapy in patients with severe hypertension J Hum Hypertens. 1995;9:861-7 | | 1 |
| e328 | Ball KJ, Williams PA, Stumpe KO. Relative efficacy of an angiotensin II antagonist compared with other antihypertensive agents. Olmesartan medoxomil versus antihypertensives. J Hypertens Suppl. 2001;19:S49-56 | | 1 |
| e329 | Stumpe KO, Ludwig M. Antihypertensive efficacy of olmesartan compared with other antihypertensive drugs. J Hum Hypertens. 2002;16 Suppl 2:S24-8 | | 1 |
| e330 | Saito I, Kushiro T, Hirata K, Sato Y, Kobayashi F, Sagawa K, Hiramatsu K, Komiya M. The use of olmesartan medoxomil as monotherapy or in combination with other antihypertensive agents in elderly hypertensive patients in Japan. J Clin Hypertens (Greenwich). 2008;10:272-9 | | 1 |
| e331 | Kato J, Yokota N, Tamaki N, Kariya S, Kita T, Ayabe T, Eto T, Kitamura K. Comparison of combination therapies, including the angiotensin receptor blocker olmesartan and either a calcium channel blocker or a thiazide diuretic, in elderly patients with hypertension. Hypertens Res. 2011;34:331-5 | | 1 |
| e332 | Kereiakes DJ, Neutel JM, Punzi HA, Xu J, Lipka LJ, Dubiel R. Efficacy and safety of olmesartan medoxomil and hydrochlorothiazide compared with benazepril and amlodipine besylate. Am J Cardiovasc Drugs. 2007;7:361-72 | | 1 |
| e333 | Hunter Hypertension Research Group. Randomised, double-blind crossover comparison of once-daily captopril and lisinopril in patients with mild to moderate hypertension--a community-based study. Clin Exp Hypertens. 1993;15:423-34 | | 1 |
| e334 | Prichard BN, Jäger BA, Luszick JH, Küster LJ, Verboom CN, Hughes PR, Sauermann W, Küppers HE. Placebo-controlled comparison of the efficacy and tolerability of once-daily moxonidine and enalapril in mild to moderate essential hypertension. Blood Press. 2002;11:166-72 | | 1 |
| e335 | Frimodt-Moeller J, Poulsen DL, Kornerup HJ, Bech P. Quality of life, side effects and efficacy of lisinopril compared with metoprolol in patients with mild to moderate essential hypertension. J Hum Hypertens. 1991;5:215-21 | | 1 |
| e336 | Howes LG, Nguyen T, Jackson B. Safety and efficacy of quinapril in hypertensive geriatric patients. J Am Geriatr Soc. 1996;44:1135 | | 1 |
| e337 | Nifedipine-Atenolol Study Review Committee. Nifedipine and atenolol singly and combined for treatment of essential hypertension: comparative multicentre study in general practice in the United Kingdom. Br Med J (Clin Res Ed). 1988;296:468-72 | | 1 |
| e338 | Karlberg BE, Nilsson O, Tolagen K, Nitelius E, Waern U. bOnce-daily metoprolol in primary hypertension. Clin Pharmacol Ther. 1979;25:399-407 | | 1 |
| e339 | Rasmussen S, Rasmussen K. Influence of metoprolol, alone and in combination with a thiazide diuretic, on blood pressure, plasma volume, extracellular volume and glomerular filtration rate in essential hypertension. Eur J Clin Pharmacol. 1979;15:305-10 | | 1 |
| e340 | Rumboldt Z, Rumboldt M, Jurisic M. Indapamide versus beta-blocker therapy: a double-blind, crossover study in essential hypertension. Curr Med Res Opin. 1984;9:10-20 | | 1 |
| e341 | Ott P, Storm TL, Krusell LR, Jensen H, Badskjaer J, Faergeman O. Multicenter, double-blind comparison of doxazosin and atenolol in patients with mild to moderate hypertension.Am J Cardiol. 1987;59:73G-77G | | 1 |
| e342 | Kubik M, Kendall M, Ebbutt A, John V. Metoprolol with and without chlorthalidone in hypertension. Clin Pharmacol Ther. 1979;25:25-32 | | 1 |
| e343 | Lessem JN, Rucinska E, Vickers FF, Espie J, Sromovsky JA. Long-term enalapril--a new converting enzyme inhibitor--in the treatment of mild to moderate essential hypertension, results of a worldwide multiclinic study. Comparing two ways of analyzing data. Clin Exp Hypertens A. 1985;7:1515-39 | | 1 |
| e344 | Palombo C, Marabotti C, Genovesi-Ebert A, Giuliano G, Giaconi S, Fommei E, Mezzasalma L, Ghione S. Long-term hypotensive treatment with nitrendipine in mild to moderate essential hypertension: preliminary results of a placebo-controlled study versus atenolol. J Cardiovasc Pharmacol. 1987;10 Suppl 10:S90-5 | | 1 |
| e345 | Prisant LM, Neutel JM, Ferdinand K, Papademetriou V, DeQuattro V, Hall WD, Weir MR. Low-dose combination therapy as first-line hypertension treatment for blacks and nonblacks J Natl Med Assoc. 1999; 91: 40-48 | | 1 |
| e346 | Jueng C, Halperin AK, Hashimoto F, Callender K.Nifedipine GITS and hydrochlorothiazide in essential hypertension. J Clin Hypertens. 1987;3:695-703 | | 1 |
| e347 | Neutel JM, Smith DH, Reilly PA. The efficacy and safety of telmisartan compared to enalapril in patients with severe hypertension. Int J Clin Pract. 1999;53:175-8 | | 1 |
| e348 | Swedish Lisinopril Study Group. Lisinopril combined with atenolol in the treatment of hypertension. J Cardiovasc Pharmacol. 1991;18:457-61 | | 1 |
| e349 | Oparil S, Lee J, Karki S, Melino M. Subgroup analyses of an efficacy and safety study of concomitant administration of amlodipine besylate and olmesartan medoxomil: evaluation by baseline hypertension stage and prior antihypertensive medication use. J Cardiovasc Pharmacol. 2009 ;54:427-36 | | 5 |
| e350 | Owens P, Kelly L, Nallen R, Ryan D, Fitzgerald D, O'Brien E. Comparison of antihypertensive and metabolic effects of losartan and losartan in combination with hydrochlorothiazide--a randomized controlled trial. J Hypertens. 2000;18:339-45 | | 3 |
| e351 | Leenen FH, Myers MG, Joyner CD, Toal CB. Differential effects of once-daily antihypertensive drugs on blood pressure, left ventricular mass and sympathetic activity: Nifedipine-GITS versus felodipine-ER versus enalapril. Can J Cardiol. 2002;18:1285-93 | | 3 |
| e352 | Asplund J. A fixed-ratio combination of metoprolol and hydrochlorothiazide (Co-betaloc(TM)) in essential hypertension: A comparison between the individual drugs. Cuur Ther Res. 1981;29:387-394 | | 1 |
| e353 | Homuth V, Faulhaber HD, Loose U, Löffler K, Luft FC. Usefulness of piretanide plus ramipril for systemic hypertension: a multicenter trial. Am J Cardiol. 1993;72:666-71 | | 6 |
| e354 | Morgan TO, Louis WJ, MacDonald GJ, Conway EL, Bartholomeusz LC, Anderson AI, Cameron DP, Donnelly T, Frewin DB, Hooper MJ, et al. Antihypertensive efficacy and safety of perindopril in mild-to-moderate essential hypertension: results of a double-blind multicenter study versus atenoll. Am J Med. 1992;92:73S-78S | | 3 |
| e355 | Scholze J, Bauer B, Massaro J. Antihypertensive profiles with ascending dose combinations of ramipril and felodipine ER. Clin Exp Hypertens. 1999;21:1447-62 | | 3 |
| e356 | Thurston H, Mimran A, Zanchetti A, Creytens G, Rorive G, Brown CL, Santoni JP. A double blind comparison of perindopril and atenolol in essential hypertension. J Hum Hypertens. 1990;4:547-52 | | 3 |
| e357 | Roca-Cusachs A, Torres F, Horas M, Ríos J, Calvo G, Delgadillo J, Terán M; Spanish Nitrendipine/Enalapril Collaborative Study Group. Nitrendipine and enalapril combination therapy in mild to moderate hypertension: assessment of dose-response relationship by a clinical trial of factorial design. J Cardiovasc Pharmacol. 2001;38:840-9 | | 6 |
| e358 | P Dessi-Fulgheri, N Glorioso, P Madeddu, M Oppes, G Manca, et al. Antihypertensive efficacy of slow-release metoprolol and chlorthalidone as fixed combination: a randomized double blind comparison with chlorthalidone .Curr ther res 1983; 34:515-522 | | 1 |
| e359 | Harth. C. ; Vander Linde D. L. ; Lustermans F. A. T. ; Vander Waag. ; Wester A. Double-blind comparison of dytenzide, atenolol and the combination of ditenzide and atenolol in patients with essential hypertension Curr ther res 1985; 38:702-709 | | 1 |
| e360 | Lacourcière Y, Neutel JM, Schumacher H. Comparison of fixed-dose combinations of telmisartan/hydrochlorothiazide 40/12.5 mg and 80/12.5 mg and a fixed-dose combination of losartan/hydrochlorothiazide 50/12.5 mg in mild to moderate essential hypertension: pooled analysis of two multicenter, prospective, randomized, open-label, blinded-end point (PROBE) trials. Clin Ther. 2005;27:1795-805 | | 5 |
| e361 | Lacourcière Y, Poirier L, Lefebvre J, Burford RG. Clinical efficacy of force titrated doses of diltiazem extended-release. A placebo controlled study. Am J Hypertens. 1995;8:282-6 | | 1 |
| e362 | Fernández M, Madero R, González D, Camacho P, Villalpando J, Arriaga J. Combined versus single effect of fosinopril and hydrochlorothiazide in hypertensive patients. Hypertension. 1994 ;23:I207-10 | | 1 |
| e363 | Küppers HE, Jäger BA, Luszick JH, Gräve MA, Hughes PR, Kaan EC. Placebo-controlled comparison of the efficacy and tolerability of once-daily moxonidine and enalapril in mild-to-moderate essential hypertension. J Hypertens. 1997;15:93-7 | | 3 |
| e364 | Gradman AH, Cutler NR, Davis PJ, Robbins JA, Weiss RJ, Wood BC. Combined enalapril and felodipine extended release (ER) for systemic hypertension. Enalapril-Felodipine ER Factorial Study Group. Am J Cardiol. 1997;79:431-5 | | 3 |
| e365 | Pool J, Kaihlanen P, Lewis G, Ginsberg D, Oparil S, Glazer R, Messerli FH. Once-daily treatment of patients with hypertension: a placebo-controlled study of amlodipine and benazepril vs amlodipine or benazepril alone. J Hum Hypertens. 200;15:495-8 | | 6 |
| e366 | Villamil A, Chrysant SG, Clahoun D, Schober B, Hsu H, Matrisciano-Dimichino L, Zhang J. Renin inhibition with aliskiren provides additive antihypertensive efficacy when used in combination with hydrochlorothiazide. J Hypertens. 2007;25:217-26 | | 6 |
| e367 | Fernandez PG, Bolli P, Lee C.The 24 h blood pressure responses of hypertensives to a once-a-day cilazapril regimen. Can J Cardiol. 1990;6:53-8 | | 1 |
| e368 | Miller WE. Randomised, Double-Blind, Comparison of Fosinopril and Propranolol Added to Diuretic Therapy for the Treatment of Moderate-to-Severe Hypertension. Drug Inves 1991;3:32-37 | | 3 |
| e369 | Brown CL, Backhouse CI, Grippat JC, Santoni JP. The effect of perindopril and hydrochlorothiazide alone and in combination on blood pressure and on the renin-angiotensin system in hypertensive subjects. Eur J Clin Pharmacol. 1990;39:327-32 | | 1 |
| e370 | Scholze J, Zilles P, Compagnone D. Verapamil SR and trandolapril combination therapy in hypertension--a clinical trial of factorial design. German Hypertension Study Group. Br J Clin Pharmacol. 1998;45:491-5 | | 3 |
| e371 | Levine B. Effect of eprosartan and enalapril in the treatment of black hypertensive patients: subgroup analysis of a 26-week, double-blind, multicentre study. Curr Med Res Opin. 1999;15:25-32 | | 3 |
| e372 | Lorimer AR, Anderson JA, Laher MS, Davies J, Lazarus JH, Taylor SH, Sanghera S. Double-blind comparison of amlodipine and nifedipine retard in the treatment of mild to moderate hypertension. J Hum Hypertens. 1994 ;8:65-8 | | 3 |
| e373 | Karlberg BE, Andrup M, Odén A. Efficacy and safety of a new long-acting drug combination, trandolapril/verapamil as compared to monotherapy in primary hypertension. Blood Press. 2000;9:140-5 | | 3 |
| e374 | Kojima S, Kawano Y, Yoshitomi Y, Kuramochi M, Matsuoka H, Omae T. Comparison of first-line antihypertensive drugs by a randomized cross-over method--a preliminary report. Hypertens Res. 1995;18:303-5 | | 2 |
| e375 | Koenig W, Sund M, Binner L, Hehr R, Rosenthal J, Hombach V. Comparison of once daily felodipine 10 mg ER and hydrochlorothiazide 25 mg in the treatment of mild to moderate hypertension. Eur J Clin Pharmacol. 1991;41:197-9 | | 1 |
| e376 | Maclean D, Mitchell ET, Laing EM, Macdonald FC, Gough KJ, Dow RJ, McDevitt DG. Comparison of the efficacy and acceptability of nicardipine and propranolol, alone and in combination, in mild to moderate hypertension. Br J Clin Pharmacol. 1989;27:569-80 | | 1 |
| e377 | Malacco E, Santonastaso M, Varì NA, Gargiulo A, Spagnuolo V, Bertocchi F, Palatini P; Blood Pressure Reduction and Tolerability of Valsartan in Comparison with Lisinopril Study. Comparison of valsartan 160 mg with lisinopril 20 mg, given as monotherapy or in combination with a diuretic, for the treatment of hypertension: the Blood Pressure Reduction and Tolerability of Valsartan in Comparison with Lisinopril (PREVAIL) study. Clin Ther. 2004;26:855-65 | | 3 |
| e378 | Malacco E, Omboni S, Volpe M, Auteri A, Zanchetti A; ESPORT Study Group. Antihypertensive efficacy and safety of olmesartan medoxomil and ramipril in elderly patients with mild to moderate essential hypertension: the ESPORT study. J Hypertens. 2010;28:2342-50 | | 3 |
| e379 | Lees KR, Reid JL, Scott MG, Hosie J, Herpin D, Santoni JP. Captopril versus perindopril: a double blind study in essential hypertension. J Hum Hypertens. 1989;3:17-22 | | 3 |
| e380 | Alcocer L, Campos C, Bahena JH, Nacaud A, Parra Carillo J, Calvo C, Weber C, Lerebours G, Mickalonis L, Villahermosa MT. Clinical acceptability of ACE inhibitor therapy in mild to moderate hypertension, a comparison between perindopril and enalapril. Cardiovasc Drugs Ther. 1995;9:431-6 | | 3 |
| e381 | Vaicaitis JS. VVaicaitis JS. Evaluation of a beta-blocker, timolol maleate, combined with hydrochlorothiazide in essential hypertension Cuur Ther Res. 1980;27:365-373 | | 1 |
| e382 | Muiesan G, Magnani B, Agabiti-Rosei E, Alicandri C, Ambrosioni E, Miele N. Evaluation of the effect of timolol alone and in combination with hydrochlorothiazide and amiloride in the treatment of mild to moderate arterial hypertension: a double-blind, controlled study. Clin Sci Mol Med Suppl. 1976;3:529s-531s | | 1 |
| e383 | Zannad F, Bernaud CM, Fay R. Double-blind, randomized, multicentre comparison of the effects of amlodipine and perindopril on 24 h therapeutic coverage and beyond in patients with mild to moderate hypertension. General Physicians Investigators' Group. J Hypertens. 1999;17:137-46 | | 3 |
| e384 | Guitard C, Sassano P, Tzincoca C, Duchiez J, Safar ME. Placebo-controlled crossover comparison of spirapril at 3, 6, 12 and 24 mg once daily in mild to severe essential hypertension. Blood Press Suppl. 1994;2:61-8 | | 1 |
| e385 | Zabludowski J, Rosenfeld J, Akbary MA, Rangoonwala B, Schinzel S. A multi-centre comparative study between ramipril and enalapril in patients with mild to moderate essential hypertension. Curr Med Res Opin. 1988;1:93-106 | | 6 |
| e386 | Scholze J, Breitstadt A, Cairns V, Bauer B, Bender N, Priestley C, Moreadith C, Phillips J, Vander Elst E, Koch G. Short report: ramipril and hydrochlorothiazide combination therapy in hypertension: a clinical trial of factorial design. J Hypertens. 1993;11:217-21 | | 6 |
| e387 | Ding YA, Chang SM, Chou TC. Comparison of amlodipine and quinapril on ambulatory blood pressure and platelet function in hypertension. J Hum Hypertens. 1995;9:637-41 | | 3 |
| e388 | Mallion JM, Heagerty A, Laeis P. Systolic blood pressure reduction with olmesartan medoxomil versus nitrendipine in elderly patients with isolated systolic hypertension. J Hypertens. 2007;25:2168-77 | | 3 |
| e389 | Edwards KG, Tweed JA, Saul PA, Wright FW. A comparative study of atenolol/nifedipine and atenolol/diuretic in hypertension. Pharmatherapeutica. 1986;4:637-41 | | 3 |
| e390 | Langdon CG, Baxter GA, Young PH. A multicenter comparison of carvedilol with hydrochlorothiazide in the treatment of mild-to-moderate essential hypertension. J Cardiovasc Pharmacol. 1991;18 Suppl 4:S51-6 | | 3 |
| e391 | Dafgärd T, Forsén B, Lindahl T. Comparative study of hydrochlorothiazide and a fixed combination of metoprolol and hydrochlorothiazide essential hypertension. Ann Clin Res. 1981;13 Suppl 30:37-44 | | 1 |
| e392 | Kang SM, Youn JC, Chae SC, Park CG, Yang JY, Kim MH, Hong TJ, Kim CH, Kim JJ, Shin DG, Jeong JW, Yoon JH, Park SH, Kwon J, Cho SY. Comparative Efficacy and Safety Profile of Amlodipine 5 mg/Losartan 50 mg Fixed-Dose Combination and Amlodipine 10 mg Monotherapy in Hypertensive Patients Who Respond Poorly to Amlodipine 5 mg Monotherapy: An 8-Week, Multicenter, Randomized, Double-Blind Phase III Noninferiority Study Clin Ther. 2011;33:1953-63 | | 2 |
| e393 | Izzo JL Jr, Purkayastha D, Hall D, Hilkert RJ. Comparative efficacy and safety of amlodipine/benazepril combination therapy and amlodipine monotherapy in severe hypertension. J Hum Hypertens. 2010;24:403-9 | | 3 |
| e394 | Lechi A, Pomari S, Berto R, Buniotto P, Parrinello A, Marini F, Cogo L, Tomasi A, Baretta G. Clinical evaluation of labetalol alone and combined with chlorthalidone in essential hypertension: a double-blind multicentre controlled study. Eur J Clin Pharmacol. 1982;22:289-93 | | 1 |
| e395 | Luurila OJ, Härkönen R, Hilden M, Icen R, Kohvakka A, Linna M, Sillanpää J, Vänskä O, Lukkala K. Carvedilol and atenolol once daily in the treatment of hypertension. J Hypertens Suppl. 1989;7:S264-5 | | 1 |
| e396 | Leonetti G. Comparative study of lacidipine and nifedipine SR in the treatment of hypertension: an Italian multicenter study. The Northern Italian Study Group of Lacidipine in Hypertension. J Cardiovasc Pharmacol. 1991;17 Suppl 4:S31-4 | | 3 |
| e397 | Lederle RM. Captopril and hydrochlorothiazide in the fixed combination multicenter trial. J Cardiovasc Pharmacol. 1985;7 Suppl 1:S63-9 | | 1 |
| e398 | Leenen FH, Tanner J, McNally CF. Antihypertensive efficacy of the ACE-inhibitor perindopril in the elderly. J Hum Hypertens. 2000;14:321-5 | | 3 |
| e399 | Izzo JL Jr, Weinberg MS, Hainer JW, Kerkering J, Tou CK; AMAZE. Antihypertensive efficacy of candesartan-lisinopril in combination vs. up-titration of lisinopril: the AMAZE trials. J Clin Hypertens (Greenwich). 2004;6:485-93 | | 3 |
| e400 | Lopez LM1, Thorman AD, Mehta JL. Effects of amlodipine on blood pressure, heart rate, catecholamines, lipids and responses to adrenergic stimulus. Am J Cardiol. 1990;66:1269-71 | | 1 |
| e401 | Larochelle P, Flack JM, Marbury TC, Sareli P, Krieger EM, Reeves RA. Effects and tolerability of irbesartan versus enalapril in patients with severe hypertension. Am J Cardiol. 1997;80:1613-5 | | 3 |
| e402 | Frishman WH, Bryzinski BS, Coulson LR, DeQuattro VL, Vlachakis ND, Mroczek WJ, Dukart G, Goldberg JD, Alemayehu D, Koury K. A multifactorial trial design to assess combination therapy in hypertension. Treatment with bisoprolol and hydrochlorothiazide. Arch Intern Med. 1994;154:1461-8 | | 6 |
| e403 | The Enalapril-Hydrochlorothiazide in Essential Hypertension Canadian Working **Group**. Enalapril and enalapril-hydrochlorothiazide in the treatment of essential hypertension. Clin Ther. 1993;15:364-73 | | 2 |
| e404 | Cilliers AJ. Atenolol as primary therapy in previously untreated hypertensives and as an adjuvant to other therapy. S Afr Med J. 1979;55:321-4 | | 2 |
| e405 | Karlberg BE, Lins LE, Hermansson K. Efficacy and safety of telmisartan, a selective AT1 receptor antagonist, compared with enalapril in elderly patients with primary hypertension.J Hypertens. 1999;17:293-302 | | 3 |
| e406 | Hornung RS, Jones RI, Gould BA, Sonecha T, Raftery EB. Twice-daily verapamil for hypertension: a comparison with propranolol. Am J Cardiol. 1986;57:93D-98D | | 6 |
| e407 | Naidu MU, Usha PR, Rao TR, Shobha JC. Evaluation of amlodipine, lisinopril, and a combination in the treatment of essential hypertension. Postgrad Med J. 2000;76:350-3 | | 1 |
| e408 | Veterans Administration Cooperative Study Group. Oxprenolol vs propranolol: a randomized, double-blind, multiclinic trial in hypertensive patients taking hydrochlorothiazide. J Hypertens 1981;3:250-256 | | 6 |
| e409 | Weir MR, Cassidy CA, Hall PS, Lancaster A, Schubert C, Urick A, Saunders E. Efficacy and tolerability of enalapril and sustained-release verapamil in older Patients with Mild to Moderate Essential Hypertension. Clin Ther 1990;12:139-148 | | 3 |
| e410 | Andrén L, Karlberg B, Ohman P, Svensson A, Asplund J, Hansson L. Captopril and atenolol combined with hydrochlorothiazide in essential hypertension. Br J. Clin. Pharmac. 1982;14:107S-111S | | 3 |
| e411 | Stevenson J G, Chideckel E W.Evaluation of cilazapril versus captopril in patients with mild to moderate essential hypertension. Clin. And Exper Hypertension. 1994; 16: 179-196 | | 3 |
| e412 | Paterna, S.; Licata, A.; Arnone, S.; Cottone, C.; Corrao, S.; Licata, G. Lercanidipine in Two Different Dosage Regimens as a Sole Treatment for Severe Essential HypertensionJ Cardiovasc Pharmacol 1997; 29:S50-S53 | | 1 |
| e413 | Widmann L, van der Does R, Hörrmann M, Machwirth M Safety and antihypertensive efficacy of carvedilol and atenolol alone and in combination with hydrochlorothiazide. Eur J Clin Pharmacol 1990;38:S143-S146 | | 6 |
| e414 | Schmieder RE, Böhm M. Efficacy and safety of olmesartan medoxomil plus amlodipine in age, gender and hypertension severity defined subgroups of hypertensive patients. J Hum Hypertens. 2011;25:354-63 | | 5, 2 |
| e415 | Study Group Multicenter **(**Labetalol/Hydrochlorothiazide). Labetalol and hydrochlorothiazide in hypertension. Clin Pharmacol Ther. 1985;38:24-7 | | 5 |
| e416 | Chalmers J, Tiller D, Horvath J, Bune A. Effects of timolol and hydrochlorothiazide on blood-pressure and plasma renin activity. Double-blind factorial trial. Lancet. 1976;2:328-31 | | 5 |
| e417 | Svendsen UG, Ibsen H, Rasmussen S, Leth A, Nielsen MD, Dige-Petersen H, Giese J. Effects of combined therapy with amiloride and hydrochlorothiazide on plasma and total body potassium, blood pressure, and the renin-angiotensin-aldosterone system in hypertensive patients. Eur J Clin Pharmacol. 1986;30:151-6 | | 5 |
| e418 | Ibsen H, Westberg B. The efficacy and tolerability of long-term felodipine treatment in hypertension. The Scandinavian Multicenter Group. Cardiovasc Drugs Ther. 1990;4:641-7 | | 5 |
| e419 | Littlejohn TW 3rd, Majul CR, Olvera R, Seeber M, Kobe M, Guthrie R, Oigman W; study investigators. Telmisartan plus amlodipine in patients with moderate or severe hypertension: results from a subgroup analysis of a randomized, placebo-controlled, parallel-group, 4 x 4 factorial study. Postgrad Med. 2009;121:5-14 | | 5 |
| e420 | Cifkova R, Peleska J, Hradec J, Rosolová H, Pintérová E, Zeman K, Oddou-Stock P, Thirlwell J, Botteri F. Valsartan and atenolol in patients with severe essential hypertension. J Hum Hypertens. 1998;12:563-7 | | 5 |
| e421 | Stergiou GS, Makris T, Papavasiliou M, Efstathiou S, Manolis A. Comparison of antihypertensive effects of an angiotensin-converting enzyme inhibitor, a calcium antagonist and a diuretic in patients with hypertension not controlled by angiotensin receptor blocker monotherapy. J Hypertens. 2005;23:883-9 | | 5 |
| e422 | de la Sierra A, Gil-Extremera B, Calvo C, Campo C, García-Puig J, Márquez E, Oliván J, Roca Cusachs A, Sanz de Castro S, Pontes C, Delgadillo J. Comparison of the antihypertensive effects of the fixed dose combination enalapril 10 mg/nitrendipine 20 mg vs losartan 50 mg/hydrochlorothiazide 12.5mg, assessed by 24-h ambulatory blood pressure monitoring, in essential hypertensive patients. J Hum Hypertens. 2004;18:215-22 | | 1 |
| e423 | Szlachcic J, Hirsch AT, Tubau JF, Vollmer C, Henderson S, Massie BM. Diltiazem versus propranolol in essential hypertension: responses of rest and exercise blood pressure and effects on exercise capacity. Am J Cardiol. 1987;59:393-9 | | 5, 1 |
| e424 | Vlasses PH, Conner DP, Rotmensch HH, Fruncillo RJ, Danzeisen JR, Shepley KJ, Ferguson RK. Double-blind comparison of captopril and enalapril in mild to moderate hypertension. J Am Coll Cardiol. 1986;7:651-60 | | 5 |
| e425 | Stokes GS, Monaghan JC, Berman K, Ryan M, Campbell DJ. Double-blind crossover study of the interaction between perindopril and amlodipine on blood pressure and hormones related to fluid and electrolyte balance in patients with essential hypertension. J Hum Hypertens. 1998;12:129-34 | | 5 |
| e426 | Talseth T, Westlie L, Daae L. Doxazosin and atenolol as monotherapy in mild and moderate hypertension: a randomized, parallel study with a three-year follow-up. Am Heart J. 1991;121:280-5 | | 5 |
| e427 | Canadian Study Group on Perindopril. Once daily perindopril versus slow release diltiazem in the treatment of mild to moderate essential hypertension Can J Cardiol. 1994;10 Suppl D:8D-12D | | 5, 3 |
| e428 | Turner AS, Brocklehurst JC, Napier RN. Once-daily acebutolol and atenolol in essential hypertension: double-blind crossover comparison. Am Heart J. 1985;109:1178-83 | | 5 |
| e429 | Ueng KC, Lin LC, Voon WC, Lin MC, Liu YB, Su HM, Chang PY, Lin TH, Chen WL, Wu CC, Lai WT, Lin CS. An eight-week, multicenter, randomized, double-blind study to evaluate the efficacy and tolerability of fixed-dose amlodipine/benazepril combination in comparison with amlodipine as first-line therapy in chinese patients with mild to moderate hypertension. Blood Press Suppl. 2008;1:24-31 | | 5 |
| e430 | Tantucci C, Bruni B, Dottorini ML, Peccini F, Motolese M, Lecaillon JB, Sorbini CA, Grassi V. Comparative evaluation of cardioselectivity of metoprolol OROS and atenolol: a double-blind, placebo-controlled crossover study. Am Heart J. 1990;120:467-72 | | 5 |
| e431 | Smilde JG. Comparison of the antihypertensive effect of a double dose of metoprolol versus the addition of hydrochlorothiazide to metoprolol. Eur J Clin Pharmacol. 1983;25:581-3 | | 5 |
| e432 | Jäättelä A.The Combination of Sotalol and Hydrochlorothiazide in the Treatment of Hypertension. J Clin Pharmacol. 1979;19:565 | | 5 |
| e433 | Sutandar H, Sugeng I, Utama H, Suryaatmaja M, Sukaman. The efficacy and acceptability of the combination of acebutolol and hydrochlorothiazide in the treatment of essential hypertension. Curr Med Res Opin. 1984;9:323-8 | | 5 |
| e434 | Henning R, Karlberg BE, Odar-Cederlöf I, Andersson PO, Lins LE, Nilsson OR, Tolagen K. Timolol and hydrochlorothiazide-amiloride in primary hypertension. Clin Pharmacol Ther. 1980;28:707-14 | | 5 |
| e435 | Valmin K, Hansen T. Treatment of benign essential hypertension: comparison of furosemide and hydrochlorothiazide. Eur J Clin Pharmacol. 1975;8:393-401 | | 5 |
| e436 | Walker JF, Kulaga SF, Kramsch DM. The efficacy and safety of enalapril in moderate to severe essential hypertension J Hypertens Suppl. 1984;2:S107-11 | | 5 |
| e437 | Basson W, Myburgh DP. Synergism of a beta blocker and a diuretic in the once-a-day treatment of essential hypertension. J Clin Pharmacol. 1979;19:571-8 | | 5 |
| e438 | Cremonesi G, Cavalieri L, Cikes I, Dobovisek J, Bacchelli S, Degli Esposti D, Costa FV, Borghi C, Ambrosioni E. Fixed combinations of delapril plus indapamide vs fosinopril plus hydrochlorothiazide in mild to moderate essential hypertension. Adv Ther. 2002;19:129-37 | | 5 |
| e439 | Plante GE, Dessurault DL. Hypertension in elderly patients. A comparative study between indapamide and hydrochlorothiazide. Am J Med. 1988;84:98-103 | | 5 |
| e440 | Pasquel R, Tribble PW, Simon A. Hypotensive effects of xipamide in essential hypertension. Crossover comparison with hydrochlorothiazide. J Clin Pharmacol. 1981;21:316-22 | | 5 |
| e441 | Plante GE, Robillard C. Indapamide in the treatment of essential arterial hypertension: results of a controlled study. Curr Med Res Opin. 1983;8 Suppl 3:59-66 | | 5 |
| e442 | Schmieder RE, Philipp T, Guerediaga J, Gorostidi M, Smith B, Weissbach N, Maboudian M, Botha J, van Ingen H. Long-term antihypertensive efficacy and safety of the oral direct renin inhibitor aliskiren: a 12-month randomized, double-blind comparator trial with hydrochlorothiazide. Circulation. 2009;119:417-25 | | 5 |
| e443 | Belleau LJ, Lebel M, Brossard JJ. Merits of adding a beta blocker (acebutolol) to a diuretic (hydrochlorothiazide) in the treatment of hypertension. J Clin Pharmacol. 1982;22:20-7 | | 5 |
| e444 | Van der Does R, Widmann L, Uberbacher HJ, Hörrmann M, Machwirth M, Stienen U. Efficacy and safety of carvedilol in comparison with atenolol in hypertensive patients pretreated with hydrochlorothiazide. Eur J Clin Pharmacol. 1990;38 Suppl 2:S147-52 | | 5 |
| e445 | Salvetti A, Magagna A, Innocenti P, Ponzanelli F, Cagianelli A, Cipriani M, Gandolfi E, Del Prato C, Ballestra AM, Saba P, et al. The combination of chlorthalidone with nifedipine does not exert an additive antihypertensive effect in essential hypertensives: a crossover multicenter study. J Cardiovasc Pharmacol. 1991;17:332-5 | | 5 |
| e446 | Tomlinson B, Woo J, Thomas GN, Chau YM, Critchley JA. Randomized, controlled, parallel-group comparison of ambulatory and clinic blood pressure responses to amlodipine or enalapril during and after treatment in adult chinese patients with hypertension. Clin Ther. 2004;26:1292-304 | | 5, 2 |
| e447 | Sharma A, Bagchi A, Kinagi SB, Sharma YK, Baliga VP, Bollmall C. Results of a comparative, phase III, 12-week, multicenter, prospective, randomized, double-blind assessment of the efficacy and tolerability of a fixed-dose combination of telmisartan and amlodipine versus amlodipine monotherapy in Indian adults with stage II hypertension. Clin Ther. 2007;29:2667-76 | | 5 |
| e448 | Verdecchia P, Calvo C, Möckel V, Keeling L, Satlin A. Safety and efficacy of the oral direct renin inhibitor aliskiren in elderly patients with hypertension. Blood Press. 2007;16:381-91 | | 5 |
| e449 | Espinel CH, Williams JL, Coughlin SS. Enalapril and lisinopril in the treatment of mild to moderate essential hypertension. Clin Ther. 1990;12:181-90 | | 5, 3 |
| e450 | Frishman WH, Goldberger J, Sherman D. Enalapril, hydrochlorothiazide, and combination therapy in patients with moderate hypertension. J Clin Hypertens. 1987;3:520-7 | | 5 |
| e451 | Mehta J, Lopez LM, Thorman AD. Lisinopril versus lisinopril plus hydrochlorothiazide in essential hypertension. Am J Cardiol. 1988;61:803-6 | | 5 |
| e452 | Neldam S, Edwards C; Telmisartan/Hydrochlorothiazide Investigators. Results of increasing doses of hydrochlorothiazide in combination with an angiotensin receptor blocker in patients with uncontrolled hypertension. J Clin Hypertens (Greenwich). 2008;10:612-8 | | 5 |
| e453 | Neldam S, Edwards C, Jones R; TEAMSTA-10 Investigators. Switching patients with uncontrolled hypertension on amlodipine 10 mg to single-pill combinations of telmisartan and amlodipine: results of the TEAMSTA-10 study. Curr Med Res Opin. 2011;27:2145-53 | | 5 |
| e454 | The Dutch TIA Trial Study Group. Trial of secondary prevention with atenolol after transient ischemic attack or nondisabling ischemic stroke Stroke. 1993;24:543-8 | | 2, 4 |
| e455 | Saunders E, Smith WB, DeSalvo KB, Sullivan WA. The efficacy and tolerability of nebivolol in hypertensive African American patients. J Clin Hypertens (Greenwich). 2007;9:866-75 | | 1 |
| e456 | Sinkiewicz W, Glazer RD, Kavoliuniene A, Miglinas M, Prak H, Wernsing M, Yen J. Efficacy and tolerability of amlodipine/valsartan combination therapy in hypertensive patients not adequately controlled on valsartan monotherapy. Curr Med Res Opin. 2009;25:315-24 | | 2 |
| e457 | Sachse A, Verboom CN, Jäger B. Efficacy of eprosartan in combination with HCTZ in patients with essential hypertension. J Hum Hypertens. 2002;16:169-76 | | 2 |
| e458 | Byyny RL, Merrill DD, Bradstreet TE, Sweet CS. An inpatient trial of the safety and efficacy of losartan compared with placebo and enalapril in patients with essential hypertension. Cardiovasc Drugs Ther. 1996;10:313-9 | | 3 |
| e459 | Frishman WH, Michelson EL, Johnson BF, Poland MP. Multiclinic comparison of labetalol to metoprolol in treatment of mild to moderate systemic hypertension. Am J Med. 1983;75:54-67 | | 1 |
| e460 | Neldam S, Lang M, Jones R; TEAMSTA-5 Investigators. Telmisartan and amlodipine single-pill combinations vs amlodipine monotherapy for superior blood pressure lowering and improved tolerability in patients with uncontrolled hypertension: results of the TEAMSTA-5 study. J Clin Hypertens (Greenwich). 2011;13:459-66 | | 2 |
| e461 | Mahmud A, Feely J. Low-dose quadruple antihypertensive combination: more efficacious than individual agents--a preliminary report. Hypertension. 2007;49:272-5 | | 1 |
| e462 | Lyngstam O, Rydén L. Metoprolol and atenolol administered once daily in primary hypertension. A clinical comparison of the efficacy of two selective beta-adrenoceptor blocking agents. Acta Med Scand. 1981;209:261-6 | | 3 |
| e463 | Martin A, Browning RC. Metoprolol in the aged hypertensive: a comparison of two dosage schedules. Postgrad Med J. 1985;61:225-7 | | 1 |
| e464 | Houston MC, Olafsson L, Burger MC. Effects of nifedipine GITS and atenolol monotherapy on serum lipids, blood pressure, heart rate, and weight in mild to moderate hypertension. Angiology. 1991;42:681-90 | | 1 |
| e465 | Mugellini A, Dobovisek J, Planinc D, Cremonesi G, Fogari R. Efficacy and safety of delapril plus manidipine compared with enalapril plus hydrochlorothiazide in mild to moderate essential hypertension: results of a randomized trial. Clin Ther. 2004;26:1419-26 | | 3 |
| e466 | Anavekar SN, Ludbrooke A, Louis WJ, Doyle AE. Evaluation of indapamide in the treatment of hypertension. J Cardiovasc Pharmacol. 1979;1:389-94 | | 1 |
| e467 | Stanton A, Jensen C, Nussberger J, O'Brien E. Blood pressure lowering in essential hypertension with an oral renin inhibitor, aliskiren. Hypertension. 2003;42:1137-43 | | 1, 3 |
| e468 | Villamil AS, Cairns V, Witte PU, Bertolasi CA. A double-blind study to compare the efficacy, tolerance and safety of two doses of the angiotensin converting enzyme inhibitor ramipril with placebo. Am J Cardiol. 1987;59:110D-114D | | 1 |
| e469 | Moser M, Lunn J. Responses to captopril and hydrochlorothiazide in black patients with hypertension. Clin Pharmacol Ther. 1982;32:307-12 | | 1 |
| e470 | Lee HY, Hong BK, Chung WJ, Lee BK, Lee SH, Jeon DW, Ahn YK, Kim D, Park CK, Kim SH, Jung HO, Kim BO, Choi D. Phase IV, 8-week, multicenter, randomized, active treatment-controlled, parallel group, efficacy, and tolerability study of high-dose candesartan cilexetil combined with hydrochlorothiazide in Korean adults with stage II hypertension. Clin Ther. 2011;33:1043-56 | | 1 |
| e471 | Reynaert J. Once-a-day treatment with sotalol and hydrochlorothiazide in patients with essential hypertension. J Clin Pharmacol. 1979;19:579-83 | | 1 |
| e472 | Jansen RW, Van Lier HJ, Hoefnagels WH. Nitrendipine versus hydrochlorothiazide in hypertensive patients over 70 years of age. Clin Pharmacol Ther. 1989;45:291-8 | | 1 |
| e473 | Bursztyn M, Ghanem J, Kobrin I, Fidel J, Ben-Ishay D. Comparison of verapamil and captopril in elderly hypertensive subjects: results of a randomized, double-blind, crossover study. J Cardiovasc Pharmacol. 1993;21:84-8 | | 1, 5 |
| e474 | Andersen K, Weinberger MH, Egan B, Constance CM, Wright M, Lukashevich V, Keefe DL. Comparative efficacy of aliskiren monotherapy and ramipril monotherapy in patients with stage 2 systolic hypertension: subgroup analysis of a double-blind, active comparator trial. Cardiovasc Ther. 2010;28:344-9 | | 1 |
| e475 | Ram CV, Ames RP, Applegate WB, Burris JF, Davidov ME, Mroczek WJ. Double-blind comparison of amlodipine and hydrochlorothiazide in patients with mild to moderate hypertension. Clin Cardiol. 1994;17:251-6 | | 1, 3 |
| e476 | Bateman DN, Dean CR, Mucklow JC, Bulpitt CJ, Dollery CT. Atenolol and chlorthalidone in combination for hypertension. Br J Clin Pharmacol. 1979;7:357-63 | | 1 |
| e477 | Backhouse CI, Hosie J, Tweed JA, Edwards KG. Atenolol and chlorthalidone in combination in the management of older hypertensive patients: a randomized clinical trial. Curr Med Res Opin. 1985;9:378-83 | | 1 |
| e478 | Mroczek WJ. Efficacy and safety of a new angiotensin-converting enzyme inhibitor, ramipril, vs. enalapril in essential hypertension: a multicenter trial. J Cardiovasc Pharmacol. 1991;18 Suppl 2:S147-9 | | 1 |
| e479 | Krum H, Nolly H, Workman D, He W, Roniker B, Krause S, Fakouhi K. Efficacy of eplerenone added to renin-angiotensin blockade in hypertensive patients. Hypertension. 2002;40:117-23 | | 1 |
| e480 | Maclean D, Ramsay LE, Richardson PJ. Enalapril and nifedipine in the treatment of mild to moderate essential hypertension: a 6 month comparison. Br J Clin Pharmacol. 1990;30:203-11 | | 1 |
| e481 | Helgeland A, Strømmen R, Hagelund CH, Tretli S. Enalapril, atenolol, and hydrochlorothiazide in mild to moderate hypertension. A comparative multicentre study in general practice in Norway. Lancet. 1986;1:872-5 | | 5, 1 |
| e482 | Jackson B, Morgan TO, Gibson J, Anderson A. Felodipine versus prazosin as an addition to a beta-blocker in the treatment of essential hypertension. The Australian Multicentre Study. Drugs. 1987;34 Suppl 3:109-19 | | 5, 1 |
| e483 | Dahlöf B, Jönsson L, Borgholst O, Ekblad G, Engstrand C, Grundestam I, Lindh A. Improved antihypertensive efficacy of the felodipine-metoprolol extended-release tablet compared with each drug alone. Blood Press Suppl. 1993;1:37-45 | | 1 |
| e484 | Van Nueten L, Schelling A, Vertommen C, Dupont AG, Robertson JI. Nebivolol vs enalapril in the treatment of essential hypertension: a double-blind randomised trial. J Hum Hypertens. 1997;11:813-9 | | 1 |
| e485 | Ninci, M. A.; Magliocca, R.; Malliani, A. Efficacy and Tolerability of Lercanidipine in Elderly Patients with Mild to Moderate Hypertension in a Placebo Controlled, Double-Blind Study. J Cardiovasc Pharmacol 1997;29: S40–S44 | | 1 |
| e486 | James IG, Jones A, Davies P. A randomised, double-blind, double-dummy comparison of the efficacy and tolerability of lercanidipine tablets and losartan tablets in patients with mild to moderate essential hypertension. J Hum Hypertens. 2002;16:605-10 | | 1 |
| e487 | De Giorgio LA, Orlandini F, Malasoma P, Zappa A. Double-blind, crossover study of lercanidipine versus amlodipine in the treatment of mild-to-moderate essential hypertension. Curr Ther Res Clin Exp 1999;60:511-20 | | 1 |
| e488 | Omboni S, Zanchetti A. Antihypertensive efficacy of lercanidipine at 2.5, 5 and 10 mg in mild to moderate essential hypertensives assessed by clinic and ambulatory blood pressure measurements. Multicenter Study Investigators. J Hypertens. 1998;16:1831-8 | | 1 |
| e489 | Lacourcière Y, Poirier L, Lefebvre J, Archambault F, Cléroux J, Boileau G. Antihypertensive effects of amlodipine and hydrochlorothiazide in elderly patients with ambulatory hypertension. Am J Hypertens. 1995;8:1154-9 | | 1, 3 |
| e490 | White WB, Sica DA, Calhoun D, Mansoor GA, Anders RJ. Preventing increases in early-morning blood pressure, heart rate, and the rate-pressure product with controlled onset extended release verapamil at bedtime versus enalapril, losartan, and placebo on arising. Am Heart J. 2002;144:657-65 | | 1 |
| e491 | Kochar M, Guthrie R, Triscari J, Kassler-Taub K, Reeves RA. Kochar M, Guthrie R, Triscari J, Kassler-Taub K, Reeves RA. Am J Hypertens. 1999;12:797-805 | | 1 |
| e492 | Daniels AR, Opie LH. Atenolol plus nifedipine for mild to moderate systemic hypertension after fixed doses of either agent alone. Am J Cardiol. 1986;57:965-70 | | 1, 3 |
| e493 | Dahlöf C, Hedner T, Thulin T, Gustafsson S, Olsson SO. Effects of diltiazem and metoprolol on blood pressure, adverse symptoms and general well-being. The Swedish Diltiazem-Metoprolol Multi-Centre Study Group..Eur J Clin Pharmacol. 1991;40:453-60 | | 1, 3 |
| e494 | Edmonds D, Greminger P, Locher R, Knorr M, Vetter H, Vetter W. Enalapril as a first-step agent in essential hypertension: a comparative study with atenolol. J Hypertens Suppl. 1986;4:S406-9 | | 1 |
| e495 | Djian J, Roy M, Forette B, Lekieffre J, Luccioni R. Efficacy and tolerance of sustained-release diltiazem 300 mg and a diuretic in the elderly. J Cardiovasc Pharmacol. 1990;16 Suppl 1:S51-5 | | 1 |
| e496 | Burris JF, Weir MR, Oparil S, Weber M, Cady WJ, Stewart WH. An assessment of diltiazem and hydrochlorothiazide in hypertension. Application of factorial trial design to a multicenter clinical trial of combination therapy. JAMA. 1990;263:1507-12 | | 1 |
| e497 | Coca A, Calvo C, García-Puig J, Gil-Extremera B, Aguilera MT, de la Sierra A, Martín-Hidalgo A, Marín R; MAPAVEL Investigators (Monitorizacíon Ambulatoria Presión Arterial APROVEL). A multicenter, randomized, double-blind comparison of the efficacy and safety of irbesartan and enalapril in adults with mild to moderate essential hypertension, as assessed by ambulatory blood pressure monitoring: the MAPAVEL Study (Monitorización Ambulatoria Presión Arterial APROVEL). Clin Ther. 2002;24:126-38 | | 1 |
| e498 | Myers MG, de Champlain J. Effects of atenolol and hydrochlorothiazide on blood pressure and plasma catecholamines in essential hypertension. Hypertension. 1983;5:591-6 | | 1 |
| e499 | Cowling CG, Leary WP, Mahomedy Y, van der Byl K. Antihypertensive effects of acebutolol plus hydrochlorothiazide and hydrochlorothiazide alone in black patients. S Afr Med J. 1986;70:92-4 | | 1 |
| e500 | Fodor JG, Chockalingam A, Cifkova R, Strong HA, Cobby J, Mukherjee J. Efficacy of once daily nitrendipine in mild hypertension: comparison withplacebo .Can J Cardiol. 1991;7:223-8 | | 1 |
| e501 | Fogari R, Mugellini A, Preti P, et al. Valsartan addition to amlodipine is more effective than losartan addition in hypertensive patients inadequately controlled by amlodipine.Vasc Health Risk Manag 2010.:87-93 | | 1 |
| e502 | Chrysant SG. Perindopril/hydrochlorothiazide dose combinations for the treatment of hypertension: a multicenter study. J Clin Pharmacol. 1997;37:47-52 | | 1, 3 |
| e503 | Shapiro DA, Liss CL, Walker JF, Lewis JL, Lengerich RA, Irvin JD. Enalapril and hydrochlorothiazide as antihypertensive agents in the elderly. J Cardiovasc Pharmacol. 1987;10 Suppl 7:S160-2 | | 1 |
| e504 | McGill JB, Reilly PA. Telmisartan plus hydrochlorothiazide versus telmisartan or hydrochlorothiazide monotherapy in patients with mild to moderate hypertension: a multicenter, randomized, double-blind, placebo-controlled, parallel-group trial. Clin Ther. 2001;23:833-50 | | 1 |
| e505 | McInnes GT, O'Kane KP, Istad H, Keinänen-Kiukaanniemi S, Van Mierlo HF. Comparison of the AT1-receptor blocker, candesartan cilexetil, and the ACE inhibitor, lisinopril, in fixed combination with low dose hydrochlorothiazide in hypertensive patients. J Hum Hypertens. 2000;14:263-9 | | 1 |
| e506 | Andreadis EA, Tsourous GI, Marakomichelakis GE, Katsanou PM, Fotia ME, Vassilopoulos CV, Diamantopoulos EJ. High-dose monotherapy vs low-dose combination therapy of calcium channel blockers and angiotensin receptor blockers in mild to moderate hypertension. J Hum Hypertens. 2005;19:491-6 | | 1 |
| e507 | Groom P, Simpson RJ, Singh B, Ward DE, Peers E, Richardson PD. A double-blind comparison of felodipine and hydrochlorothiazide added to metoprolol to control hypertension. Eur J Clin Pharmacol. 1988;34:21-4 | | 1 |
| e508 | Tuomilehto J, Tykarski A, Baumgart P, Reimund B, Le Breton S, Ferber P. Combination therapy with valsartan/hydrochlorothiazide at doses up to 320/25 mg improves blood pressure levels in patients with hypertension inadequately controlled by valsartan 320 mg monotherapy. Blood Press Suppl. 2008;1:15-23 | | 1 |
| e509 | Waeber B, Aschwanden R, Sadecky L, Ferber P. Combination of hydrochlorothiazide or benazepril with valsartan in hypertensive patients unresponsive to valsartan alone. J Hypertens. 2001;19:2097-104 | | 1 |
| e510 | Gleim GW, Rubino J, Zhang H, Shahinfar S, Soffer BA, Lyle PA, Littlejohn TW 3rd, Feig PU. A multicenter, randomized, double-blind, parallel-group trial of the antihypertensive efficacy and tolerability of a combination of once-daily losartan 100 mg/hydrochlorothiazide 12.5 mg compared with losartan 100-mg monotherapy in the treatment of mild to severe essential hypertension. Clin Ther. 2006;28:1639-48 | | 1 |
| e511 | PROGRESS Collaborative Group. Randomised trial of a perindopril-based blood-pressure-lowering regimen among 6,105 individuals with previous stroke or transient ischaemic attack. Lancet. 2001;358:1033-41 | | 1 |
| e512 | Freytag F, Schelling A, Meinicke T, Deichsel G; Telmisartan Hypertension Experience in a Randomized European Study Versus Atenolol Study Group. Comparison of 26-week efficacy and tolerability of telmisartan and atenolol, in combination with hydrochlorothiazide as required, in the treatment of mild to moderate hypertension: a randomized, multicenter study. Clin Ther. 2001;23:108-23 | | 1 |
| e513 | Mimran A, Ruilope L, Kerwin L, Nys M, Owens D, Kassler-Taub K, Osbakken M. A randomised, double-blind comparison of the angiotensin II receptor antagonist, irbesartan, with the full dose range of enalapril for the treatment of mild-to-moderate hypertension. J Hum Hypertens. 1998;12:203-8 | | 1, 3 |
| e514 | Neutel JM, Frishman WH, Oparil S, Papademitriou V, Guthrie G. Comparison of telmisartan with lisinopril in patients with mild-to-moderate hypertension. Am J Ther. 1999;6:161-6 | | 1 |
| e515 | Bolzano K, Arriaga J, Bernal R, Bernardes H, Calderon JL, Debruyn J, Dienstl F, Drayer J, Goodfriend TL, Gross W, et al. The antihypertensive effect of lisinopril compared to atenolol in patients with mild to moderate hypertension. J Cardiovasc Pharmacol. 1987;9 Suppl 3:S43-7 | | 1 |
| e516 | Weinberger MH. Comparison of captopril and hydrochlorothiazide alone and in combination in mild to moderate essential hypertension. Br J Clin Pharmacol. 1982;14 Suppl 2:127S-131S | | 1 |
| e517 | Lorimer AR, Smedsrud T, Walker P, Tyler HM. A comparison of amlodipine, verapamil and placebo in the treatment of mild to moderate hypertension. Amlodipine Study Group. J Hum Hypertens. 1989;3:191-6 | | 1 |
| e518 | Miranda RD, Mion D Jr, Rocha JC, Kohlmann O Jr, Gomes MA, Saraiva JF, Amodeo C, Filho BL. An 18-week, prospective, randomized, double-blind, multicenter study of amlodipine/ramipril combination versus amlodipine monotherapy in the treatment of hypertension: the assessment of combination therapy of amlodipine/ramipril (ATAR) study. Clin Ther. 2008;30:1618-28 | | 1 |
| e519 | Edmonds D, Knorr M, Greminger P, Walger P, Frielingsdorf J, Vetter H, Vetter W. ACE inhibitor versus beta-blocker in the treatment of essential hypertension.Nephron. 1987;47 Suppl 1:90-3 | | 1 |
| e520 | Neutel JM, Franklin SS, Oparil S, Bhaumik A, Ptaszynska A, Lapuerta P. Efficacy and safety of irbesartan/HCTZ combination therapy as initial treatment for rapid control of severe hypertension. J Clin Hypertens (Greenwich). 2006;8:850-7 | | 1 |
| e521 | Marre M, Puig JG, Kokot F, Fernandez M, Jermendy G, Opie L, Moyseev V, Scheen A, Ionescu-Tirgoviste C, Saldanha MH, Halabe A, Williams B, Mion Júnior D, Ruiz M, Hermansen K, Tuomilehto J, Finizola B, Gallois Y, Amouyel P, Ollivier JP, Asmar R. Equivalence of indapamide SR and enalapril on microalbuminuria reduction in hypertensive patients with type 2 diabetes: the NESTOR Study. J Hypertens. 2004;22:1613-22 | | 1 |
| e522 | London GM. Efficacy of indapamide 1.5 mg, sustained release, in the lowering of systolic blood pressure. J Hum Hypertens. 2004;18 Suppl 2:S9-S14 | | 1 |
| e523 | Schrader J, Lüders S, Kulschewski A, Hammersen F, Plate K, Berger J, Zidek W, Dominiak P, Diener HC; MOSES Study Group. Morbidity and Mortality After Stroke, Eprosartan Compared with Nitrendipine for Secondary Prevention: principal results of a prospective randomized controlled study (MOSES). Stroke. 2005;36:1218-26 | | 1, 3 |
| e524 | Schrader J, Lüders S, Kulschewski A, Berger J, Zidek W, Treib J, Einhäupl K, Diener HC, Dominiak P; Acute Candesartan Cilexetil Therapy in Stroke Survivors Study Group. The ACCESS Study: evaluation of Acute Candesartan Cilexetil Therapy in Stroke Survivors. Stroke. 2003;34:1699-703 | | 1, 3 |
| e525 | Gosse P, Sheridan DJ, Zannad F, Dubourg O, Guéret P, Karpov Y, de Leeuw PW, Palma-Gamiz JL, Pessina A, Motz W, Degaute JP, Chastang C. Regression of left ventricular hypertrophy in hypertensive patients treated with indapamide SR 1.5 mg versus enalapril 20 mg: the LIVE study. J Hypertens. 2000;18:1465-75 | | 1, 3 |
| e526 | Messerli F, Frishman WH, Elliott WJ, Bacher PH, Pepine CJ. Antihypertensive properties of a high-dose combination of trandolapril and verapamil-SR. Blood Press Suppl. 2007 Mar;1:6-9 | | 1 |
| e527 | Benz JR, Black HR, Graff A, Reed A, Fitzsimmons S, Shi Y. Valsartan and hydrochlorothiazide in patients with essential hypertension. A multiple dose, double-blind, placebo controlled trial comparing combination therapy with monotherapy. J Hum Hypertens. 1998;12:861-6 | | 1 |
| e528 | Morgan T, Anderson A. Clinical efficacy of perindopril in hypertension. Clin Exp Pharmacol Physiol Suppl. 1992;19:61-5 | | 1, 5 |
| e529 | Wahl J, Turlapaty P, Singh BN. Comparison of acebutolol and propranolol in essential hypertension. Am Heart J. 1985;109:313-21 | | 1 |
| e530 | Mancia G, Brown M, Castaigne A, de Leeuw P, Palmer CR, Rosenthal T, Wagener G, Ruilope LM; INSIGHT. Outcomes with nifedipine GITS or Co-amilozide in hypertensive diabetics and nondiabetics in Intervention as a Goal in Hypertension (INSIGHT). Hypertension. 2003;4:431-6 | | 1 |
| e531 | Bremner AD, Baur M, Oddou-Stock P, Bodin F. Valsartan: long-term efficacy and tolerability compared to lisinopril in elderly patients with essential hypertension. Clin Exp Hypertens. 1997;19:1263-85 | | 1 |
| e532 | Hosie J, Dahlöf B, Klein G. The long-term antihypertensive efficacy and safety of a new felodipine-metoprolol combination tablet. The Swedish/UK and German Study Groups. Blood Press Suppl. 1993;1:46-50 | | 1, 5 |
| e533 | Thurston H, Desche P. Assessment of antihypertensive efficacy of perindopril: results of double-blind multicenter studies versus reference drugs. J Cardiovasc Pharmacol. 1991;18 Suppl 7:S45-9 | | 1 |
| e534 | Mourad JJ, Lameira D, Guillausseau PJ. Blood pressure normalization by fixed perindopril/indapamide combination in hypertensive patients with or without associate metabolic syndrome: results of the OPTIMAX 2 study. Vasc Health Risk Manag. 2008;4:443-51 | | 1 |
| e535 | Hedner T, Thulin T, Gustafsson S, Olsson SO. A comparison of diltiazem and metoprolol in hypertension. Swedish Diltiazem-Metoprolol Multicentre Study Group. Eur J Clin Pharmacol. 1990;39:427-33 | | 1 |
| e536 | Multicenter Diuretic Cooperative Study Group no authors listed). Multiclinic comparison of amiloride, hydrochlorothiazide, and hydrochlorothiazide plus amiloride in essential hypertension. Arch Intern Med. 1981;14:482-6 | | 1 |
| e537 | Lacourcière Y, Bélanger A, Godin C, Hallé JP, Ross S, Wright N, Marion J. Long-term comparison of losartan and enalapril on kidney function in hypertensive type 2 diabetics with early nephropathy. Kidney Int. 2000;58:762-9 | | 1 |
| e538 | Weiss RJ, Weber MA, Carr AA, Sullivan WA. A randomized, double-blind, placebo-controlled parallel-group study to assess the efficacy and safety of nebivolol, a novel beta-blocker, in patients with mild to moderate hypertension. J Clin Hypertens (Greenwich). 2007;9:667-76 | | 1 |
| e539 | Massie B, MacCarthy EP, Ramanathan KB, Weiss RJ, Anderson M, Eidelson BA, Labreche DG, Tubau JF, Ulep D, Bartels D. Diltiazem and propranolol in mild to moderate essential hypertension as monotherapy or with hydrochlorothiazide. Ann Intern Med. 1987;107:150-7 | | 1, 3 |
| e540 | Phillips RA, Kloner RA, Grimm RH Jr, Weinberger M. The effects of amlodipine compared to losartan in patients with mild to moderately severe hypertension. J Clin Hypertens (Greenwich). 2003;5:17-23 | | 1, 3 |
| e541 | Williams GH, Burgess E, Kolloch RE, Ruilope LM, Niegowska J, Kipnes MS, Roniker B, Patrick JL, Krause SL. Efficacy of eplerenone versus enalapril as monotherapy in systemic hypertension. Am J Cardiol. 2004;93:990-6 | | 1, 3 |
| e542 | Weir MR, Elkins M, Liss C, Vrecenak AJ, Barr E, Edelman JM. Efficacy, tolerability, and quality of life of losartan, alone or with hydrochlorothiazide, versus nifedipine GITS in patients with essential hypertension. Clin Ther. 1996;18:411-28 | | 1, 3 |
| e543 | Tedesco MA, Natale F, Calabrò R. Effects of monotherapy and combination therapy on blood pressure control and target organ damage: a randomized prospective intervention study in a large population of hypertensive patients. J Clin Hypertens (Greenwich). 2006;8:634-41 | | 1 |
| e544 | Wheeldon NM, MacDonald TM, Prasad N, Maclean D, Peebles L, McDevitt DG. A double-blind comparison of bisoprolol and atenolol in patients with essential hypertension. QJM. 1995;88:565-70 | | 1 |
| e545 | Walle PO, Westergren G, Dimenäs E, Olofsson B, Albrektsen T. Effects of 100 mg of controlled-release metoprolol and 100 mg of atenolol on blood pressure, central nervous system-related symptoms, and general well being. J Clin Pharmacol. 1994;34:742-7 | | 1 |
| e546 | Johnson SM, Mauritson DR, Corbett JR, Woodward W, Willerson JT, Hillis LD. Double-blind, randomized, placebo-controlled comparison of propranolol and verapamil in the treatment of patients with stable angina pectoris. Am J Med. 1981;71:443-51 | | 1, 5 |
| e547 | Grassi G, Seravalle G, Dell'Oro R, Trevano FQ, Bombelli M, Scopelliti F, Facchini A, Mancia G; CROSS Study. Comparative effects of candesartan and hydrochlorothiazide on blood pressure, insulin sensitivity, and sympathetic drive in obese hypertensive individuals: results of the CROSS study. J Hypertens. 2003;21:1761-9 | | 1 |
| e548 | Massie BM, Tubau JF, Szlachcic J, Vollmer C. Comparison and additivity of nitrendipine and hydrochlorothiazide in systemic hypertension. Am J Cardiol. 1986;58:16D-19D | | 1 |
| e549 | Hort JF, Wilkins HM.Changes in blood pressure, serum potassium and electrolytes with a combination of triamterene and a low dose of chlorthalidone. Curr Med Res Opin. 1991;12:430-40 | | 1 |
| e550 | Frishman WH, Garofalo JL, Rothschild A, Rothschild M, Greenberg SM, Soberman J. Multicenter comparison of the nifedipine gastrointestinal therapeutic system and long-acting propranolol in patients with mild to moderate systemic hypertension receiving diuretics. A preliminary experience. Am J Med. 1987;83:15-9 | | 1 |
| e551 | Yener G, Aran S, Bahceci M, Ozdemir K, Gultekin F, Lowe W. Quinapril for treatment of hypertension in Turkey: dose titration and diuretic combination treatment strategies. Clin Drug Investig. 2007;27:613-22 | | 1 |
| e552 | Medina-Ruiz A, Feliu JF. A cooperative study to evaluate the efficacy and safety of enalapril in Puerto Rican patients with mild to moderate hypertension. Drugs. 1990;39 Suppl 2:77-82 | | 1 |
| e553 | Messerli FH, Weir MR, Neutel JM. Combination therapy of amlodipine/benazepril versus monotherapy of amlodipine in a practice-based setting. Am J Hypertens. 2002;15:550-6 | | 1 |
| e554 | Yener G, Aran S, Bahceci M, Ozdemir K, Gultekin F, Lowe W. Quinapril for treatment of hypertension in Turkey: dose titration and diuretic combination treatment strategies. Clin Drug Investig. 2007;27:613-22 | | 1 |
| e555 | Backhouse CI, Orofiamma B, Pauly NC. Long-term therapy with trandolapril, a new nonsulfhydryl ACE inhibitor, in hypertension: a multicenter international trial. Investigator Study Group. J Cardiovasc Pharmacol. 1994;23 Suppl 4:S86-90 | | 1 |
| e556 | MRC Working Party. Medical Research Council trial of treatment of hypertension in older adults: principal results. BMJ.1992; 304: 405-412 | | 1 |
| e557 | Medical Research Council Working Party. MRC trial of treatment of mild hypertension: principal results. Br Med J (Clin Res Ed). 1985;291:97-104 | | 1 |
| e558 | Suraniti S, Berrut G, Marre M, Fressinaud P. Antihypertensive efficacy and acceptability of perindopril in elderly hypertensive patients. Am J Cardiol. 1993;71:28E-31E | | 1 |
| e559 | Grimbert P, Grenier O, Thuillez C; APPROVE Study Investigators. Dose-related efficacy of irbesartan in patients, with mild-to-moderate essential hypertension. Therapie. 2005;60:577-82 | | 1 |
| e560 | Rump LC, Girerd X, Sellin L, Stegbauer J. Effects of high dose olmesartan medoxomil plus hydrochlorothiazide on blood pressure control in patients with grade 2 and grade 3 hypertension. J Hum Hypertens. 2011;25:565-74 | | 1 |
| e561 | Muiesan G, Agabiti-Rosei E, Carotti A, Corea L, Innocenti P, Montervino C, Prezioso R, Romanelli G, Toso M, Motolese M. Antihypertensive activity of once daily metoprolol alone and with chlorthalidone and comparison with a twice daily regimen. Eur J Clin Pharmacol. 1982;23:209-13 | | 1 |
| e562 | Gradman AH, Cutler NR, Davis PJ, Robbins JA, Weiss RJ, Wood BC, Michelson EL. Long-term efficacy, tolerability, and safety of the combination of enalapril and felodipine ER in the treatment of hypertension. Clin Ther. 1998;20:527-38 | | 1 |
| e563 | Chrysant SG, Murray AV, Hoppe UC, Dattani D, Patel S, Hsu H, Zhang J. Long-term safety, tolerability and efficacy of aliskiren in combination with valsartan in patients with hypertension: a 6-month interim analysis. Curr Med Res Opin. 2008;24:1039-47 | | 1 |
| e564 | Ostman J, Asplund K, Bystedt T, Dahlöf B, Jern S, Kjellström T, Lithell H. Comparison of effects of quinapril and metoprolol on glycaemic control, serum lipids, blood pressure, albuminuria and quality of life in non-insulin-dependent diabetes mellitus patients with hypertension. J Intern Med. 1998;24495-107 | | 2 |
| e565 | Roca-Cusachs A1, Schmieder RE, Triposkiadis F, Wenzel RR, Laurent S, Kohlmann O Jr, Fogari R; MORE Study Group. Efficacy of manidipine/delapril versus losartan/hydrochlorothiazide fixed combinations in patients with hypertension and diabetes. J Hypertens. 2008;26:813-8 | | 2 |
| e566 | Karotsis AK, Symeonidis A, Mastorantonakis SE, Stergiou GS; Home-Di-Plus Study Group. Additional antihypertensive effect of drugs in hypertensive subjects uncontrolled on diltiazem monotherapy: a randomized controlled trial using office and home blood pressure monitoring. Clin Exp Hypertens. 2006;28:655-62 | | 2 |
| e567 | Pareek A, Basavanagowdappa H, Zawar S, Kumar A, Chandurkar N. A randomized, comparative study evaluating the efficacy and tolerability of losartan-low dose chlorthalidone (6.25 mg) combination with losartan-hydrochlorothiazide (12.5 mg) combination in Indian patients with mild-to-moderate essential hypertension. Expert Opin Pharmacother. 2009;10:1529-36 | | 2 |
| e568 | Fogari R, Zoppi A,Mugellini A, Preti P, Destro M, Rinaldi A, Derosa G. Effectiveness of hydrochlorothiazide in combination with telmisartan and olmesartan in adults with moderate hypertension not controlled with monotherapy: a prospective, randomized, open-label, blinded end point (PROBE), parallel-arm study. Curr Ther Res Clin Exp. 2008;69:1 | | 5 |
| e569 | Pool JL, Schmieder RE, Azizi M, Aldigier JC, Januszewicz A, Zidek W, Chiang Y, Satlin A. Aliskiren, an orally effective renin inhibitor, provides antihypertensive efficacy alone and in combination with valsartan. Am J Hypertens. 2007;20:11-20 | | 6 |
| e570 | Applegate WB, Phillips HL, Schnaper H, Shepherd AM, Schocken D, Luhr JC, Koch GG, Park GD. A randomized controlled trial of the effects of three antihypertensive agents on blood pressure control and quality of life in older women. Arch Intern Med. 1991 ;151:1817-23 | | 3 |
| e571 | Fogari R, Zoppi A, Mugellini A, Preti P, Destro M, Rinaldi A, Derosa G. [Hydrochlorothiazide added to valsartan is more effective than when added to HYPERLINK "http://www.ncbi.nlm.nih.gov/pubmed/17142202"olmesartanHYPERLINK "http://www.ncbi.nlm.nih.gov/pubmed/17142202" in reducing blood pressure in moderately hypertensive patients inadequately controlled by monotherapyHYPERLINK "http://www.ncbi.nlm.nih.gov/pubmed/17142202".](http://www.ncbi.nlm.nih.gov/pubmed/17142202) Adv Ther. 2006 ;23:680-95 | | 5 |
| e572 | Wilson TW, Lacourcière Y, Barnes CC. The antihypertensive efficacy of losartan and amlodipine assessed with office and ambulatory blood pressure monitoring. CMAJ. 1998;159:469-76 | | 3 |
| e573 | Fagan TC. Blood pressure reduction and tolerability of felodipine ER in older and younger hypertensive patients. J Am Geriatr Soc. 1997;45:712-7 | | 6 |
| e574 | Calvo C, Hermida RC, Ayala DE, Ruilope LM. Effects of telmisartan 80 mg and valsartan 160 mg on ambulatory blood pressure in patients with essential hypertension. J Hypertens. 2004;22:837-46 | | 5 |
| e575 | Høegholm A, Wiinberg N, Rasmussen E, Nielsen PE. Office and ambulatory blood pressure: a comparison between amlodipine and felodipine ER. J Hum Hypertens. 1995;9:611-6 | | 3 |
| e576 | Neutel JM, Littlejohn TW, Chrysant SG, Singh A; Telmisartan Study Group. Telmisartan/Hydrochlorothiazide in comparison with losartan/hydrochlorothiazide in managing patients with mild-to-moderate hypertension. Hypertens Res. 2005;28:555-63 | | 5 |
| e577 | Manolis AJ, Grossman E, Jelakovic B, Jacovides A, Bernhardi DC, Cabrera WJ, Watanabe LA, Barragan J, Matadamas N, Mendiola A, Woo KS, Zhu JR, Mejia AD, Bunt T, Dumortier T, Smith RD. Effects of losartan and candesartan monotherapy and losartan/hydrochlorothiazide combination therapy in patients with mild to moderate hypertension. Clin Ther. 2000;22:1186-203 | | 3 |
| e578 | Pool J, Kaihlanen P, Lewis G, Ginsberg D, Oparil S, Glazer R, Messerli FH. Once-daily treatment of patients with hypertension: a placebo-controlled study of amlodipine and benazepril vs amlodipine or benazepril alone. J Hum Hypertens. 2001;15:495-8 | | 3 |
| e579 | National Intervention Cooperative Study in Elderly Hypertensives Study Group. Randomized double-blind comparison of a calcium antagonist and a diuretic in elderly hypertensives. Hypertension 1999;34:1129-33 | | 2 |
| e580 | Obel Arthur O. Efficay and Tolerability of Long Term Oxprenolol and Chlorthalidone Singly and in Combination in Hypertensive Blacks. Jpn. Heart J. 1990; 31:183-192 | |  |
| e581 | Massie BM. Antihypertensive therapy with nitrendipine: comparison with hydrochlorothiazide and propranolol. J Cardiovasc Pharmacol. 1988;12 Suppl 4:S55-8 | | 3 |
| e582 | Moncloa F, Hwang IK, Muccilli AC. Multiclinic evaluation of the antihypertensive effect of a methyldopa, hydrochlorothiazine, and amiloride combination. Clin Ther. 1980;3:168-75 | | 6 |
| e583 | Pareek A, Salkar H, Mulay P, Desai S, Chandurkar N, Redkar N. A randomized, comparative, multicentric evaluation of atenolol/amlodipine combination with atenolol alone in essential hypertensive patients. Am J Ther. 2010;17:46-52 | | 2 |
| e584 | Weber MA, Sica DA, Tarka EA, Iyengar M, Fleck R, Bakris GL. Controlled-release carvedilol in the treatment of essential hypertension. Am J Cardiol. 2006;98:32L-38L | | 3 |
| e585 | Hoffmann J. Comparison of a felodipine-metoprolol combination tablet vs each component alone as antihypertensive therapy. Blood Press Suppl. 1993;1:30-6 | | 6 |
| e586 | Neutel J, Elliott WJ, Izzo J, Chen CL, Masonson H. Antihypertensive efficacy of olmesartan medoxomil, a new angiotensin II receptor antagonist, as assessed by ambulatory blood pressure measurements. J Clin Hypertens (Greenwich). 2002 ;4:325-31. Només ABPM. No dades de PA basal | | 6 |
| e587 | Weber MA, Bakris GL, Tarka EA, Iyengar M, Fleck R, Sica DA. Efficacy of a once-daily formulation of carvedilol for the treatment of hypertension. J Clin Hypertens (Greenwich). 2006 ;8:840-9 | | 3 |
